# Curriculum Klinische Akut- und Notfallmedizin – Schwerpunkt Innere Medizin: Empfehlung zu Weiterbildungsinhalten der Inneren Medizin in der Notaufnahme

**DOI:** 10.1007/s00063-024-01113-3

**Published:** 2024-04-16

**Authors:** DGIIN, DGIM, BDI, DGA, DGE, DGVS, DGG, DGHO, DGI, DGK, DGfN, DG f. Pneumologie und Beatmungsmedizin, DG f. Palliativmedizin, DGRh, Hans-Jörg Busch, Sebastian Wolfrum, Guido Michels, Matthias Baumgärtel, Klaus-Friedrich Bodmann, Michael Buerke, Volker Burst, Philipp Enghard, Georg Ertl, Wolf Andreas Fach, Frank Hanses, Hans Jürgen Heppner, Carsten Hermes, Uwe Janssens, Stefan John, Christian Jung, Christian Karagiannidis, Michael Kiehl, Stefan Kluge, Alexander Koch, Matthias Kochanek, Peter Korsten, Philipp M. Lepper, Martin Merkel, Ursula Müller-Werdan, Martin Neukirchen, Alexander Pfeil, Reimer Riessen, Wolfgang Rottbauer, Sebastian Schellong, Alexandra Scherg, Daniel Sedding, Katrin Singler, Marcus Thieme, Christian Trautwein, Carsten Willam, Karl Werdan

**Affiliations:** 1 Zentrum für Notfall- und Rettungsmedizin, Universitäts-Notfallzentrum Freiburg, Universitätsklinikum Freiburg, Freiburg, Deutschland; 2 Interdisziplinäre Notaufnahme Campus Lübeck, Universitätsklinikum Schleswig-Holstein, Lübeck, Deutschland; 3 Notfallzentrum, Krankenhaus der Barmherzigen Brüder Trier, Medizincampus der Universitätsmedizin Mainz, Trier, Deutschland; 4 Klinikum Nürnberg, Universitätsklinik für Innere Medizin 3 der Paracelsus Medizinischen Privatuniversität, Nürnberg, Deutschland; 5 Ltd. Arzt Infektiologie, Kliniken Nordoberpfalz AG, Klinikum Weiden, Weiden, Deutschland; 6 Medizinische Klinik II, St. Marien-Krankenhaus Siegen, Siegen, Deutschland; 7 Schwerpunkt Klinische Akut- und Notfallmedizin und Klinik II für Innere Medizin, Uniklinik Köln, Köln, Deutschland; 8 Klinik mit Schwerpunkt Nephrologie und Internistische Intensivmedizin, Charité Universitätsmedizin, Berlin, Deutschland; 9 Deutsches Zentrum für Herzinsuffizienz, Universitätsklinikum Würzburg, Würzburg, Deutschland; 10 MVZ CCB am AGAPLESION Bethanien Krankenhaus, Frankfurt (Main), Deutschland; 11 Interdisziplinäre Notaufnahme, Universitätsklinikum Regensburg, Regensburg, Deutschland; 12 Klinik für Geriatrie und Geriatrische Tagesklinik, Klinikum Bayreuth – Medizincampus Oberfranken und Friedrich-Alexander-Universität Erlangen-Nürnberg, Bayreuth, Deutschland; 13 Bonn, Deutschland; 14 Klinik für Innere Medizin und Internistische Intensivmedizin, St-Antonius-Hospital gGmbH, Eschweiler, Deutschland; 15 Medizinische Klinik 8, Abteilung für Internistische Intensivmedizin, Klinikum Nürnberg-Süd, Paracelsus Medizinische Privatuniversität, Nürnberg, Deutschland; 16 Klinik für Kardiologie, Pneumologie und Angiologie des Universitätsklinikums Düsseldorf, Heinrich-Heine Universität Düsseldorf, Düsseldorf, Deutschland; 17 ARDS und ECMO Zentrum Köln-Merheim, Kliniken Köln und Universität Witten/Herdecke, Köln, Deutschland; 18 Medizinische Klinik I, Klinikum Frankfurt (Oder), Frankfurt (Oder), Deutschland; 19 Klinik für Intensivmedizin, Universitätsklinikum Hamburg-Eppendorf, Hamburg, Deutschland; 20 Medizinische Klinik III, Universitätsklinikum Aachen, Aachen, Deutschland; 21 Klinik I für Innere Medizin, Uniklinik Köln, Köln, Deutschland; 22 Klinik für Rheumatologie und Klinische Immunologie, St. Josef-Stift Sendenhorst, Sendenhorst, Deutschland; 23 Klinik für Akut- und Notfallmedizin, Universität und Universitätsklinikum des Saarlandes, Homburg, Deutschland; 24 Endokrinologikum Hamburg, Hamburg, Deutschland; 25 Medizinische Klinik für Geriatrie und Altersmedizin, der Charité – Universitätsmedizin Berlin und EGZB, Berlin, Deutschland; 26 Interdisziplinäres Zentrum für Palliativmedizin und Klinik für Anästhesiologie, Universitätsklinikum Düsseldorf, Heinrich-Heine-Universität Düsseldorf, Düsseldorf, Deutschland; 27 Klinik für Innere Medizin III, Universitätsklinikum Jena, Jena, Deutschland; 28 Internistische Intensivstation 93, Dept. f. Innere Medizin, Universitätsklinikum Tübingen, Tübingen, Deutschland; 29 Klinik für Innere Medizin II (Kardiologie, Angiologie, Pneumologie, Intensivmedizin, Sport- und Rehabilitationsmedizin), Universitätsklinikum Ulm, Ulm, Deutschland; 30 Direktorium, Städtisches Klinikum Dresden, Dresden, Deutschland; 31 PalliativQuartier Hamburg e.V., Hamburg, Deutschland; 32 Universitätsklinik und Poliklinik für Innere Medizin III, Universitätsklinikum Halle (Saale), Ernst-Grube-Straße 40, 06097 Halle (Saale), Deutschland; 33 Universitätsklinik für Innere Medizin – Geriatrie & Institut für Biomedizin des Alterns, Klinikum Nürnberg Paracelsus Medizinische Privatuniversität & Friedrich-Alexander Universität Erlangen-Nürnberg, Nürnberg & Erlangen, Deutschland; 34 Abteilung Innere Medizin und REGIOMED Gefäßzentrum, REGIOMED Klinikum Sonneberg, Sonneberg und Klinik für Innere Medizin I, Universitätsklinikum Jena, Jena, Deutschland; 35 Medizinische Klinik 4, Universitätsklinikum Erlangen, Erlangen, Deutschland; 36 Aachen, Deutschland

**Keywords:** Berufsverband Deutscher Internistinnen und Internisten (BDI), Bundesärztekammer, Deutsche Gesellschaft für Internistische Intensivmedizin und Notfallmedizin (DGIIN), Deutsche Gesellschaft für Innere Medizin (DGIM), Schockraumversorgung, Zusatz-Weiterbildung „Klinische Akut- und Notfallmedizin“, Zusatz-Weiterbildung „Notfallmedizin“, Professional Association of German Internists (BDI), German Medical Association, German Society of Medical Intensive Care and Emergency Medicine (DGIIN), German Society of Internal Medicine (DGIM), Resuscitation room management, Advanced training “Clinical Acute- and Emergency Medicine”, Advanced training “Emergency Medicine”

## Abstract

In Deutschland qualifiziert sich der Akut- und Notfallmediziner durch eine Facharztweiterbildung in Verbindung mit den Zusatz-Weiterbildungen „Klinische Akut- und Notfallmedizin“ und „Notfallmedizin“ gemäß den Vorgaben der Landesärztekammern, die sich auf die Empfehlungen der Bundesärztekammer beziehen. Eine zentrale Säule in der gebietsübergreifenden notfallmedizinischen Versorgung stellt das Gebiet der Inneren Medizin mit seinen Schwerpunkten dar. Das vorliegende Curriculum gibt einen umfassenden Überblick über internistische Weiterbildungsinhalte der Akut- und Notfallmedizin, die nach Ansicht der internistischen Gesellschaften (Deutsche Gesellschaft für Internistische Intensivmedizin und Notfallmedizin [DGIIN], Deutsche Gesellschaft für Innere Medizin [DGIM] samt Schwerpunktgesellschaften, Berufsverband Deutscher Internistinnen und Internisten [BDI]) für den Erwerb der erforderlichen Kenntnisse und praktischen Fähigkeiten für eine bestmögliche Versorgung der akut- und notfallmedizinischen Patienten aus internistischer Sicht erforderlich scheinen. Das Curriculum stellt zum einen die allgemeinen Aspekte der klinischen Akut- und Notfallmedizin mit den Inhalten Struktur- und Prozessqualität, Erstdiagnostik, Initialtherapie und Indikationsstellung zur weiterführenden Behandlung, Schockraumversorgung, Diagnostik und Monitoring, generelle Therapieverfahren, Hygienemaßnahmen und Pharmakotherapie dar. Anschließend folgen spezifische Aspekte der Akut- und Notfallmedizin (angiologische, endokrinologische, diabetologische und metabolische, gastroenterologische, geriatrische, hämatoonkologische, infektiologische, kardiologische, nephrologische, palliativmedizinische, pneumologische, rheumatologische und toxikologische). Unterlegt sind die Themen jeweils mit auf das Weiterbildungskonzept zugeschnittenen Publikationen. Das Curriculum stellt für Internistinnen und Internisten alle internistischen Weiterbildungsinhalte der o. g. Zusatz-Weiterbildungen dar, zeigt aber auch allen Notfallmedizinern, mit welchen internistischen Krankheitsbildern sie bei ihrer Tätigkeit rechnen müssen.

## Abstract (en)

In Germany, physicians qualify for emergency medicine by combining a specialty medical training—e.g. internal medicine—with advanced training in emergency medicine according to the statutes of the State Chambers of Physicians largely based upon the Guideline Regulations on Specialty Training of the German Medical Association. Internal medicine and their associated subspecialities represent an important column of emergency medicine. For the internal medicine aspects of emergency medicine, this curriculum presents an overview of knowledge, skills (competence levels I–III) as well as behaviours and attitudes allowing for the best treatment of patients. These include general aspects (structure and process quality, primary diagnostics and therapy as well as indication for subsequent treatment; resuscitation room management; diagnostics and monitoring; general therapeutic measures; hygiene measures; and pharmacotherapy) and also specific aspects concerning angiology, endocrinology, diabetology and metabolism, gastroenterology, geriatric medicine, hematology and oncology, infectiology, cardiology, nephrology, palliative care, pneumology, rheumatology and toxicology. Publications focussing on contents of advanced training are quoted in order to support this concept. The curriculum has primarily been written for internists for their advanced emergency training, but it may generally show practising emergency physicians the broad spectrum of internal medicine diseases or comorbidities presented by patients attending the emergency department.

## Vorwort

Der Begriff „Notfallmedizin“ wird in Deutschland bis heute häufig mit der Tätigkeit im Rettungsdienst assoziiert. Jedoch ist die Notfallmedizin in Deutschland heutzutage weit mehr und umfasst neben der prähospitalen Notfallmedizin – dem Rettungsdienst oder besser der Rettungsmedizin – die sektoren- und gebietsübergreifende klinische Akut- und Notfallmedizin in Notfallzentren, Notaufnahmen und Notfallkliniken.

Die Bedeutung der klinischen Akut- und Notfallmedizin im Gesundheitssystem zeigt sich bei der wichtigen Weiterentwicklung der Strukturen in Anlehnung an den Beschluss des gemeinsamen Bundesausschusses (G-BA) zu einem gestuften System von Notfallstrukturen [1]. Der in der Notaufnahme tätige Arzt muss ein breites Spektrum an fächerübergreifendem theoretischem Wissen (Methoden- bzw. kognitive Kompetenzen) und praktischen Fähigkeiten (Handlungskompetenzen) beherrschen.

In einigen europäischen Ländern existiert ein „Facharzt für Unfall- und Notfallmedizin“, in anderen ein „Facharzt für Notfallmedizin“. Die europäischen notfallmedizinischen Gesellschaften haben dafür ein Curriculum erstellt, in dem stichwortartig das breite Spektrum der Notfallmedizin symptomen- und befundorientiert dargestellt ist; eine deutsche Curriculumversion hat die Deutsche Gesellschaft Interdisziplinäre Notfall- und Akutmedizin e. V. (DGINA) ausgearbeitet [2]. In Deutschland basiert dagegen die Weiterbildung zur Tätigkeit in der Notfallmedizin auf einer Facharztqualifikation – z. B. Innere Medizin – ergänzt durch die Zusatz-Weiterbildung (Z-WB) „Klinische Akut- und Notfallmedizin“ (erwerbbar nach der Facharztweiterbildung) und die am prähospitalen Rettungsdienst orientierte Z‑WB „Notfallmedizin“ (erwerbbar bereits während der Facharzt-Weiterbildung; siehe 1.3.1. und 1.3.2.). Die erforderlichen Qualifikationen sind in den beiden Z‑WB der Bundesärztekammer (BÄK) stichwortartig aufgeführt.

Eine zentrale Säule in der gebietsübergreifenden Versorgung stellt das Gebiet der Inneren Medizin mit seinen Schwerpunkten dar. In großen zentralen Notaufnahmen oder Notfallzentren fällt mindestens die Hälfte aller Patienten entsprechend ihrer Diagnosen in den internistisch-konservativen Bereich [3–5]. Die Bedeutung der Inneren Medizin in der klinischen Akut- und Notfallmedizin [6] wird aufgrund der demografischen Entwicklung mit einer zunehmenden Zahl betagter Notfallpatienten an Bedeutung weiter zunehmen [3, 7]. Viele Akut- und Notfallpatienten weisen in der Regel mehrere prognoserelevante internistische Begleiterkrankungen auf, die im Rahmen der Behandlung ebenfalls berücksichtigt werden müssen.

Die BÄK wird dem Stellenwert der Inneren Medizin in der klinischen Akut- und Notfallmedizin insofern gerecht, als dass die (Muster‑)Weiterbildungsordnung ([M-]WBO) zum Facharzt für Innere Medizin bzw. für Innere Medizin und Schwerpunkt einen gut fundierten, ausführlichen notfall- und intensivmedizinischen Weiterbildungsblock vorsieht, mit einer verpflichtenden 6‑monatigen Tätigkeit unter Anleitung in der klinischen Akut- und Notfallmedizin und einer 6‑monatigen Tätigkeit unter Anleitung in der Intensivmedizin.

Allerdings kann der Weiterbildungsblock im Rahmen der internistischen Weiterbildung nicht das gesamte Spektrum der klinischen Akut- und Notfallmedizin abbilden, sodass der Internist, der zukünftig im Bereich der Notfallmedizin tätig sein möchte, im Anschluss an die Facharztqualifikation die bereits genannte Z‑WB „Klinische Akut- und Notfallmedizin“ und ggf. auch schon während seiner Facharztweiterbildung die Z-WB „Notfallmedizin“ zu absolvieren hat (siehe 1.3.1. und 1.3.2).

Das vorliegende Curriculum „Klinische Akut- und Notfallmedizin – Schwerpunkt Innere Medizin“ zu Ausbildungsinhalten der Inneren Medizin in der Notaufnahme der Deutschen Gesellschaft für Internistische Intensivmedizin und Notfallmedizin (DGIIN), der Deutschen Gesellschaft für Innere Medizin (DGIM) samt deren Schwerpunktgesellschaften sowie des Berufsverbands Deutscher Internistinnen und Internisten (BDI) unter Einbeziehung der Deutschen Gesellschaft für Palliativmedizin (DGP) dient einerseits dazu, Empfehlungen zu Ausbildungsinhalten der Inneren Medizin in der Notaufnahme zu geben. Insbesondere dient es aber dazu, die internistischen Inhalte der Zusatz-Weiterbildung „Klinische Akut- und Notfallmedizin“ (siehe 1.3.2.) „mit Leben zu füllen“, die relevanten internistischen akut- und notfallmedizinischen Themen zu benennen, die Lerninhalte zu deklarieren und letztendlich einen umfassenden Überblick über die internistischen Inhalte der Akut- und Notfallmedizin zu geben. Auch die Weiterbildungsinhalte der Z‑WB „Notfallmedizin“ (siehe unter 1.3.1.) werden im Curriculum ausgewiesen und benannt. Dieses Curriculum kann zudem als Leitfaden für den Erwerb der erforderlichen Kenntnisse genutzt werden und es definiert und kategorisiert die notwendigen praktischen Fähigkeiten für eine bestmögliche Versorgung der akut- und notfallmedizinischen Patienten aus internistischer Sicht.

Das Curriculum repräsentiert umfassend die Position und das Verständnis der DGIIN, der DGIM samt Schwerpunktgesellschaften sowie des BDI, welche Expertise, Kenntnisse, Fertigkeiten und auch berufsethische Qualitäten künftige Notfallmediziner in der Inneren Medizin besitzen sollen. Die Gliederung des Curriculums in einen allgemeinen und einen schwerpunktspezifischen Teil ermöglicht es dem Weiterzubildenden, die Notfallmedizin nicht nur als Beseitigung eines akuten „Problems“ zu sehen, sondern als möglichst früher Beginn – bereits in der Notaufnahme – der Behandlung der dem „Problem“ zugrunde liegenden internistischen Erkrankung. Die Autoren erhoffen sich zudem, dass dieses Curriculum auch bei berufspolitischen und standesorganisatorischen Fragestellungen sowie Diskussionen entsprechende Berücksichtigung finden wird.

### Für die Vorstände der DGIIN, der DGIM und deren Schwerpunktgesellschaften sowie des BDI inkl. DGG und DGP (Palliativmedizin).

- Prof. Dr. Matthias Kochanek, Präsident der Deutschen Gesellschaft für Internistische Intensivmedizin und Notfallmedizin e. V. (DGIIN)
- Prof. Dr. Andreas Neubauer, Vorsitzender der Deutschen Gesellschaft für Innere Medizin e. V. (DGIM)
- Prof. Dr. Wulf Ito, Präsident der Deutschen Gesellschaft für Angiologie – Gesellschaft für Gefäßmedizin e. V. (DGA)
- Prof. Dr. Günter Stalla, Past-Präsident der Deutschen Gesellschaft für Endokrinologie e. V. (DGE)
- Prof. Dr. Heiner Wedemeyer, Präsident der Deutschen Gesellschaft für Gastroenterologie, Verdauungs- und Stoffwechselkrankheiten e. V. (DGVS)
- Prof. Dr. med. univ. Markus Gosch, Präsident der Deutschen Gesellschaft für Geriatrie e. V. (DGG)
- Prof. Dr. Hermann Einsele, Geschäftsführender Vorsitzender der Deutschen Gesellschaft für Hämatologie und Medizinische Onkologie e.V. (DGHO)
- Prof. Dr. Bernd Salzberger, Past-Präsident der Deutschen Gesellschaft für Infektiologie e. V. (DGI)
- Prof. Dr. Holger Thiele, Präsident der Deutschen Gesellschaft für Kardiologie – Herz- und Kreislaufforschung e. V. (DGK)
- Prof. Dr. Hermann Pavenstädt, Präsident der Deutschen Gesellschaft für Nephrologie e. V. (DGfN)
- Prof. Dr. Claudia Bausewein, Präsidentin der Deutschen Gesellschaft für Palliativmedizin e. V. (DGP)
- Prof. Dr. Wolfram Windisch, Präsident der Deutschen Gesellschaft für Pneumologie und Beatmungsmedizin e. V. (DGP)
- Prof. Dr. Christof Specker, Präsident der Deutschen Gesellschaft für Rheumatologie e. V. (DGRh)
- Christine Neumann-Grutzeck, Präsidentin des Berufsverbands Deutscher Internistinnen und Internisten e. V. (BDI)

## Inhaltsverzeichnis

1.: Weiterbildung „Klinische Akut- und Notfallmedizin – Schwerpunkt Innere Medizin“
1.1.: Die (Muster‑)Weiterbildungsordnung ([M-]WBO) der Bundesärztekammer
1.2.: Klinische Akut- und Notfallmedizin in der (M-)WBO zum Facharzt für Innere Medizin und zum Facharzt für Innere Medizin und Schwerpunkt
1.3.: Akut- und notfallmedizinische Zusatz-Weiterbildungen (Z‑WB) der Bundesärztekammer (BÄK)
1.3.1.: Zusatz-Weiterbildung (Z‑WB) „Notfallmedizin“
1.3.2.: Zusatz-Weiterbildung (Z‑WB) „Klinische Akut- und Notfallmedizin“
1.4.: Empfehlung zu Weiterbildungsinhalten der Inneren Medizin in der Notaufnahme
2.: Adressaten des Curriculums „Klinische Akut- und Notfallmedizin – Schwerpunkt Innere Medizin“
2.1.: Weiterzubildende
2.2.: Weiterbilder
2.3.: Gremien und Ärztekammern
3.: Erforderliche Qualifikationen
3.1.: Theoretische Kenntnisse – praktische Fähigkeiten – professionelles Verhalten
3.2.: Kompetenzgraduierung – Level I, II und III
4.: Durchführung der Z‑WB für Internisten und Schwerpunktinternisten unter Einbeziehung des Curriculums
4.1.: Weiterbildungsinhalte und Weiterbildungsdauer
4.2.: Aktivitätsnachweise und Einbindung von DGIIN, DGIM und BDI
4.3.: Mindestmengen
5.: Curriculum: Anforderungen an den Weiterzubildenden
6.: Curriculum: Anforderungen an den Weiterbilder und an die Weiterbildungsstätte
7.: Curriculum: Dokumentation der Zusatz-Weiterbildung
8.: Curriculum: Akkreditierung der Weiterbildungsstätte
9.: Zertifizierung des Weiterzubildenden
10.: Vorgesehene Aktualisierung des Curriculums
11.: Ziele des Curriculums: internistische Weiterbildungsinhalte der Akut- und Notfallmedizin umfassend und aktuell präsentieren!
11.1.: Allgemeine Aspekte der Klinischen Akut- und Notfallmedizin – Schwerpunkt Innere Medizin
11.1.1.: Allgemeiner Teil – Struktur- und Prozessqualität (Tab. 1)
11.1.2.: Allgemeiner Teil – Erstdiagnostik, Initialtherapie und Indikationsstellung zur weiterführenden Behandlung (Tab. 2)
11.1.3.: Allgemeiner Teil – Schockraumversorgung (Tab. 3)
11.1.4.: Allgemeiner Teil – Diagnostik und Monitoring (Tab. 4)
11.1.5.: Allgemeiner Teil – generelle Therapieverfahren (Tab. 5)
11.1.6.: Allgemeiner Teil – Hygienemaßnahmen (Tab. 6)
11.1.7.: Allgemeiner Teil – Pharmakotherapie (Tab. 7)
11.2.: Angiologische Aspekte in der Akut- und Notfallmedizin (Tab. 8)
11.3.: Endokrinologische, diabetologische und metabolische Aspekte in der Akut- und Notfallmedizin (Tab. 9)
11.4.: Gastroenterologische Aspekte in der Akut- und Notfallmedizin (Tab. 10)
11.5.: Geriatrische Aspekte in der Akut- und Notfallmedizin (Tab. 11)
11.6.: Hämatoonkologische Aspekte in der Akut- und Notfallmedizin (Tab. 12)
11.7.: Infektiologische Aspekte der Akut- und Notfallmedizin (Tab. 13)
11.8.: Kardiologische Aspekte der Akut- und Notfallmedizin (Tab. 14)
11.9.: Nephrologische Aspekte der Akut- und Notfallmedizin (Tab. 15)
11.10.: Palliativmedizinische Aspekte der Akut- und Notfallmedizin (Tab. 16)
11.11.: Pneumologische Aspekte der Akut- und Notfallmedizin (Tab. 17)
11.12.: Rheumatologische Aspekte der Akut- und Notfallmedizin (Tab. 18)
11.13.: Toxikologische Aspekte der Akut- und Notfallmedizin (Tab. 19)

## 1. Weiterbildung „Klinische Akut- und Notfallmedizin – Schwerpunkt Innere Medizin“

### 1.1. Die (Muster‑)Weiterbildungsordnung ([M-]WBO) der Bundesärztekammer

- Der **Begriff „Weiterbildung“** im engeren Sinne ist ein Terminus der (Muster‑)Weiterbildungsordnung ([M-]WBO) mit Prüfungsabschluss, für die die Bundesärztekammer und die Landesärztekammern verantwortlich zeichnen. Die Präambel der (M-)WBO führt dazu Folgendes an [8]: *„Ärztliche Weiterbildung beinhaltet das Erlernen spezieller ärztlicher Fähigkeiten und Fertigkeiten nach abgeschlossenem Studium der Humanmedizin und nach Erteilung der Erlaubnis zur Ausübung der ärztlichen Tätigkeit. Im Interesse der Patienten werden die in der Ausbildung geprägten ärztlichen Kompetenzen und Haltungen während der Weiterbildung vertieft. Kennzeichnend für die Weiterbildung ist die vertiefende Anwendung ärztlicher Kenntnisse in der Berufsausübung. Die Weiterbildung erfolgt in strukturierter Form, um in Gebieten die Qualifikation als Facharzt, darauf aufbauend eine Spezialisierung in Schwerpunkten oder in einer Zusatz-Weiterbildung zu erhalten … Die Weiterbildung wird in angemessen vergüteter hauptberuflicher Ausübung der ärztlichen Tätigkeit an zugelassenen Weiterbildungsstätten durchgeführt. Sie erfolgt unter Anleitung befugter Ärzte in praktischer Tätigkeit und theoretischer Unterweisung sowie teilweise durch die erfolgreiche Teilnahme an anerkannten Kursen … Die Weiterbildungsbezeichnung ist der Nachweis für erworbene Kompetenz. Sie dient der Qualitätssicherung der Patientenversorgung und der Bürgerorientierung.“*
- Die **Novellierung der (Muster‑)Weiterbildungsordnung** ([M-]WBO) der Bundesärztekammer ist im November 2018 mit der Publikation [8] erfolgreich zum Abschluss gebracht worden. Die (M-)WBO beinhaltet neben der Gebiets‑, Facharzt- und Schwerpunkt-Weiterbildung auch die darauf aufbauenden Zusatz-Weiterbildungen (Z‑WB), die gebietsübergreifend erworben werden können. Derzeit erarbeitet die Bundesärztekammer für alle Weiterbildungsordnungen inkl. der Zusatz-Weiterbildungen den sog. fachlich empfohlenen Weiterbildungsplan (FEWP) zur Konkretisierung der Weiterbildungsinhalte. Die FEWPs werden nach Fertigstellung in die Weiterbildungsordnungen als Ergänzung der kognitiven und Methodenkompetenz- und Handlungskompetenzspalten integriert werden.
- Die **Fachgesellschaften** haben die Möglichkeit, bei einer Aktualisierung der (M-)WBO („Novellierung“) durch die BÄK beratend die Weiterbildungsinhalte mit zu definieren: In Vorbereitung einer Novellierung der (M-)WBO durch die BÄK werden die Fachgesellschaften von der BÄK aufgefordert, Vorschläge hinsichtlich der Weiterbildungsinhalte zu unterbreiten. Nach Fertigstellung der Novellierung durch die BÄK legt diese die (M-)WBO-Novellierung dem Ärztetag zur Beschlussfassung vor. Nach Zustimmung des Ärztetags leitet die BÄK die novellierte (M-)WBO als „Muster“-Vorschlag den Landesärztekammern zur Umsetzung zu, wobei diese die Möglichkeit haben, durch Modifikationen die (M-)WBO der BÄK in die definitive Weiterbildungsordnung für ihren jeweiligen Ärztekammerbereich (WBO) umzugestalten. In dieser Phase besteht wiederum für Mitglieder der Fachgesellschaften, die in Gremien der Landesärztekammer mitarbeiten, die Möglichkeit – dieses Mal auf der Ebene der jeweiligen Landesärztekammer – beratend Modifikationen der Weiterbildungsinhalte vorzuschlagen.

### 1.2. Klinische Akut- und Notfallmedizin in der (M-)WBO zum Facharzt für Innere Medizin bzw. zum Facharzt für Innere Medizin und Schwerpunkt

- In der **(M-)WBO zum Facharzt für Innere Medizin** bzw. zum **Facharzt für Innere Medizin und Schwerpunkt** [8] ist festgelegt, dass jeder zukünftige Facharzt im Rahmen seiner 60- bzw. 72-monatigen Weiterbildungszeit unter Befugnis im Gebiet Innere Medizin 6 Monate in der Notfallaufnahme und 6 Monate in der Intensivmedizin ableisten muss.

Die notfallmedizinische Weiterbildung im Rahmen der Weiterbildung zum Facharzt für Innere Medizin und zum Facharzt für Innere Medizin und Schwerpunkt ist eine gute Basis für die Mitarbeit im Bereich der Akut- und Notfallmedizin zur Behandlung internistischer Patienten und zur Mitbehandlung internistischer Komorbiditäten bei nichtinternistischen Patienten.

- Notfall- und intensivmedizinische Maßnahmen im Gebiet Innere Medizin:
  Die gemeinsamen Inhalte der Facharztweiterbildungen im Gebiet Innere Medizin sind für die Notfall- und Intensivmedizin im Folgenden zusammengefasst.
  - Kognitive und Methodenkompetenz (Kenntnisse):
    - Differenzierte Beatmungstechniken.
  - Handlungskompetenz (Erfahrungen und Fertigkeiten):
    - Stufendiagnostik und Therapie bei akut einsetzenden Leitsymptomen, z. B. Dyspnoe, Thoraxschmerz, Bauchschmerz, passagere und persistierende Bewusstseinsstörungen, Fieber, Erbrechen, Durchfall;
    - Diagnostik und Therapie akuter und vital bedrohlicher Erkrankungen und Zustände insbesondere respiratorische Insuffizienz, Schock, kardiale Insuffizienz, akutes Nierenversagen, sonstige Ein- und Mehrorganversagen, Koma und Delir, akute Enzephalopathie, Sepsis und Intoxikationen;
    - Kardiopulmonale Reanimation;
    - intensivmedizinische Behandlung von Patienten mit Funktionsstörungen von mindestens 2 vitalen Organsystemen;
    - Analgosedierung von intensivmedizinischen Patienten;
    - atemunterstützende Maßnahmen bei intubierten und nichtintubierten Patienten einschließlich Beatmungsentwöhnung bei langzeitbeatmeten Patienten;
    - Therapie von Stoffwechselentgleisungen;
    - Notfallsonographie;
    - Notfallbronchoskopie;
    - passagere Schrittmacheranlage;
    - Punktions- und Katheterisierungstechniken (insbesondere zentralvenöse Zugänge und arterielle Gefäßzugänge).
- **Facharzt für Innere Medizin und Kardiologie:** Neben den gemeinsamen Inhalten zur Notfall- und Intensivmedizin für alle internistischen WBOs (siehe oben) enthält die WBO für den Facharzt für Innere Medizin und Kardiologie noch zusätzlich einen Weiterbildungsblock für kardiovaskuläre Notfall- und Intensivmedizin.
  - Kognitive und Methodenkompetenz (Kenntnisse):
    - Herzunterstützende Verfahren.
  - Handlungskompetenz (Erfahrungen und Fertigkeiten):
    - Behandlung des Herz-Kreislauf-Versagens in der Akutphase;
    - Management der Postreanimationsphase;
    - Akutbehandlung von Patienten mit akuten und bedrohlichen Herz-Kreislauferkrankungen, insbesondere akutes Thoraxschmerzsyndrom, auch in Notaufnahme und Chest Pain Unit, Intermediate Care und internistischer Intensivmedizin;
    - invasives hämodynamisches Monitoring;
    - Organ-unterstützende Verfahren, z. B. nichtinvasive und invasive Beatmung, intraaortale Ballongegenpulsation, perkutane Herz-Lungen-Maschine, extrakorporale Membranoxygenierung, perkutane Herzunterstützungssysteme;
    - Akutbehandlung des Herz-Kreislauf-Schocks, insbesondere des kardiogenen Schocks;
    - Mitbehandlung des Multiorgan-Dysfunktions-Syndroms.
- **Facharzt für Innere Medizin und Infektiologie:** Neben den gemeinsamen Inhalten zur Notfall- und Intensivmedizin für alle internistischen WBOs (siehe oben) enthält die WBO für den Facharzt für Innere Medizin und Infektiologie noch zusätzlich einen Weiterbildungsblock für Infektiologische Notfälle.
  - Kognitive und Methodenkompetenz (Kenntnisse):
    - Akute lebensbedrohliche Infektionen und infektiologische Notfälle.
  - Handlungskompetenz (Erfahrungen und Fertigkeiten):
    - Beurteilung des Schweregrads von Infektionen;
    - Erkennung und Behandlung einschließlich Erstversorgung von Infektionen mit hoher Kontagiosität;
    - interdisziplinäre Beratung und Akutbehandlung bei lebensbedrohlichen Infektionen;
    - Erkennung und Therapie der Sepsis und des septischen Schocks, auch in interdisziplinärer Zusammenarbeit.

### 1.3. Akut- und notfallmedizinische Zusatz-Weiterbildungen (Z-WB) der Bundesärztekammer (BÄK)

Für die Tätigkeiten in der prähospitalen und klinischen Akut- und Notfallmedizin hat die Bundesärztekammer 2 Zusatz-Weiterbildungen geschaffen, die Z‑WB „Notfallmedizin“ und die Z-WB „Klinische Akut- und Notfallmedizin“. Die Z‑WB „Notfallmedizin“ kann bereits während einer Facharztausbildung absolviert werden und fokussiert mit den 50 Notarzteinsätzen vor allem auf die *„Erkennung drohender oder eingetretener Notfallsituationen und die Behandlung von Notfällen sowie die Wiederherstellung und Aufrechterhaltung akut bedrohter Vitalfunktonen.“* Dagegen vermittelt die Z‑WB „Klinische Akut- und Notfallmedizin“ einem fertigen Facharzt die zusätzlichen interdisziplinären Kompetenzen zur *„Erstdiagnostik und Initialtherapie von Notfall- und Akutpatienten im Krankenhaus sowie zur Indikationsstellung und Koordination der weiterführenden fachspezifischen Behandlung in interdisziplinärer Zusammenarbeit.“*

#### 1.3.1. Zusatz-Weiterbildung (Z-WB) „Notfallmedizin“ [8]

- **Definition:** „Die Zusatz-Weiterbildung Notfallmedizin umfasst die Erkennung drohender oder eingetretener Notfallsituationen und die Behandlung von Notfällen sowie die Wiederherstellung und Aufrechterhaltung akut bedrohter Vitalfunktionen.“
- Die **Mindestanforderungen gemäß § 11 MWBO** für diese Z‑WB sind 24 Monate Weiterbildung in einem Gebiet der unmittelbaren Patientenversorgung im stationären Bereich unter Befugnis an Weiterbildungsstätten, davon 6 Monate in der Intensivmedizin oder Anästhesiologie plus 80 h-Kurs-Weiterbildung gemäß § 4 Abs. 8 in allgemeiner und spezieller Notfallbehandlung plus anschließend 50 Notarzteinsätze im öffentlichen Rettungsdienst (Notarzteinsatzfahrzeug oder Rettungshubschrauber) unter Anleitung eines verantwortlichen Notarztes, davon können bis zu 25 Einsätze im Rahmen eines standardisierten Simulationskurses erfolgen.
- Die **Weiterbildungsinhalte** der Z‑WB gliedern sich in
  - organisatorische, einsatztaktische Grundlagen;
  - die Untersuchung des Notfallpatienten;
  - **Leitsymptome:** *Handlungskompetenz* (Erfahrungen und Fertigkeiten) in der Einleitung einer symptomorientierten Erstbehandlung bei → Bewusstseinsstörungen/neurologischen Defiziten, → akuter Atemnot, → Brustschmerz, → Blutungen, → Schock, → Herzrhythmusstörungen, → akutem Abdomen/Bauchschmerzen, → psychischen Störungen, → Fieber;
  - **diagnostische Maßnahmen: ***Handlungskompetenz* (Erfahrungen und Fertigkeiten) → Durchführung und Befunderstellung des Elektrokardiogramms im Notfall, → Applikation und Bewertung des Basismonitorings einschließlich Besonderheiten des kindgerechten Monitorings beim Transport, Messung und Bewertung der Kapnometrie und Kapnographie;
  - **therapeutische Maßnahmen: ***kognitive und Methodenkompetenz* (Kenntnisse) u. a. in Grundlagen der transkutanen Schrittmachertherapie; *Handlungskompetenz* (Erfahrungen und Fertigkeiten) u. a. bei der Durchführung von Defibrillation oder Kardioversion, auch als Simulation.

#### 1.3.2. Zusatz-Weiterbildung (Z-WB) „Klinische Akut- und Notfallmedizin“ [8, 9]

- **Definition:** „Die Zusatz-Weiterbildung Klinische Akut- und Notfallmedizin umfasst in Ergänzung zu einer Facharztkompetenz die Erstdiagnostik und Initialtherapie von Notfall- und Akutpatienten im Krankenhaus sowie die Indikationsstellung und Koordination der weiterführenden fachspezifischen Behandlung in interdisziplinärer Zusammenarbeit.“ [8].
- Die **Mindestanforderungen gemäß § 11 MWBO** für diese Z‑WB sind die Facharztanerkennung in einem Gebiet der unmittelbaren Patientenversorgung plus 6 Monate Intensivmedizin, die auch während der Facharztweiterbildung abgeleistet werden können, plus 80 h-Kurs-Weiterbildung gemäß § 4 Abs. 8 in allgemeiner und spezieller Notfallbehandlung plus 24 Monate klinische Akut- und Notfallmedizin in einer interdisziplinären Notfallaufnahme unter Befugnis an Weiterbildungsstätten [8].
- Die **Weiterbildungsinhalte** der Z‑WB gliedern sich in
  - **übergreifende Inhalte**;
  - **symptomorientierte Erstdiagnostik und Initialtherapie**;
  - **alters- und geschlechtsspezifische Notfälle** (Kindes- und Jugendalter, Schwangerschaft, geriatrische Patienten);
  - **notfallmedizinische Kernverfahren**;
  - **organbezogene und spezifische Notfallsituationen.**
    Die Weiterbildungsinhalte zu organbezogenen und spezifischen Notfallsituationen beinhalten keine *Handlungskompetenz* (Erfahrungen und Fertigkeiten), wohl aber *kognitive und Methodenkompetenz* (Kenntnisse) in Form der Differenzialdiagnostik und Therapieoptionen organbezogener Notfälle:
    → kardiovaskuläre Notfälle, → hämatologische und onkologische Notfälle; → immunologische Notfälle, → Infektionskrankheiten und Sepsis, → endokrine und metabolische Notfälle, → Flüssigkeits- und Elektrolytstörungen; → gastrointestinale und hepatologische Notfälle, → respiratorische Notfälle; → nephrologische und urologische Notfälle, → dermatologische Notfälle; → Notfälle im Hals‑, Nasen‑, Ohren‑, Mund- und Nackenbereich; → gynäkologische Notfälle, → muskuloskelettale Notfälle, → neurologische Notfälle, → neurochirurgische Notfälle, → ophthalmologische Notfälle; → psychiatrische Notfälle und Verhaltensstörungen, → Trauma (stumpf/penetrierend), → akute Notfälle durch Umwelteinflüsse, thermische, hyper- und hypobare Exposition und elektrischen Strom.

### 1.4. Empfehlung zu Weiterbildungsinhalten der Inneren Medizin in der Notaufnahme

- Das vorliegende Curriculum beschreibt die internistischen Weiterbildungsinhalte der präklinischen und klinischen Akut- und Notfallmedizin, die für die Betreuung internistischer akut- und notfallmedizinischer Patienten zu erwerben sind, aber auch für die Mitbetreuung nichtinternistischer Akut- und Notfallpatienten – Patienten mit internistischen Komorbiditäten und bei internistischen Notfällen – beherrscht werden sollen. Das Curriculum wurde gemeinsam von Mitgliedern der Deutschen Gesellschaft für Internistische Intensiv- und Notfallmedizin (DGIIN), der Deutschen Gesellschaft für Innere Medizin (DGIM) samt deren Schwerpunktgesellschaften sowie des Berufsverbands Deutscher Internistinnen und Internisten (BDI) erstellt. Das Curriculum soll dem Facharzt für Innere Medizin bei der Absolvierung der Zusatz-Weiterbildung „Klinische Akut- und Notfallmedizin“ die Weiterbildungsinhalte aus dem Bereich der Inneren Medizin aufzeigen. Das Curriculum inkludiert auch die internistischen Weiterbildungsinhalte der auf die präklinische Notfallmedizin fokussierten Zusatz-Weiterbildung „Notfallmedizin“. Selbstredend umfasst dieses Curriculum nur die internistischen Weiterbildungsinhalte und nicht das gesamte restliche notfallmedizinische Spektrum.
- Die aufgeführten Weiterbildungsinhalte berücksichtigen auch die entsprechenden Weiterbildungsinhalte des **European Core Curriculum for Emergency Medicine** der European Society for Emergency Medicine und der Section for Emergency Medicine der European Union of Medical Specialists (UEMS; [2] sowie internationale Weiterbildungskonzepte [10]).

## 2. Adressaten des Curriculums „Klinische Akut- und Notfallmedizin – Schwerpunkt Innere Medizin“

### 2.1. Weiterzubildende

Ziel dieses Curriculums ist es, aus Sicht der DGIIN, der DGIM samt deren Schwerpunktgesellschaften, des BDI und unter Einbeziehung der Palliativmedizin aufzuzeigen, welche Kompetenzen auf dem Gebiet der internistischen Akut- und Notfallmedizin heutzutage benötigt werden. Dem in den Z‑WB „Klinische Akut- und Notfallmedizin“ oder „Notfallmedizin“ Weiterzubildenden soll das Curriculum die Möglichkeit geben, seine Zusatzweiterbildungszeit hinsichtlich der internistischen Weiterbildungsinhalte so effizient wie möglich zu strukturieren, sich gut auf die Prüfung vorzubereiten und das Erlernte anschließend im Sinne eines „berufslebenslangen“ Qualifizierens zu bewahren und auszubauen. Wie bereits im Vorwort ausgeführt erlaubt die Gliederung des Curriculums in einen allgemeinen und einen schwerpunktspezifischen Teil es dem Weiterzubildenden, die Notfallmedizin nicht nur als Beseitigung eines akuten „Problems“ zu sehen, sondern als möglichst früher Beginn der Behandlung der dem „Problem“ zugrunde liegenden internistischen Erkrankung bereits in der Notaufnahme.

### 2.2. Weiterbilder

Das Curriculum möchte aber nicht nur die Weiterzubildenden, sondern auch die Weiterbilder in den Z‑WBs „Klinische Akut- und Notfallmedizin“ sowie „Notfallmedizin“ erreichen und aufzeigen, welche internistischen Inhalte und Fertigkeiten nach Ansicht von DGIIN, DGIM und BDI in den Z‑WB vermittelt werden sollen, um alle Aspekte der Inneren Medizin in der Akut- und Notfallmedizin für die Patienten bestmöglich einsetzen zu können.

### 2.3. Gremien und Ärztekammern

Und schließlich soll das Curriculum den für die Zusatz-Weiterbildungen verantwortlichen Gremien der Ärztekammern das breite Spektrum der internistischen Akut- und Notfallmedizin aufzeigen, das nach Ansicht von DGIIN, DGIM und BDI in die Z‑WBs „Klinische Akut- und Notfallmedizin“ bzw. „Notfallmedizin“ einfließen soll.

## 3. Erforderliche Qualifikationen

### 3.1. Theoretische Kenntnisse – praktische Fähigkeiten – professionelles Verhalten

Die Qualifikation der Weiterzubildenden wird durch „theoretische Kenntnisse“ („knowledge“; TK), „praktische Fähigkeiten“ („skills“; PF) und beruflich-professionelles Verhalten („behaviours and attitudes“; BV) erworben und aufrechterhalten. Diese Klassifizierung „knowledge“, „skills“ und „behaviours and attitudes“ ist international akzeptiert [11, 12] und auch auf nationaler Ebene – z. B. in Deutschland [13] – im Einsatz. Die Bundesärztekammer [8] verwendet in der (M-)WBO die Begriffe „*Kognitive und Methodenkompetenz – Kenntnisse*“ und „*Handlungskompetenz – Erfahrungen und Fertigkeiten“*, die unschwer mit den in diesem Curriculum verwandten Begriffen „theoretische Kenntnisse“ und „praktische Fähigkeiten“ gleichgesetzt werden können. Dagegen ist der für den Berufsalltag wichtige Weiterbildungsinhalt „beruflich-professionelles Verhalten“ in der (M-)WBO nicht explizit abgebildet.

Die TK-PF-BV-Einteilung bildet die Basis der Klassifikation dieses Curriculums. Dieses standardisierte Vorgehen erleichtert den Vergleich der Zusatzweiterbildungsinhalte der Akut- und Notfallmedizin mit den Weiterbildungsinhalten der internistischen Intensivmedizin [14] und der internistischen Schwerpunkte, z. B. der Kardiologie [13, 15, 16]:

- Die **„theoretischen Kenntnisse“** (TK, „*knowledge*“) definieren sich aus den stichwortartig aufgeführten Themenschwerpunkten bzw. den kognitiven und Methodenkompetenzen der internistischen Weiterbildungsinhalte der Z‑WB „Klinische Akut- und Notfallmedizin“ und der Z‑WB „Notfallmedizin“ (siehe Tab. 1–19). Die theoretischen Wissensanteile sind das essenzielle Fundament der Kompetenzentwicklung.
- Die **„praktischen Fähigkeiten“** (PF, „*skills*“) bzw. Handlungskompetenzen beschreiben die effektive Anwendung von theoretischem Wissen zur Lösung von Problemen, zu klinischen Entscheidungsfindungen und – aufbauend auf Erfahrung und Training – zur Durchführung von Prozeduren. Simulationstraining (siehe Kurse der DGIIN) im Team stellt eine sinnvolle Ergänzung in der kompetenzorientierten Zusatz-Weiterbildung dar und führt zu einer vertrauensvollen interdisziplinären und interprofessionellen Zusammenarbeit. Die „*skills*“ sollten sich allerdings nicht allein auf die fachpraktischen, die sog. „*hard skills*“, beschränken, sondern persönliche, soziale und methodische Kompetenzen, sog. „s*oft skills*“, mit einschließen.
- Das **„beruflich-professionelle Verhalten“** (BV, „behaviours and attitudes“) muss der Akut- und Notfallmediziner lernen und beruflich „leben“ im Umgang mit Patienten und Angehörigen, allen beteiligten Berufsgruppen – sowohl interdisziplinär als auch interprofessionell – und anderen Akteuren im Gesundheitswesen.

### 3.2. Kompetenzgraduierung – Level I, II und III

Das sehr aufgefächerte gesamte Methodenspektrum der internistischen Inhalte in der Akut- und Notfallmedizin muss zwar von jedem Weiterzubildenden „gewusst“ werden, nicht jeder Akut- und Notfallmediziner kann aber alle Spezialkenntnisse bzw. Techniken der integrierten internistischen Schwerpunktfächer – wie z. B. die Behandlung eines dekompensierten Vitiums oder die extrakorporale Reanimation (eCPR) – selbständig durchführen. Insofern muss hinsichtlich der „Eindringtiefe“ im Beherrschen praktischer Fähigkeiten zwangsläufig eine Selektion vorgenommen werden zwischen dem selbständigen bzw. nichtselbständigen Beherrschen der breiten Palette notfallmedizinischer Methoden und Techniken und dem Wissen um erweiterte Methoden und Techniken im Speziellen. Letztere können einerseits anhand weiterführender, auf dem Curriculum „Klinische Akut- und Notfallmedizin – Schwerpunkt Innere Medizin“ aufbauender Curricula der internistischen Schwerpunktfächer erworben werden, z. B. dem Curriculum „Kardiovaskuläre Intensiv- und Notfallmedizin“ der Deutschen Gesellschaft für Kardiologie – Herz- und Kreislaufforschung e. V. (DGK; [15]); andererseits dienen dazu die Empfehlungen fächerübergreifender Konsensuspapiere unter Einbeziehung der Deutschen Interdisziplinären Vereinigung für Intensiv- und Notfallmedizin (DIVI), z. B. die Empfehlungen zur extrakorporalen Reanimation (eCPR; [17]).

Hinsichtlich der Kompetenzgraduierung orientiert sich das vorliegende Curriculum ebenfalls an der von der Europäischen Gesellschaft für Kardiologie (ESC) beschriebenen Graduierung [11] mit den Kompetenzlevels I–III:

- Kompetenzlevel I für praktische Fähigkeiten („skills“):
  - Erfahrung bei der Auswahl der geeigneten diagnostischen oder therapeutischen Maßnahmen und der Interpretation der erhaltenen Ergebnisse;
  - Erfahrung bei der Suche nach einer geeigneten Behandlung, zu der der Patient überwiesen werden soll;
  - Level I erfordert zwar umfassende theoretische Kenntnisse der Methoden, jedoch keine Beherrschung der Techniken.
- Kompetenzlevel II für praktische Fähigkeiten („skills“):
  - Level II geht über Level I hinaus: Zusätzlich zur Level-I-Kompetenz soll der Weiterzubildende sich praktische Erfahrungen aneignen und bewahren, aber nur als nichtselbständiger und nicht als eigenverantwortlicher Untersucher (der Weiterzubildende assistiert oder führt eine spezielle Technik oder Prozedur unter Anleitung durch).
- Kompetenzlevel III für praktische Fähigkeiten („skills“):
  - Level III geht über Level I und Level II hinaus. Der Weiterzubildende soll lernen und die Kompetenz bewahren, eigenständig für ein diagnostisches oder therapeutisches Verfahren die Indikation zu erkennen, die Technik oder die Prozedur durchzuführen, die Daten zu interpretieren und Komplikationen zu beherrschen.

In den Tab. 1–19 sind die Level-Angaben mit einem * gekennzeichnet.

## 4. Durchführung der Z‑WBs für Internisten und Schwerpunktinternisten unter Einbeziehung des Curriculums

### 4.1. Weiterbildungsinhalte und Weiterbildungsdauer

Weiterbildungsinhalte und -dauer für die Z‑WB „Klinische Akut- und Notfallmedizin“ und die Z‑WB „Notfallmedizin“ sind unter 1.3. beschrieben. Die von DGIIN, DGIM und BDI im Einklang mit den Z‑WB empfohlenen internistischen Weiterbildungsinhalte finden sich im Curriculum.

### 4.2. Aktivitätsnachweise und Einbindung von DGIIN, DGIM und BDI

- Obligat ist die Dokumentation der erworbenen Weiterbildungsinhalte für die Z‑WB „Klinische Akut- und Notfallmedizin“ und die Z‑WB „Notfallmedizin“ entsprechend der WBO der zuständigen Ärztekammer.
- Der Besuch nationaler und internationaler Fachtagungen/Kongresse mit internistisch-akutmedizinischen und internistisch-notfallmedizinischen Programmteilen ist empfehlenswert. Auf nationaler Ebene bieten sich dabei die Kongresse und Kurse der DGIIN/ÖGIAIN, der DIVI und DGINA an.
- Die Weiter- und Fortbildungsveranstaltungen und -kurse der DGIIN gehen gezielt auf die im Curriculum genannten Zusatzweiterbildungsinhalte ein.

### 4.3. Mindestmengen

- Beim Erlernen von Techniken spielen das persönliche Handanlegen und die praktische Erfahrung eine große Rolle. Zwar ist die Zahl der durchgeführten Untersuchungen keine Garantie dafür, dass die Prozedur vom Weiterzubildenden kompetent beherrscht wird; dennoch vermittelt die Durchführung einer bestimmten Anzahl bei ausgewählten Prozeduren eine gewisse Sicherheit, die in Bezug auf den Patienten gefordert werden muss.
- Die notfallmedizinischen Weiterbildungsinhalte für das Fachgebiet Innere Medizin enthalten keine entsprechenden Richtzahlen, ebenso wenig wie die Z‑WB „Klinische Akut- und Notfallmedizin“. In der Z‑WB „Notfallmedizin“ werden folgende Richtzahlen genannt: Indikationsstellung und Durchführung von Repositionen bei Frakturen und Luxationen: 5 sowie Sicherung der Atemwege durch endotracheale Intubation einschließlich Videolaryngoskopie: 50.

## 5. Curriculum: Anforderungen an den Weiterzubildenden

- Jeder Facharzt, wie z. B der Facharzt für „Innere Medizin“ bzw. „Innere Medizin und Schwerpunkt“, der die Z‑WB „Klinische Akut- und Notfallmedizin“ anstrebt und anschließend als Akut- und Notfallmediziner arbeiten möchte, soll sich im Rahmen seiner Z‑WB-Zeit die von den Ärztekammern in der WBO geforderte Kognitive und Methodenkompetenz (Kenntnisse) und Handlungskompetenz (Erfahrungen und Fertigkeiten) aneignen. Das vorliegende Curriculum versucht, die von den Ärztekammern vorgeschriebenen internistischen Weiterbildungsinhalte entsprechend den Vorstellungen von DGIIN, DGIM und BDI anhand der geforderten theoretischen Kenntnisse, der praktischen Fähigkeiten und des beruflich professionellen Verhaltens „mit Leben zu füllen“.
- Hilfreich – sowohl für die Teamarbeit als auch für die Patientensicherheit – sind auch das Lernen mit digitalen Medien [18] und Ausbildungskonzepte, die eine Simulation von Fällen und Szenarien aller Art anbieten [19–21].
- Die zugehörige **Qualifikationen** sind die Z‑WB-Prüfungen „Klinische Akut- und Notfallmedizin“ und „Notfallmedizin“ durch die jeweilige Landesärztekammer.

## 6. Curriculum: Anforderungen an den Weiterbilder und an die Weiterbildungsstätte

- Der **Weiterbilder** für die Inhalte des Curriculums „Klinische Akut- und Notfallmedizin – Schwerpunkt Innere Medizin“ arbeitet in Vollzeit- bzw. in überwiegender Tätigkeit in einer/einem interdisziplinären Notaufnahme/Klinik für Akut- und Notfallmedizin/Zentrum für Notfallmedizin, in der/dem sich der fachärztliche Weiterbilder „Innere Medizin“ besonders für die internistischen Akut- und Notfallpatienten verantwortlich zeichnet.
- Die **Weiterbildungsstätte** sollte – unterstützt von der Klinikleitung [22] – mit einer adäquaten Prozess- und Strukturqualität ausgestattet sein, um den Weiterzubildenden die in Abschn. 11 aufgeführten Weiterbildungsinhalte zu ermöglichen. Sehr zu begrüßen ist die staatlich finanzierte notfallmedizinische Weiterbildung in manchen Ländern wie Schweden [23].
- Anzustreben ist hinsichtlich des Erstellens eines Qualitätskriterienkatalogs für Weiterbilder und Weiterbildungsstätten eine Kooperation der DGIIN mit den Landesärztekammern, wie dies im Fall der Weiterbildung zum Facharzt für Innere Medizin und Kardiologie bereits realisiert ist.

## 7. Curriculum: Dokumentation der Weiterbildung

- Das Curriculum gibt dem in klinischer Akut- und Notfallmedizin Weiterzubildenden die Möglichkeit, sich systematisch die internistischen Weiterbildungsinhalte zu erarbeiten und sie mit den internistischen Inhalten der Z‑WB „Klinische Akut- und Notfallmedizin“ und der Z‑WB „Notfallmedizin“ abzugleichen. Die erlernten und geübten Weiterbildungsinhalte sollen entsprechend der Z‑WB-Version der zuständigen Ärztekammer anhand des (elektronischen) Logbuchs und der Zeugnisse der Weiterbilder dokumentiert werden.
- DGIIN, DGIM und BDI unterstützen die Weiterzubildenden beratend und anhand des Fortbildungs- und Kursangebots bei der strukturierten Z‑WB entsprechend dem Curriculum. Eine zusätzliche Zertifizierung der in diesem Curriculum vorgeschlagenen Weiterbildungsinhalte ist nicht vorgesehen.
- Der Weiterbilder wird gebeten, im Weiterbildungszeugnis nicht nur die Erfüllung der in der Z‑WB geforderten Weiterbildungsinhalte zu dokumentieren, sondern auch die in diesem Curriculum vorgelegten Weiterbildungsinhalte.

## 8. Curriculum: Akkreditierung der Weiterbildungsstätte

- Hinsichtlich der Akkreditierung der Weiterbildungsstätte gilt das unter Abschn. 6 Gesagte.
- Eine darüber hinaus gehende Akkreditierung durch die DGIIN als Fachgesellschaft ist nicht vorgesehen.

## 9. Zertifizierung des Weiterzubildenden

- Die zuständige Landesärztekammer dokumentiert die erfolgreich abgeleistete Z‑WB „Klinische Akut- und Notfallmedizin“ und die Z‑WB „Notfallmedizin“ mit einer Prüfung.
- Eine zusätzliche Prüfung der Curriculumweiterbildungsinhalte ist nicht vorgesehen.
- Dem in der klinischen Akut- und Notfallmedizin Tätigen und mit der Zusatz-Weiterbildung „Klinische Akut- und Notfallmedizin“ Qualifizierten obliegt die Verantwortung für das „berufslebenslange“ Aufrechterhalten dieser Qualifikation durch Teilnahme an Fortbildungsveranstaltungen und Kursen (z. B. der DGIIN, DIVI, DGINA), durch Kongressbesuche (z. B. Jahrestagung der DGIIN/ÖGIAIN, DIVI, DGINA) und mit kontinuierlichem Fachliteraturstudium [24].
- Eine darüber hinaus gehende Zusatzqualifizierung internistischer Schwerpunktgesellschaften [15] erweitert das Spektrum im Sinne einer weiterführenden Spezialisierung.

## 10. Vorgesehene Aktualisierung des Curriculums

Eine Aktualisierung des Curriculums ist spätestens in 5 Jahren (2029) vorgesehen.

## 11. Ziele des Curriculums: internistische Weiterbildungsinhalte der Akut- und Notfallmedizin umfassend und aktuell präsentieren!

Ziel des Curriculums ist es, die in der Akut- und Notfallmedizin tätigen Ärztinnen und Ärzte in die Lage zu versetzen, bei ihrer Tätigkeit das gesamte Spektrum der Inneren Medizin in Bezug auf Diagnostik, Monitoring und Therapie kompetent beim Patienten anzuwenden. Dies schließt umfassende Kenntnisse, Erfahrungen und Fertigkeiten in der Gesprächsführung mit Patienten und Angehörigen mit ein. Besondere Kenntnisse und praktische Erfahrungen soll der Akut- und Notfallmediziner auch bezüglich der Anwendung von Arzneimitteln bei Notfallpatienten erwerben, da häufig Arzneimittelmetabolismus und -elimination infolge von Organdysfunktionen des Notfallpatienten alteriert sind. Dies gilt insbesondere auch für alte und geriatrische Patienten in der Notaufnahme/in der Notfallklinik, mit Frailty, Sarkopenie, geriatrischen Syndromen, kognitiver Dysfunktion, Polypharmazie und Polypragmasie [25]. Und schließlich wird die zunehmende Digitalisierung in der Akut- und Notfallmedizin auch die Weiterbildungsinhalte und damit auch zukünftige Auflagen dieses Curriculums entscheidend prägen.

### 11.1. Allgemeine Aspekte der klinischen Akut- und Notfallmedizin – Schwerpunkt Innere Medizin

#### 11.1.1. Allgemeiner Teil – Struktur- und Prozessqualität (Tab. 1)

**Table 1:** 

WZ 1	Verständnis für die diversen organisatorischen Aspekte einer Notaufnahme, insbesondere hinsichtlich Patientensicherheit, Kommunikation sowie interprofessionelle und interdisziplinäre Kooperation
*1.1*	*Organisation – Kenntnisse der Zusammensetzung und Aufgabenverteilung in einem multiprofessionellen Notaufnahmeteam*
TK 1	Multiprofessionelle Personalbesetzung in den verschiedenen Schichten
TK 2	Aufgabenverteilung im multiprofessionellen Team der Notaufnahme
BV 1	Konstruktive und kollegiale Mitarbeit im Notaufnahmeteam
*1.2*	*Visiten und Tagesziele*
TK 3	Aufgaben und Inhalte einer multiprofessionellen und interdisziplinären klinischen Visite
TK 4	Ablauf der multiprofessionellen und interdisziplinären Visite (Zeit, Dauer, Ort, Teilnehmer, Dokumentation)
PF 1	*Level III: strukturierte Patientenvorstellung bei der Visite
PF 2	*Level III: Erkennen von Problemen und Erarbeiten von Lösungsvorschlägen
PF 3	*Level III: Formulierung und Dokumentation von Schichtzielen und Tageszielen
PF 4	*Level III: angemessenes Verhalten am Krankenbett
BV 2	Offene Kommunikation bei der Visite mit Berücksichtigung begründeter individueller Ausnahmen und Modifikationen. Bei Bedarf: Erklärung der Notwendigkeit von SOP
BV 3	Einbeziehung der an der Notfallpatientenbetreuung beteiligten Berufsgruppen in die Diskussion und Festlegung der Schichtziele und Tagesziele
*1.3*	*Kommunikation, Teambesprechung, Konfliktmanagement und Krisenintervention*
TK 5	Grundsätze einer respektvollen, am Patientenwohl orientierten Kommunikation
TK 6	Formen und Ablauf von Teambesprechungen
PF 5	*Level III: aktive Teilnahme an den etablierten Teambesprechungen
PF 6	*Level II: Einbeziehung externer Unterstützungsmöglichkeiten bei Konflikten und Krisen (z. B. Psychologen, Ethikkonsil, Seelsorger, Mediatoren)
BV 4	Konstruktives und kollegiales Verhalten im Team
BV 5	Konstruktiver und lösungsorientierter Umgang mit Konflikten
TK 7	Kenntnis der verschiedenen Dokumentationsstandards in der Notaufnahme
PF 7	*Level III: Dokumentation einer Aufnahme
PF 8	*Level III: tägliche Schichtdokumentation
PF 9	*Level III: Dokumentation bei Verlegung und Entlassung – Arztbrief/Notaufnahmeprotokoll
PF 10	*Level III: Dokumentation bei Sterbefällen (inkl. Totenschein)
PF 11	*Level III: Dokumentation: Patientenwillen und Angehörigengespräche
PF 12	*Level III: Dokumentation einer Fixierungsanordnung
BV 6	Gegenseitige konstruktive Einforderung einer genauen Dokumentation aller für den Behandlungsverlauf des Patienten relevanten Daten
*1.4*	*Patientenaufnahme, -verlegung und -entlassung*
TK 8	Kriterien für die Patientenaufnahme, -verlegung und -entlassung
PF13	*Level III: Planung und Organisation von Aufnahme, Verlegung und Entlassung
BV 7	Entscheidung über die Aufnahme und Verlegung von Notfallpatienten auf der Grundlage der vitalen Gefährdung, der Indikation einer stationären Behandlung und der Patientensicherheit
*1.5*	*„Standard operating procedures“ (SOPs)*
TK 9	Kenntnis wichtiger Behandlungs- und Organisationsstandards der Notaufnahme
TK 10	Zugriffsmöglichkeiten auf SOPs der Notaufnahme
PF 14	*Level III: Umsetzung von SOPs
PF 15	*Level II: Mitarbeit an der Überarbeitung bzw. Erstellung von SOPs
BV 8	Indikationsbezogener Einsatz von SOPs
*1.6*	*Rechtliche und ethische Grundlagen*
WZ 2	Kenntnis der rechtlichen und ethischen Grundlagen bei der Versorgung von Notfallpatienten
TK 11	Allgemeine Rechtsgrundlagen und Haftungsfragen, Abmeldung, Behandlungsauftrag
TK 12	Behandlungsumfang, Behandlungsverweigerung, Patientenverfügung, Vorsorgevollmacht und Betreuung
TK 13	Gewalt in der Notaufnahme, Deeskalationsmanagement, Einschränkung der Freiheitsrechte
*1.7*	*Qualitätsmanagement (QM)*
WZ 3	Kenntnisse über Qualitätsmanagement, Risikomanagement und Fehlerkultur in der Notaufnahme
TK 14	Grundlagen des QM-Projektmanagements, Qualitätsindikatoren, Risikomanagement in der klinischen Akut- und Notfallmedizin
TK 15	Grundlagen des Risikomanagements der klinischen Akut- und Notfallmedizin
TK 16	Crew bzw. Team Ressource Management in kritischen Situationen [21]
*1.8*	*Sektorenübergreifende Zusammenarbeit*
WZ 4	Kenntnis der verschiedenen Sektoren und hausinternen/externen Schnittstellen (insbesondere Rettungsdienst) in der klinischen Akut- und Notfallmedizin
TK 17	Rechtsgrundlagen und Organisation von Notfallrettung/Rettungsdienst und des ambulanten Versorgungssektors
TK 18	Notaufnahmen/Krankenhäuser (Versorgungsstufen, strukturräumliche Verteilung und Fachabteilungen) und Sondereinrichtungen
TK 19	Interdisziplinäre Schnittstellen und Organisationsprozesse
BV 9	Kommunikation mit Rettungsdienst und kassenärztlicher Vereinigung
*1.9*	*Ersteinschätzungssysteme*
TK 20	Vor- und Nachteile von mindestens 3 standardisierten Ersteinschätzungssystemen

Anmerkung: Level-I- bis -III-Angaben sind zur Hervorhebung mit einem * versehen. *QM* Qualitätsmanagement, *SOP* „Standard operating procedure“

##### Grundlagen und Standards.

Die Qualität der Zusammenarbeit in dem interprofessionellen und interdisziplinären Team einer Notaufnahme/Notfallklinik/einem Notfallzentrum hat einen erheblichen Einfluss auf die Qualität der dortigen Patientenversorgung und die Patientensicherheit.

##### Das soll gewusst und gekonnt werden.

- Verständnis dafür, welche strukturellen Faktoren und Prozesse diese Qualität beeinflussen und welche Ergebnisse als relevant eingestuft werden müssen. Dazu gehören auch medikolegale Aspekte, wie die Einwilligung in medizinische Eingriffe, das Betreuungsrecht und die Anwendung freiheitsentziehender Maßnahmen sowie die allgemeinen Rechtsgrundlagen und Haftungsfragen.
- Kenntnisse im Qualitätsmanagement, im Risikomanagement und in der Fehlerkultur in der Notaufnahme sowie Kenntnisse der unterschiedlichen Sektoren und Schnittstellen in der klinischen Akut- und Notfallmedizin.
- Umfassender Überblick und Kenntnis der Patientenersteinschätzung, einem der wichtigen organisatorischen Systeme.

#### 11.1.2. Allgemeiner Teil – Erstdiagnostik, Initialtherapie und Indikationsstellung zur weiterführenden Behandlung (Tab. 2)

**Table 2:** 

WZ 1	Eigenständige Durchführung einer rationalen Notfalldiagnostik bei verschiedenen Leitsymptomen, Einleitung einer angemessenen Notfalltherapie sowie sichere Entscheidung über die Notwendigkeit einer stationären Weiterbehandlung mit der Auswahl einer geeigneten Behandlungseinheit
TK 1	Erstdiagnostik, Initialtherapie und Indikationsstellung zur weiterführenden Behandlung bei → Dyspnoe → Herzrasen, Palpitationen und Brustschmerzen → Kopfschmerzen → Schwindel → akuten Störungen des Bewusstseins und Bewusstseinsverlust, Synkopen [27] → Störungen des Gedächtnisses, der Kognition und des Verhaltens → Übelkeit, Erbrechen und Diarrhö → nichttraumatologischen Blutungen [28] → Schock → Dysurie, Oligo‑/Anurie, Polyurie, Hämaturie → akuten Bauch- und Leistenschmerzen → Schmerzen und akuten nichttraumatischen Veränderungen der unteren und oberen Extremitäten → Ikterus → Veränderungen der Körpertemperatur → akuten Hautveränderungen
PF 1	*Level III: rationale, an Leitsymptomen ausgerichtete Notfalldiagnostik und Therapie inkl. notwendigem Monitoring
PF 2	*Level III: sicheres Erkennen der Notwendigkeit einer stationären Weiterbehandlung im Vergleich zur ambulanten Versorgungsmöglichkeit
PF 3	*Level III: sichere Auswahl einer geeigneten Versorgungseinheit für die Notfallpatienten (z. B. Beobachtungsstation, Aufnahmestation, Intensivstation, Intermediate-Care-Station, Normalstation)

Anmerkung: Level-I- bis -III-Angaben sind zur Hervorhebung mit einem * versehen

##### Grundlagen und Standards.

Kernelemente der Arbeit des klinischen Akut- und Notfallmediziners ist die differenzialdiagnostische Abklärung von zur Vorstellung führenden Leitsymptomen unter Berücksichtigung einer zeitkritisch einzuleitenden Notfalltherapie.

##### Das soll gewusst und gekonnt werden.

Nach Stellung einer Arbeitsdiagnose ist die folgende Notfalldiagnostik leitlinienkonform durchzuführen. Weiterhin ist die Entscheidung für oder gegen eine stationäre Aufnahme bzw. eine ambulante Weiterbehandlung zu treffen. Im Fall einer stationären Aufnahme soll der Notfallmediziner die Indikationen/Kriterien für die Notwendigkeit einer engmaschigen Überwachung der Patienten auf einer geeigneten Behandlungseinheit (z. B. Beobachtungsstation) kennen bzw. erkennen [26].

#### 11.1.3. Allgemeiner Teil – Schockraumversorgung (Tab. 3)

**Table 3:** 

WZ 1	Eigenständige Durchführung einer indizierten Schockraumversorgung im multiprofessionellen, interdisziplinären Schockraumteam
TK 1	Erstdiagnostik, Initialtherapie und Indikationsstellung zur weiterführenden Behandlung
PF 1	*Level III: rationale, an Leitsymptomen ausgerichtete strukturierte Notfalldiagnostik und Therapie nach dem ABCDE-Schema („*airway, breathing, circulation, disability, exposure/environment*“)
PF 2	*Level III: Stabilisierung der Vitalfunktionen
PF 3	*Level III: erweitertes Schockraummanagement

Anmerkung: Level-I- bis -III-Angaben sind zur Hervorhebung mit einem * versehen

##### Grundlagen und Standards.

Die Anzahl kritisch kranker, nichttraumatologischer Schockraumpatienten, die in Notaufnahmen bzw. Notfallzentren eingewiesen werden, ist bis zu 4‑mal höher als die Anzahl schwerverletzter Patienten [19]. Ein entsprechendes Weißbuch „Nichttraumatologisches Schockraummanagement“ ist verfügbar [89].

##### Das soll gewusst und gekonnt werden.

Die Wahl einer geeigneten Behandlungseinrichtung, der Einsatz einer adäquaten bildgebender Diagnostik, die Etablierung von Algorithmen und Behandlungspfaden sowie die Koordination und Abstimmung der Übergabe sind unabdingbare Bestandteile der Kenntnisse und Fähigkeiten eines Akut- und Notfallmediziners. Insbesondere die Übergabe von Notfallpatienten bedarf einer gewissen Strukturierung und Vereinheitlichung [29, 30] unter Einbeziehung und Beachtung aller beteiligter Berufsgruppen und Prozesse.

#### 11.1.4. Allgemeiner Teil – Diagnostik und Monitoring (Tab. 4)

**Table 4:** 

*4.1*	*Anamnese*
WZ 1	Eigen- und Fremdanamnese inkl. der Besonderheiten der Übergabe durch den Rettungsdienst
TK 1	Anamnesetechniken
PF 1	*Level III: strukturierte Anamnesedokumentation bei Aufnahme
PF 2	*Level III: Anamneseerhebung im akutmedizinischen Setting nach dem ABCDE- („airway, breathing, circulation, disability, exposure/environment“) bzw. SAMPLER-Schema [36, 37]
PF 3	*Level III: Medikamentenanamnese (inkl. Allergien und Unverträglichkeiten) und Familienanamnese
PF4	*Level II: Transfusionsanamnese (Vortransfusion, Nebenwirkungen unter Transfusion, Knochenmark‑/Stammzelltransplantation, Blutgruppenausweis)
PF 5	*Level II: Impfanamnese (Vorimpfungen, Nebenwirkungen unter Impfungen, Dauer der Schutzwirkung von Impfungen, Impfausweis)
BV 1	Kommunikationstraining
*4.2*	*Körperliche Untersuchung*
WZ 2	Körperliche Untersuchung (Inspektion, Auskultation, Perkussion, Palpation)
TK 2	Untersuchungstechniken bei Bewusstseinsstörungen inkl. Demenz und Delir bzw. akute Enzephalopathie bei kritisch Kranken (leitsymptomorientiert, sequenziell)
PF 6	*Level III: strukturierte Basisuntersuchung von Notfallpatienten bei Aufnahme
PF 7	*Level III: standardisierte Nachuntersuchung von Notfallpatienten im Rahmen der Verlaufsvisitation unter Berücksichtigung aller zur Verfügung stehenden Informationen (z. B. Beatmungssituation, Hämodynamik, neurologischer Status, Zugangssituation, Hautzustand [Dekubitus]), ggf. Integration der bettseitigen Sonographie/Echokardiographie
PF 8	*Level III: Dokumentation der körperlichen Untersuchung
*4.3*	*Basismonitoring*
TK 3	Hämodynamisches Basismonitoring [32, 38]
TK 4	Kenntnisse der messbaren Parameter im Rahmen des Basismonitorings: → Atemfrequenz → (nicht)invasive Blutdruckmessung → Blutgasanalyse → Elektrokardiogramm → Temperaturmessung → Urinproduktion → Pulsoxymetrie → Kapnometrie/Kapnographie → Notfallechokardiographie inklusive Sonographie der V. cava inferior
PF 9	*Level III: strukturierte Erfassung der Parameter des Basismonitorings bei Patientenaufnahme und in der täglichen Routine
PF 10	*Level III: Beurteilung der Hämodynamik im klinischen Kontext
PF 11	*Level III: systematische und einheitliche Dokumentation der Parameter des Basismonitorings
BV 2	Weitergabe der Parameter und deren Konsequenzen an das Behandlungsteam
*4.4*	*Notfall- bzw. fokussierte Sonographie*
WZ 3	Vermittlung der mittels (fokussierter) Sonographie erzielbaren diagnostischen Resultate beim Notfallpatienten
TK 5	Grundlagen der Notfallsonographie [39]
TK 6	Anwendungsgebiete der Notfallsonographie: → Fokussierte Echokardiographie → Fokussierte Thorax- und Abdomensonographie → Bettseitige Gefäßsonographie
PF 12	Erlernen der Sonographiekompetenz in der internistischen Notfall- und Intensivmedizin in Anlehnung an das Positionspapier von DGIIN, DEGUM und DGK [33]: *Level III: Basislevel SIN I: → Leitsymptomorientierte Anwendung der Notfallsonographie → Standardisierte Anwendung der fokussierten Echokardiographie bei Dyspnoe und Thoraxschmerzen → Standardisierte Anwendung der fokussierten Thoraxsonographie bei Dyspnoe → Standardisierte Anwendung der fokussierten Abdomensonographie bei akutem Abdomen *Level II: → Hepatische Sonographie einschließlich der Darstellung der hepatischen Gefäßstrukturen entsprechend Basislevel SIN II [33] *Level II: → Fortgeschrittene Gefäßsonographie entsprechend Basislevel SIN II [33]
PF 13	*Level III: Beurteilung der sono-/echokardiographischen Befunde im klinischen Kontext
PF 14	*Level III: protokollbasierte Dokumentation der erhobenen Befunde (z. B. nach dem ABC-Schema: „*Abdomen*“, „*breath*“ [Thorax], „*cardiac*“); Befundung und Archivierung der Bilddaten [39]
BV 3	Weitergabe der Befunde und deren Konsequenzen an das Behandlungsteam
*4.5*	*Elektrokardiogramm*
TK 7	Grundlagen der EKG-Auswertung (Rhythmus, Lagetyp, De‑/Repolarisationsstörungen, PQ-/QRS-/QTc-Zeiten; [40])
TK 8	EKG-Befunde: → Rhythmusstörungen (Brady‑/Tachykardien, intraventrikuläre Leitungsstörungen) → Akutes Koronarsyndrom (ACS) → (Peri‑)Myokarditis → Elektrolytstörungen → Rechtsherzbelastung → Schrittmacher/ICD-Patienten
PF 15	EKG-Interpretation bei akutem Koronarsyndrom (ACS) → *Level III: charakteristische Zeichen entsprechend der aktuellen Leitliniendefinition eines ACS mit (STEMI) und ohne (NSTEMI) anhaltende ST-Strecken-Hebung → *Level II: schwieriger zu interpretierende Zeichen eines ACS (z. B. modifizierte Sgarbossa-Kriterien bei Linksschenkelblock, ST-Strecken-Hebungen in den rechtspräkordialen Ableitungen [V_3r_, V_4r_] und in den Ableitungen V_7_ bis V_9_)
PF 16	EKG-Interpretation bei lebensbedrohlichen Herzrhythmusstörungen: → *Level III: häufige lebensbedrohliche Herzrhythmusstörungen (z. B. ventrikuläre Tachykardien, Kammerflimmern) → *Level II: seltenere lebensbedrohliche Herzrhythmusstörungen (z. B. Brugada-Syndrom, Kammerarrhythmien durch übergeleitetes Vorhofflimmern/-flattern bei Präexzitationssyndrom)
BV 4	Interdisziplinäre Zusammenarbeit mit Kardiologen
*4.6*	*Labor*
TK 9	Grundlagen der Labordiagnostik und Analysen
TK 10	Möglichkeiten der Labordiagnostik (Zentrallabor, Mikrobiologie, Virologie, Pathologie, Zytologie, Toxikologie, Rechtsmedizin)
TK 11	Phasen der Laboranalytik: → Präanalytische Phase: Indikationsstellung, Probenkennzeichnung, Technik und Reihenfolge der Probenentnahme, Probenlagerung, Transport → Analytische Phase: Zentrallabor, externes Labor, POCT-Verfahren → Postanalytische Phase: Interpretation der Laborergebnisse im klinischen Kontext
PF 17	*Level III: sichere Blutprobengewinnung aus peripheren (z. B. arterielle Katheter) und zentralen Zugängen (z. B. zentrale Venenkatheter [ZVK], Schleusen)
PF 18	*Level III: sichere Probengewinnung aus Kathetern/Drainagen (Urin, Stuhl, Liquor, Pleura‑/Perikarderguss, Aszites etc.)
BV 5	Interdisziplinäre Zusammenarbeit/Interpretation
*4.7*	*Echokardiographie*
TK 12	Grundlagen der Echokardiographie
TK 13	Möglichkeiten der Echokardiographie in der klinischen Akut- und Notfallmedizin: transthorakale (TTE), transösophageale (TEE) und Kontrastechokardiographie [35]
PF 19	Transthorakale Echokardiographie (TTE): → *Level III: Sonographie des Herzens in der Perireanimationsphase bzw. „focused echocardiographic evaluation in life support“ (FEEL) → *Level III: Notfallechokardiographie bzw. fokussierte Echokardiographie (Basislevel, SIN-I [33]) → *Level I: fortgeschrittene Echokardiographie für die Untersuchung kardiologischer Notfallpatienten (Expertenlevel, SIN-II [33])
PF 20	Grundzüge der transösophagealen Echokardiographie (TEE) entsprechend Expertenlevel SIN-II [33]: → *Level I: Standardschnitte inklusive Evaluation von Endokarditis, Aortendissektion, kardialen Emboliequellen (linkes Vorhofohr, LAA), intrakardialen Shunts (persistierendes Foramen ovale [PFO]; Vorhofseptumdefekt [ASD]; Ventrikelseptumdefekt [VSD]; Klappen- und Klappenprothesenvitia)
PF 21	*Level III: Beurteilung der echokardiographischen Befunde im klinischen Kontext
PF 22	*Level III: protokollbasierte Dokumentation und Archivierung der Bilddaten
BV 6	Weitergabe der Befunde und deren Konsequenzen an das Behandlungsteam
*4.8*	*Röntgendiagnostik*
TK 14	Röntgendiagnostik in der Notfallmedizin
TK 15	Grundlagen der Strahlenexposition und des Strahlenschutzes
TK 16	Aufnahmetechniken der Liegend- und Stehendlungenaufnahmen, der Abdomenübersicht und der Abdomenaufnahme in Linksseitenlage
PF 23	*Level II: Überprüfung der rechtfertigenden Indikation einer Thorax- und Abdomenaufnahme unter Berücksichtigung des aktuellen Strahlenschutzgesetzes
BV 7	Interdisziplinäre Zusammenarbeit bei Indikationsstellung und Interpretation der Befunde mit Radiologen
BV 8	Möglichkeiten der Teleradiologie/Befundübermittlung
BV 9	Vermeidung von Überdiagnostik
*4.9*	*Computertomographie (CT) und Magnetresonanztomographie (MRT)*
TK 17	Physikalische Grundlagen der CT und MRT
TK 18	CT- und MRT-Diagnostik bei Notfallpatienten
PF 24	*Level II: Überprüfung der rechtfertigenden Indikation einer Notfall-CT unter Berücksichtigung des aktuellen Strahlenschutzgesetzes und der Leitlinie der BÄK zur Qualitätssicherung in der Computertomographie
PF25	*Level II: Überprüfung der Indikation einer Notfall-MRT zusammen mit dem Radiologen und der jeweiligen Fachabteilung (z. B. Neurologie) unter Berücksichtigung der BÄK-Richtlinie zur Qualitätssicherung bei der MRT
PF 26	*Level III: Planung/Vorbereitung eines Transports kritisch kranker Patienten; checklisten-/protokollbasierter Patiententransport inklusive Komplikationsmanagement, unter Berücksichtigung der Empfehlung der DIVI zum innerklinischen und Interhospitaltransport kritisch kranker erwachsener Patienten [41]
PF 27	*Level III: Begleitung/Betreuung kritisch kranker Patienten im Notfallteam inkl. Protokollierung
BV 10	Interdisziplinäre Zusammenarbeit/Interpretation mit der Radiologie und der jeweiligen Fachabteilung
BV 11	Möglichkeiten der Teleradiologie/Befundübermittlung
BV 12	Vermeidung von Überdiagnostik
*4.10*	*Bronchoskopie*
TK 19	Grundlagen der flexiblen Bronchoskopie
TK 20	Voraussetzungen, Indikationen und Kontraindikationen der flexiblen Bronchoskopie bei nichtbeatmeten Notfallpatienten
TK 21	Voraussetzungen, Indikationen und Kontraindikationen der flexiblen Bronchoskopie bei beatmeten Notfallpatienten: Auswirkungen auf Atemmechanik, Gasaustausch und Hämodynamik
PF 28	*Level II: flexible Bronchoskopie bei stabilen beatmeten Patienten *Level I: flexible Bronchoskopie bei stabilen nichtbeatmeten Patienten (Wachbronchoskopie) und bei instabilen beatmeten Patienten
PF 29	*Level III: Gewinnung von Trachealsekret oder Entfernung eines Fremdkörpers
BV 13	Interdisziplinäre Zusammenarbeit mit Pneumologen/Radiologen/Infektiologen
*4.11*	*Endoskopische Verfahren*
TK 22	Hintergrundwissen endoskopischer Verfahren in der Gastroenterologie
TK 23	Indikationen der einzelnen Verfahren und deren Dringlichkeit
PF 30	*Level III: Monitoring, Analgesie und Sedierung sowie Management von kritisch kranken Patienten in der gastrointestinalen Endoskopie während Gastroskopie, Koloskopie und anderen endoskopischen Verfahren unter Berücksichtigung der aktuellen S3-DAS-Leitlinie [42]
PF 31	*Level III: protokollbasierte Dokumentation von Monitoring und Analgosedierung
BV 14	Interdisziplinäre Zusammenarbeit mit Gastroenterologen
*4.12*	*Neurologische Verfahren*
TK 24	Neurologisch-diagnostische Verfahren in der Notfallmedizin
TK 25	Bildgebende neurologische Verfahren: kraniale CT (cCT) und kraniale MRT (cMRT) sowie Angiographie hirnversorgender und zerebraler Gefäße
PF 32	**Strukturierte neurologische Untersuchung** (Bewusstseinsstörungen, Motorik/Reflexstatus, Sensibilität/Dermatome, Hirnnervenstatus, Koordination, Meningismuszeichen): → *Level III: beim kritisch kranken Patienten → *Level I: bei neurologischen Symptomen/Syndromen
PF 33	*Level II: Durchführung einer bettseitigen Liquorpunktion
BV 15	Interdisziplinäre Zusammenarbeit bei der Befundinterpretation mit Neurologen/Neurochirurgen/Neuroradiologen
*4.13*	*Hirntoddiagnostik*
TK 26	Medizinische und rechtliche Grundlagen der Hirntoddiagnostik
BV 16	Zusammenarbeit mit dem weiterbetreuenden interdisziplinären Team
BV 17	Gesprächsführung mit Angehörigen und Angehörigenbetreuung

Anmerkung: Level-I- bis -III-Angaben sind zur Hervorhebung mit einem * versehen

##### Grundlagen und Standards.

- **Standardrepertoire: **Die Akutdiagnostik und das Monitoring gehören zum Standardrepertoire der notfallmedizinischen Betreuung von Notfallpatienten inkl. kritisch kranker Patienten. Im Rahmen der Diagnostik soll die gezielte (Fremd‑)Anamnese sowie die körperliche Untersuchung und damit der „klinische Blick“ stets die Basis bei der Aufnahme eines Notfallpatienten darstellen. Bei der Anamneseerhebung hat die Interaktion mit dem Rettungsdienst inkl. das Verständnis für die im Rettungsdienst eingesetzten Übergabealgorithmen eine besondere Bedeutung. Wesentliche Fähigkeiten des Notfallmediziners sind das rasche Stellen einer Arbeitsdiagnose unter Berücksichtigung zeitkritischer und potenziell lebensbedrohlicher Differenzialdiagnosen, das schnelle Einleiten geeigneter Notfalltherapien sowie die Entscheidung über die Notwendigkeit einer stationären Weiterbehandlung und die Festlegung eines geeigneten Stationstyps [26].
- **Diagnostik: **In der Notfallmedizin findet die bildgebende Diagnostik überwiegend am Krankenbett statt – „*bedside ultrasonography*“ (fokussierte Sonographie [31]), da Transporte ggf. mit einem erhöhten Patientenrisiko oder einer Zeitverzögerung verbunden sind. Alle diagnostischen Verfahren sollten stets im klinischen Kontext sowie im interdisziplinären fachärztlichen Team erfolgen.
- Monitoring:
  - Für das **Monitoring** stehen dem Notfallmediziner sowohl nichtinvasive als auch invasive Werkzeuge zur Verfügung. Die Europäische Gesellschaf für Intensivmedizin (ESICM) empfiehlt ein klinisches sowie ein hämodynamisches Monitoring auch zur Identifizierung der Schockursache und zur Überprüfung der therapeutischen Maßnahmen und des Ansprechens auf die Therapie [32]. Das Basismonitoring soll die klinische Untersuchung (z. B. Symptome/Zeichen von Stauung und Hypoperfusion bei akuter Herzinsuffizienz) und nichtinvasive Überwachungsverfahren (z. B. die Messung der peripheren Sauerstoffsättigung) beinhalten. Das erweiterte Monitoring reduziert sich in der Notaufnahme zumeist auf eine kontinuierliche invasive Blutdruckmessung. Ergänzende hämodynamische Messverfahren sollen auf der Intensivstation durchgeführt werden.
  - Das **Basismonitoring** soll immer 2 unabhängig voneinander agierende Vitalparameter des Patienten einbeziehen und beinhaltet neben der Bestimmung der Atemfrequenz, der Körpertemperatur sowie der peripheren Sauerstoffsättigung die nichtinvasive Blutdruckmessung (ggf. invasiv), die Beurteilung des 12-Ableitungs-Elektrokardiogramms (EKG) und auch Grundkenntnisse in der leitsymptomorientierten Notfallsonographie.
  - Ziel der **fokussierten Notfallsonographie inkl. Echokardiographie** ist das frühzeitige Erkennen bzw. der Ausschluss wichtiger kritischer Diagnosen. Bei den allermeisten Leitsymptomen kann die bettseitige Notfallsonographie hier eine rasche Diagnose z. B. des Pneumothorax bei Dyspnoe oder der Cholezystitis bei akutem Abdomen ermöglichen. Die strukturierte Ultraschallausbildung in der internistischen Intensiv- und Notfallmedizin (SIN) basiert auf einem 2‑Stufen-Konzept, das von den 3 nationalen Fachgesellschafen DGIIN, DGK und DEGUM vertreten wird [33–35]. Das Konzept umfasst ein Basislevel (SIN-I) und ein Expertenlevel (SIN-II), die – aufeinander aufbauend mithilfe moderner Lehrmethoden – eine leitsymptomorientierte Sonographie für die Notfall- und Intensivmedizin vermitteln. Es werden sowohl theoretische Kenntnisse als auch praktische Fertigkeiten gelehrt und im Rahmen einer Prüfung kontrolliert. Ziel ist es, die in nationalen und internationalen Leitlinien empfohlenen Vorgaben zum Einsatz der Sonographie in der Notfall- und Intensivmedizin in der klinischen Praxis standardisiert zu etablieren.

##### Das soll gewusst und gekonnt werden.

- Anamnese, körperliche Untersuchung und sämtliche bettseitige Untersuchungen, wie EKG, Labordiagnostik (u. a. „*Point-of-care-testing*“[POCT]-Verfahren), fokussierte Sono‑/Echokardiographie sowie das Monitoring, bilden die Basis der notfallmedizinischen Betreuung von Notfallpatienten inklusive der kritisch kranken Patienten. Neben diesen Inhalten sollen die Grundlagen sämtlicher radiologischer Untersuchungsverfahren (Röntgen, Computertomographie, Magnetresonanztomographie) von der Überprüfung der Indikation bis hin zur Planung bzw. Durchführung eines Patiententransports (Intra- und Interhospitaltransport) nach den Regeln eines Intensivtransports (inklusive Komplikationsmanagement) in der Zusatz-Weiterbildung vermittelt werden.
- Der Akut- und Notfallmediziner soll in der Lage sein, selbstständig und eigenverantwortlich eine fokussierte Sonographie inkl. Echokardiographie, abhängig vom jeweiligen Ausbildungslevel (beginnend von „*focused echocardiography in emergency life support*“ [FEEL] bis hin zu Grundzügen der transösophagealen Echokardiographie) durchzuführen. Weiterhin sollte er eine flexible Bronchoskopie im Rahmen des Atmungs- und Beatmungsmanagements durchführen können. Häufige Punktionen – insbesondere Pleura- und Aszitespunktion – sollen selbstständig durchgeführt werden. Hinsichtlich der Hirntoddiagnostik zur Feststellung des irreversiblen Hirnfunktionsausfalls soll er über die theoretischen Kenntnisse verfügen, um gemeinsam mit dem interdisziplinären Team die Indikation für eine organerhaltende Therapie initiieren zu können.

#### 11.1.5. Allgemeiner Teil – generelle Therapieverfahren (Tab. 5)

**Table 5:** 

WZ 1	Für den Notfallmediziner relevante Therapieverfahren: Vermittlung von Kenntnissen, Fähigkeiten und professionellem Verhalten
*5.1*	*Venöse und arterielle Zugänge*
WZ 2	Vermittlung von Kenntnissen und Fähigkeiten zur Anlage arterieller und venöser Zugänge
TK 1	Grundlagen zur Vorbereitung und Durchführung eines Gefäßzugangs (Patientenlagerung, hygienische Maßnahmen, Seldinger-Technik)
TK 2	Indikationen verschiedener Zugangswege (PVK, ZVK, arterielle Zugangswege, spezielle Schleusen und Zugänge für Dialysezugänge sowie Zugänge für ECLS/ECMO)
PF 1	*Level III: sichere Durchführung üblicher und häufiger notfallmedizinischer Zugänge: PVK, ZVK (V. jugularis, V. subclavia, V. femoralis), arterielle Zugänge (A. radialis, A. femoralis)
*5.2*	*Volumentherapie*
WZ 3	Vermittlung der physiologischen und pathophysiologischen Grundlagen der Infusionstherapie und Abgrenzung der Kreislauftherapie von reiner Flüssigkeitsgabe sowie klinische Einschätzung des Volumenstatus kritisch kranker Patienten
TK 3	Indikationen zur Infusionstherapie
TK 4	Zusammensetzung der Flüssigkeitskompartimente des Körpers: Gesamtkörperwasser (intrazellulär, extrazellulär, intravasal)
TK 5	Unterscheidung von Volumentherapie (als Kreislauftherapie) und Flüssigkeitsgabe (als Substitution von Verlusten)
TK 6	Zusammensetzung der unterschiedlichen Infusionslösungen; Unterschiede von kolloidalen und kristalloiden Lösungen und deren Indikationen/Kontraindikationen
TK 7	Indikationsstellung des Einsatzes extrakorporaler Therapieverfahren zur Steuerung des Volumenhaushalts eines Patienten
PF 2	*Level III: Einschätzung des Volumenstatus eines Notfallpatienten inkl. der Anwendung unterschiedlicher Verfahren – klinischer Status und Anamnese, Laborparameter, Ultraschallverfahren (inkl. Echokardiographie) – und invasive Verfahren wie die arterielle Blutdruckmessung
PF 3	*Level III: adäquate Einschätzung von Volumenmangel vs. -bedarf und Volumenreagibilität
PF 4	*Level III: regelmäßige Kontrolle des Volumenstatus
*5.3*	*Vasoaktive Substanzen und Inotropika*
TK 8	Pharmakologische Eigenschaften von in der Notfallmedizin eingesetzten Vasopressoren, Vasodilatatoren, Inotropika und Inodilatatoren
TK 9	Pathophysiologische Grundlagen des differenzierten Einsatzes vasoaktiver Substanzen und Inotropika bei den verschiedenen Formen des Schocks, inkl. der damit verbundenen Risiken, insbesondere bei akut oder chronisch kardial erkrankten Patienten
PF 5	*Level III: individualisierte Festlegung von klinischen und hämodynamischen Zielgrößen und des dafür notwendigen Monitorings für die Steuerung einer Therapie mit vasoaktiven Substanzen und Inotropika
*5.4*	*Kardiale Elektrotherapie (Defibrillation, Schrittmacher, Kardioversion)*
WZ 4	Leitlinienorientiertes Management von Patienten mit bedrohlichen Herzrhythmusstörungen einschließlich deren Risikostratifizierung mit Vermittlung und Beherrschen der erforderlichen Kenntnisse und Fähigkeiten der notfallmedizinischen Behandlung
TK 10	Bradykarde und tachykarde Herzrhythmusstörungen
TK 11	Leitliniengerechte Indikationsstellung der kardialen Elektrotherapie
TK 12	Kardiale Elektrotherapie inklusive Defibrillation, Kardioversion, transthorakaler und invasiver Notfallschrittmachertherapie
PF 6	*Level III: sichere EKG-Interpretation bei Patienten mit lebensbedrohlichen Herzrhythmusstörungen
PF 7	*Level III: Durchführung einer Defibrillation/Kardioversion
PF 8	*Level III: Durchführung einer Notfallschrittmacheranlage (invasiv oder transthorakal)
BV 1	Interdisziplinäre Zusammenarbeit mit Kardiologen und ggf. Überweisung in ein kardiologisches Interventionszentrum
*5.5*	*Sauerstofftherapie*
WZ 5	Vermittlung von Kenntnissen und Fähigkeiten der Sauerstofftherapie bei kritisch kranken Patienten
TK 13	Wirkungsweise von konventioneller und High-flow-Sauerstofftherapie
TK 14	Prinzipien der konventionellen und High-flow-Sauerstofftherapie: → Indikationen und Kontraindikationen → Nebenwirkungen, Komplikationen und Limitationen
PF 9	*Level III: Einleitung der High-flow-Sauerstofftherapie
BV 2	Interprofessionelle Zusammenarbeit mit dem Notfallteam
*5.6*	*Atemwegsmanagement*
WZ 6	Vermittlung der erforderlichen Kenntnisse und Fähigkeiten zur Beherrschung des Atmungs- und Beatmungsmanagements bei Notfallpatienten
TK 15	**Indikationen und Kenntnisse zur Durchführung:** → Sicherung des Atemwegs → Präoxygenierung → Notfall- vs. elektive Analgosedierung → Monitoring und Erfolgskontrolle → Atemwegssicherung → Maskenbeatmung → Atemwegshilfen → Erschwerte Maskenbeatmung → Erweiterte Atemwegshilfen → Koniotomie → Videolaryngoskopie → Schwieriger Atemweg (fiberoptische Intubation) → Nasale Intubation
PF 10	*Level III: suffiziente Maskenbeatmung
PF 11	*Level III: sichere Intubation
PF 12	*Level III: Atemwegssicherung
PF 13	*Level II: Koniotomie (Simulationstraining)
PF 14	*Level I: nasale Intubation
PF 15	*Level II: fiberoptische Intubation (Simulationstraining)
BV 3	Interdisziplinäre Zusammenarbeit mit den jeweiligen Fachdisziplinen
*5.7*	*Beatmungsmanagement*
WZ 7	Erwerb von Kenntnissen und Fähigkeiten zur differenzierten Beatmung kritisch kranker Patienten
TK 16	Technische Wirkungsweise von Beatmungsgeräten
TK 17	**Prinzipien der nichtinvasiven Beatmung **[48]: → Indikationen und Kontraindikationen → Nebenwirkungen, Komplikationen und Limitationen → Vor- und Nachteile verschiedener Interfaces → Beatmungseinstellungen (Flow-Kurve, Lungenmechanik)
TK 18	**Prinzipien der invasiven Beatmung:** → Indikationen und Kontraindikationen → Nebenwirkungen, Komplikationen und Limitationen → Prinzip der lungenprotektiven invasiven Beatmung → Prinzip des „Best-PEEP“ → Beatmungsstrategien mit differenziertem Einsatz unterschiedlicher Beatmungsmodi der assistierten und kontrollierten Beatmung → Beatmungseinstellungen entsprechend der Krankheitsphase und der Art des respiratorischen Versagens (hypoxämische und ventilatorische Insuffizienz)
PF 16	*Level III: Einleitung der nichtinvasiven Beatmung (Interface, Eingewöhnung)
PF 17	*Level III: invasive Beatmung: → Einstellung und Anpassung von F_i_O_2_ → Beatmungsmodi → Beatmungsdrucke und Beatmungsfrequenzen → Erkennen und Anpassung von Patient-Ventilator-Dyssynchronisation → Triggereinstellungen → Leckagemanagement
BV 4	Interprofessionelle Zusammenarbeit mit dem Notfallteam
*5.8*	*Koniotomie*
WZ 8	Vermittlung von Kenntnissen und Fähigkeiten der notfallmäßigen tiefen Kehlkopferöffnung
TK 19	Indikation und Kontraindikationen der Koniotomie
TK 20	Komplikationen der Koniotomie
TK 21	Vor- und Nachteile der notfallmäßigen Tracheotomie
TK 22	Grundlagen zur Vorbereitung, Durchführung und Nachsorge der Koniotomie
PF 18	*Level I: praktische Erfahrung mit der Vorbereitung und Durchführung der Koniotomie
PF 19	*Level III: praktische Erfahrung mit dem Trachealkanülenmanagement, dem Wechsel einer Trachealkanüle und der Dekanülierung
*5.9*	*Analgosedierung*
WZ 9	Vermittlung der erforderlichen Kenntnisse und Fähigkeiten für die indikations- und leitlinienorientierte Analgosedierung des kritisch Kranken bzw. intensivpflichtigen Notfallpatienten [42]
TK 23	Pharmakologische Eigenschaften notfallmedizinisch eingesetzter Analgetika [49]
TK 24	Pharmakodynamik, Pharmakokinetik und Nebenwirkungen notfallmedizinisch eingesetzter i.v.-Sedativa
TK 25	Indikationen und Kenntnisse zur Durchführung (Techniken, Risiken): → der Notfall- vs. elektiven Einleitung und Führung der Analgosedierung → der kurzzeitigen (Analgo‑)Sedierung für Endoskopien und bettseitige Eingriffe
PF 20	*Level III: leitlinienorientierte nichtpharmakologische und pharmakologische Therapie von Schmerzen
PF 21	*Level III: Einsatz von Analgetika und Sedativa im Rahmen einer palliativmedizinischen Akutbehandlung
PF 22	*Level III: sichere Einleitung der Analgosedierung zur Intubation sowie kurzzeitige Analgosedierung inkl. periprozeduralem Kreislauf- und Atemwegsmanagement
PF 23	*Level II: differenzierter Einsatz von Analgetika zur Schmerzkontrolle unter Berücksichtigung des längerfristigen Abhängigkeitspotenzials
PF 24	*Level II: Differenzierter Einsatz von Sedativa unter Berücksichtigung der wechselseitigen Beziehung von Agitation, Schmerz und Delir entsprechend der aktuellen S3-DAS-Leitlinie [42] einschließlich der Lokal‑, Oberflächen- und Regionalanästhesie
*5.10*	*Nierenersatzverfahren*
WZ 10	Grundsätzliche Kenntnisse zum Einsatz von Nierenersatzverfahren sowohl im Sinne der evidenzbasierten als auch der personalisierten Medizin
TK 26	Grundsätzliche Kenntnisse zu Indikationen sowie Kontraindikationen von Nierenersatzverfahren und anderen extrakorporalen Blutreinigungsverfahren
TK 27	Grundsätzliche Kenntnisse zu üblichen Antikoagulationsmodi extrakorporaler Verfahren (systemisch und regional) und deren Nebenwirkungen bzw. Komplikationen
PF 25	*Level II: Indikationen, Kontraindikationen, Zugangswege und Techniken der Anlage von Akutdialysekathetern
BV 5	Fähigkeit zur engen Zusammenarbeit mit Nephrologen bei der Indikationsstellung und Durchführung von Nierenersatzverfahren
*5.11*	*„Extracorporeal life support“ (ECLS)/extrakorporale Membranoxygenierung (venovenöse/venoarterielle ECMO [vv-/va-ECMO])*
WZ 11	Vermittlung der physiologischen, pathophysiologischen und klinischen Grundlagen des Einsatzes temporärer Lungen(vv-ECMO)- und Herz-Lungen(va-ECMO)-Ersatzverfahren
TK 28	Grundlagen: → Determinanten des Gasaustauschs und Interaktion mit der maschinellen Beatmung → Grundlagen der ECMO-Therapie (primär venoarteriell) → Information über das zu erwartende Outcome und die Risikoklassifikation beim akuten Herzversagen (Reanimation, kardiogener Schock) → Komplikationen extrakorporaler Systeme und deren Behandlung → Gerinnungsdiagnostik und Antikoagulation im Rahmen der ECMO-Therapie
TK 29	VA-ECMO/ECLS: leitlinienorientierte Indikationsstellung und Wissen um die Grenzen des Einsatzes des temporären Herzersatzes bei links- und/oder rechtsventrikulärem Pumpversagen [50] sowie eCPR [17]
BV 6	Gesprächsführungsstrategien mit Angehörigen, „End-of-life“-Entscheidungen (Eruierung des potenziellen Patientenwillens, Möglichkeiten der Organspende), Arzt-Arzt-Gespräch zur Verlegung in ein regionales ECMO-Zentrum
*5.12*	*Antiinfektive Therapie*
WZ 12	Rationale antiinfektive Therapie: Indikationen, Komplikationen, Zeitpunkt der Beendigung
TK 30	Grundlagen der mikrobiellen Diagnostik (Blutkultur, bronchoalveoläre Lavage [BAL] u. a.) sowie der Infektionsepidemiologie und deren Bewertung
TK 31	Empirische und gezielte antimikrobielle Therapie: Grundlagen der Antibiotika-, antiviralen, antimykotischen und antiparasitären Therapie
PF 26	*Level III: Materialgewinnung zur mikrobiologischen Diagnostik (Blutkulturabnahme, intravasale Katheter, Material aus möglicherweise infiziertem Gewebe)
PF 27	*Level III: Differenzierung von Infektion, Kontamination und Besiedlung
PF 28	Auswahl einer kalkulierten antiinfektiven Therapie in Abhängigkeit von Grunderkrankung und Risikoprofil des Patienten sowie von dem zu erwartenden Keimspektrum: → *Level III: bei Standardsituationen → *Level II: bei komplexen Fragestellungen
PF 29	Antiinfektive Therapie: → *Level III: bei identifiziertem Erreger: antiinfektive Therapie nach Erregersensitivitäts-/-resistenzspektrum und lokaler Resistenzlage → *Level II: bei komplexen Fragestellungen
BV 7	Interdisziplinäre Kooperation mit Hygienikern/Mikrobiologen/Infektiologen (Antibiotic Stewardship [ABS]) und ggf. mit den mitbetreuenden Fachdisziplinen beim individuellen Patienten
BV 8	Zusammenarbeit mit Hygienikern/Mikrobiologen/Infektiologen vor Ort zur Infektionsprophylaxe
*5.13*	*Prophylaxemaßnahmen*
WZ 13	Erlernen der notwendigen prophylaktischen Maßnahmen zur Vermeidung bekannter häufiger Komplikationen bei Notfallpatienten inkl. Dekubitus, Mobilität und Kontrakturen
TK 32	Allgemeine Kenntnisse zur Prophylaxe, Komplikationsvermeidung und Risikominimierung bei Notfallpatienten
TK 33	Spezielle Kenntnisse zu vermeidbaren Komplikationen und Risiken in der Notfallmedizin (z. B. Delirentwicklung bei Demenz, Alkoholmissbrauch) und deren Prophylaxe (z. B. Thromboembolieprophylaxe, Indikation zur Sturzprophylaxe)
PF 30	*Level III: Erkennen der patientenspezifischen Risikofaktoren und erkrankungsspezifischen Komplikationen
PF 31	*Level III: Erstellung eines Behandlungsplans unter Berücksichtigung der medikamentösen und nichtmedikamentösen Prophylaxemaßnahmen
BV 9	Der Komplikationsvermeidung dient die interdisziplinäre und interprofessionelle Zusammenarbeit von Notfallmedizinern mit Notfallfachpflegekräften und denjenigen Mitarbeitern weiterer Berufsgruppen, die in die Betreuung des Patienten involviert sind
BV 10	Weiterführende Kommunikation mit denjenigen Einheiten – z. B. Mikrobiologie und Hygiene –, die in die Betreuung des jeweiligen individuellen Patienten mit involviert sind
*5.14*	*Blutprodukte*
WZ 14	Erlernen und praktische Umsetzung der Indikationen, Kontraindikationen und Komplikationen der Transfusion von Blutprodukten in der Notaufnahme
TK 34	Transfusionstrigger/Indikationen zur Gabe von Blutprodukten (Erythrozyten, Thrombozyten)
TK 35	Nebenwirkungen und Kontraindikationen der Transfusionen von Blutprodukten
TK 36	Indikationen zur Gabe von Gerinnungsfaktoren und -produkten
TK 37	Nebenwirkungen und Kontraindikationen der Gabe von Gerinnungsfaktoren und -produkten
TK 38	Transfusionsgesetze und deren organisatorische Umsetzung und Einhaltung
PF 32	*Level III: praktische und organisatorische Umsetzung der Transfusionsgesetze nach der Richtlinie der BÄK
PF 33	*Level III: Indikationsstellung und Triggergrenzen für die Transfusion von Blut- und Gerinnungsprodukten
PF 34	*Level III: Beherrschung von Nebenwirkungen bzw. Transfusionsreaktionen
PF 35	*Level II: Dokumentation und Meldung unerwünschter Transfusionsreaktionen in Kooperation mit dem Transfusionsbeauftragten
BV 11	Interprofessionelle Zusammenarbeit mit dem Hersteller der Blutprodukte/Blutbank
*5.15*	*Ethik*
WZ 15	Ärztliche Indikation und Patientenwille als Grundlagen sämtlicher Behandlungsentscheidungen nach Festlegung eines patientenzentrierten Therapieziels sowie Bedeutung der (juristischen) Stellvertreter/Angehörigen bei der Ermittlung des Patientenwillens; Steuerung der kommunikativen Abläufe innerhalb des Behandlungsteams und mit den (juristischen) Stellvertretern/Angehörigen [46, 47]
*5.16*	*„End-of-life“-Entscheidungen*
TK 39	Prinzipienethik (Respekt der Autonomie bzw. Selbstbestimmung des Patienten, Prinzip der Schadensvermeidung, Prinzip der Fürsorge, Prinzip der Gerechtigkeit)
TK 40	Ärztliche Indikationsstellung (vor dem Hintergrund von „Nutzen vs. Schaden“)
TK 41	Patientenwille (aufgeklärte Einwilligung/Verweigerung, Patientenverfügung, Vorsorgebevollmächtigter, Betreuer, mutmaßlicher Patientenwille, Behandlungswünsche)
TK 42	Therapiezieländerung (Therapiebegrenzung, Therapiebeendigung)
TK 43	Einberufung und Durchführung einer Ad-hoc-Ethikfallberatung
TK 44	Palliativmedizinische Grundprinzipien
TK 45	Aktive/passive/direkte/indirekte Sterbehilfe
PF 36	*Level III: Fähigkeit zur praktischen Anwendung der Prinzipienethik bei kritisch kranken Patienten mit einer hohen Sterbewahrscheinlichkeit
PF 37	*Level III: Anwendung der ärztlichen Indikationsstellung und Fähigkeit zur Begründung der Indikation vor dem Hintergrund von „Nutzen vs. Schaden“
PF 38	*Level III: Fähigkeit zur Interpretation der Patientenverfügung, auch unter Berücksichtigung der Vorgaben des Betreuungsrechts (§ 1901 a–c des BGB, Ehegattennotvertretung)
PF 39	*Level III: Fähigkeit zur Umsetzung einer Therapiezieländerung
PF 40	*Level III: Beteiligung an einer Ethikfallberatung
BV 12	Fähigkeit zur interprofessionellen und interdisziplinären Diskussion von ärztlicher Indikation, Patientenwille und Therapieziel mit nachfolgender interprofessioneller/interdisziplinärer Entscheidungsfindung
*5.17*	*Angehörigenmanagement*
TK 46	Betreuungsrecht, Patientenverfügung und Vorsorgevollmacht
TK 47	Bedeutung und Durchführung einer Angehörigenbesprechung
TK 48	Implementierung einer angehörigenzentrierten Organisation der Abläufe in einer Notaufnahme (Besuchszeiten, Einbindung der Angehörigen in die Pflege)
TK 49	Akute Belastungen der Angehörigen und mögliche Langzeitfolgen (Angst, Depression, posttraumatische Belastungsstörung)
PF 41	*Level III: strukturierte Angehörigenbesprechung
BV 13	Durchführung einer interdisziplinären und interprofessionellen Angehörigenbesprechung unter Einbeziehung aller an der Behandlung des Patienten beteiligten Berufsgruppen, Fachabteilungen sowie möglicherweise auch der betreuenden Hausärzte

Anmerkung: Level-I- bis -III-Angaben sind zur Hervorhebung mit einem * versehen

##### Grundlagen und Standards.

Die Betreuung eines Notfallpatienten erfordert ein breites Spektrum an allgemeinen Therapieverfahren, das weit über das übliche Spektrum bei der Behandlung einer speziellen internistischen Erkrankung im stabilen Zustand des Patienten hinausgeht. Die Entscheidung zur Therapie hat dabei rasch und gezielt zu erfolgen und erfordert den Erwerb spezieller manueller Fähigkeiten.

##### Das soll gewusst und gekonnt werden.

- **Kathetertechnik:** Die sonographieunterstützte Anlage zentralvenöser (V. jugularis, V. subclavia, V. femoralis) und arterieller (A. femoralis, A. radialis, A. brachialis) Zugänge sowie verschiedene Punktionstechniken und Drainageanlagen (Pleura, Perikard, Aszites, Liquor) sollen während der Z‑WB erlernt und sicher beherrscht werden.
- **Hämodynamische Therapie:** Einer differenzierten hämodynamischen Therapie mit verschiedenen Volumenersatzstoffen und vasoaktiven Substanzen kommt bei den verschiedenen Schockformen vor allem in der Akutphase einer kritischen Erkrankung eine zentrale Rolle zu. Zur hämodynamischen Therapie gehört eine profunde klinische Einschätzung ebenso dazu wie ein leitlinienorientiertes hämodynamisches Monitoring (Tab. 4, Top 4.3) unter Einbeziehung von Zielparametern der Organperfusion [32, 38]. Die sonographische Point-of-care-Evaluation ist ein elementarer Bestandteil (Tab. 4, Top. 4.4). Eine kardiale Elektrotherapie (Defibrillation, Schrittmacher, Kardioversion) soll der Notfallmediziner sicher durchführen können. Bezüglich der venoarteriellen extrakorporalen Membranoxygenierung im Rahmen der Reanimation (eCPR) sind theoretische und praktische Kenntnisse Bestandteil dieses Curriculums. Notfallmediziner in Notaufnahmen, in denen diese mechanische Herz-Kreislauf-Unterstützungssysteme unter Reanimationsbedingungen oder im kardiogenen Schock eingesetzt werden, sollen sich die für die Mitbetreuung dieser Patienten erforderlichen praktischen Fähigkeiten strukturiert aneignen (extrakorporaler Life Support: [43]; extrakorporale Reanimation: [17]).
- **Respiratorische Therapie:** O_2_-Therapie [44] und ein modernes Atemwegsmanagement spielen häufig eine zentrale Rolle in der Akutversorgung eines Notfallpatienten. Hier sollen die notfallmäßige Sicherung der Atemwege einschließlich endotrachealer Intubation sowie der Algorithmus bei schwieriger Intubation, inkl. alternativer Atemwege wie z. B. Larynxtubus und Larynxmaske, aber auch die Koniotomie sicher beherrscht werden. Eingehende Kenntnisse in der Atemphysiologie bis hin zum leitlinienorientierten und differenzierten Atmungs- und Beatmungsmanagement sind zentrale Ziele der Z‑WB. Hiermit gehen auch Kenntnisse in der modernen Analgosedierung einher.
- **Organersatzverfahren:** Zentraler Bestandteil der Notfallmedizin ist das Erkennen der Notwendigkeit des Einsatzes von Organersatzverfahren. Während es in der klinischen Akut- und Notfallmedizin primär um die Prävention/Abmilderung des Organversagens geht, beschäftigt sich der Intensivmediziner mit dem Ersatz der Organfunktion bis zu deren Rekompensation. Dennoch soll auch der Notfallmediziner die wichtigsten Indikationen zum Nierenersatzverfahren (z. B. Intoxikation, lebensbedrohliche Hyperkaliämie) kennen. (siehe auch Abschn. 11.9, Tab. 15). Auch die Anlage verschiedener Zugänge zur Durchführung entsprechender extrakorporaler Verfahren unter Reanimationsbedingungen oder im kardiogenen Schock gehört zum Tätigkeitsfeld.
- **Infektions‑/Sepsistherapie:** Eine zentrale Rolle in der klinischen Akut- und Notfallmedizin kommt schweren Infektionskrankheiten bis hin zur Sepsis und zum septischen Schock zu (siehe auch Abschn. 11.7, Tab. 13). Hier soll der internistische Notfallmediziner eine entsprechend rasche Diagnostik und Therapieeinleitung gemäß den „sepsis bundles“ und Sepsisleitlinien [45] initiieren und durchführen können. Hier kommt gerade der ersten Stunde nach Sepsisdiagnose eine große Bedeutung zu („1 h bundle“), um die noch immer hohe Letalität in der Sepsis zu reduzieren. Indikation, Auswahl und Dauer einer antiinfektiven Therapie bei primären und sekundären Infektionen sollen auch bei kalkuliertem Ansatz beherrscht werden, ebenso wie Prinzipien der Hämodynamik und Herz-Kreislauf-Therapie im septischen Schock.
- **Therapie mit Blutprodukten:** Der Notfallmediziner soll sicher Blutprodukte einsetzen können.
- **Organspendemanagement:** Er muss einen potenziellen Organspender erkennen, über die theoretischen Kenntnisse für einen irreversiblen Hirnfunktionsausfall verfügen und gemeinsam mit dem interdisziplinären Team die Indikation zur organerhaltenden Therapie stellen können (siehe auch Tab. 4, Top 4.13).
- **Notfalltherapie am Lebensende:** Die Gesundheitssysteme in Ländern mit hohem Einkommen sind mit einer wachsenden Zahl von älteren Patienten mit zunehmenden Komorbiditäten und der steigenden Nachfrage nach technologisch fortschrittlicher Versorgung konfrontiert. Die Hälfe aller Sterbefälle in Deutschland ereignet sich im Krankenhaus und nicht selten in der Notaufnahme. Die Weichen für die Aufnahme auf eine Beobachtungsstation oder eine Intensivstation werden oft in der Notaufnahme gestellt. Somit stellt sich die Frage nach einer angemessenen Inanspruchnahme einer Akuttherapie am Lebensende. Für eine qualitativ hochwertige Versorgung am Lebensende („*end-of-life care*“) sind Kompetenz in der Entscheidungsfindung, kommunikative Fähigkeiten sowie die Zusammenarbeit eines gut funktionierenden interdisziplinären Teams erforderlich [46]. Dabei nehmen die ärztliche Indikation und der Patientenwille eine zentrale Rolle in einem komplexen multiprofessionellen und interdisziplinären Entscheidungsprozess ein. Eine der Kernaufgaben von Ärztinnen und Ärzten der Notaufnahme ist in der Beachtung und der Umsetzung ethischer Grundprinzipien zu sehen [47]. Begleitung, Unterstützung und Führung der Angehörigen schwerstkranker Patienten ist ebenfalls Kernelement ärztlicher Prozesse im medizinischen Alltag.

#### 11.1.6. Allgemeiner Teil – Hygienemaßnahmen (Tab. 6)

**Table 6:** 

*6.1*	*Allgemeine Maßnahmen*
WZ 1	Kenntnis grundsätzlicher Hygienemaßnahmen, spezieller Infektionen, des Umgangs mit resistenten Erregern sowie Erkennen der Relevanz von Hygienemaßnahmen
TK 1	Grundsätzlich mögliche Übertragungswege von Keimen; Kontaktübertragung; Tröpfcheninfektion und Airborne-Infektionen
TK 2	Nosokomiale Keime, deren Reservoirs und Pathogenitätsfaktoren sowie die damit verbundenen Übertragungswege
TK 3	Saisonale und ganzjährig vorkommende Viruserkrankungen (insbesondere Schweres-akutes-Atemwegssyndrom-Coronavirus 2 [„severe acute respiratory syndrome coronavirus 2“, SARS-CoV‑2], Influenza, RSV), deren Infektiosität bzw. Kontagiosität, die jeweiligen Übertragungswege und Schutzmaßnahmen
TK 4	Typische Erreger von Infektionen in der Notaufnahme und deren Übertragungswege
TK 5	Grundlagen mikrobiologischer Diagnostik (v. a. Präanalytik, Resistenztestung, Befundinterpretation)
TK 6	Grundkenntnisse des Infektionsschutzgesetzes – insbesondere zum Thema Meldepflicht von Infektionskrankheiten wie Tuberkulose, Influenza und COVID-19 (Coronaviruskrankheit 2019), bakterielle Meningitis
TK 7	Hausinterne Hygienestandards
TK 8	Hausinterne Keim- und Resistenzspektren
PF 1	*Level III: Händedesinfektion
PF 2	*Level III: steriles Einkleiden
PF 3	*Level III: steriles Arbeiten bei der Anlage invasiver Zugänge
PF 4	*Level III: Vorbereitung eines „sterilen Tischs“ bzw. Aufsicht darüber
BV 1	Enge Zusammenarbeit mit den Hygienefachkräften und dem ABS-Team
BV 2	Qualifizierung zur Vermittlung der Standards notwendiger hygienischer Maßnahmen an das Klinikpersonal
BV 3	Qualifizierung zur Vermittlung der Standards notwendiger hygienischer Maßnahmen an das Patientenumfeld
BV 4	Beratung durch Hygienekompetenzzentren bei speziellen/regional ungewöhnlichen Keimen
*6.2*	*Prävention*
WZ 2	Kenntnis grundsätzlicher Präventivmaßnahmen, insbesondere Vermeidung nosokomialer Infektionen sowie Infektionsprävention
TK 9	Allgemeine Hygienemaßnahmen und Standardmaßnahmen zur Infektionsprävention – insbesondere Vermeidung von Wundinfektionen, Katheter- oder Device-Infektionen
TK 10	Allgemeine Hygienemaßnahmen beim Umgang mit infektiösen Patienten inkl. Schutzmaßnahmen, insbesondere bei Infektionskrankheiten mit Tröpfcheninfektion (Tuberkulose, Meningokokken, Viruserkrankungen)
TK 11	Allgemeine Hygienemaßnahmen beim Umgang mit schwer immunsupprimierten Patienten, inklusive „Umkehrschutzmaßnahmen“
TK 12	Fundierte Kenntnisse im Umgang (Schutz und Isolationsmaßnahmen) mit antibiotikaresistenten Erregern (methicillinresistenter *Staphylococcus aureu*s [MRSA], vancomycinresistente Enterokokken [VRE], multiresistente gramnegative Bakterien [MRGN] und Clostridioides difficile)
PF 5	*Level III: adäquater Einsatz persönlicher Schutzausrüstung
BV 5	Vermögen, die Sinnhaftigkeit und Notwendigkeit von Präventivmaßnahmen an das Notaufnahmeumfeld weiterzugeben
BV 6	Sensibilisierung bezüglich des gehäuften Auftretens von Problemkeimen
*6.3*	*Isolierung*
WZ 3	Kenntnis grundsätzlicher Isolationsmaßnahmen und erregerspezifischer Indikationen bei resistenten Erregern, Virusinfektionen – insbesondere Influenza und SARS-CoV-2 – aber auch hochkontagiösen Erkrankungen (z. B. Ebola)
TK13	Isolationsmöglichkeiten, Möglichkeiten und Beschränkungen von Kohortenisolation, Umkehrisolation, Maßnahmen räumlicher Trennung und Voraussetzungen für eine Quarantäne
PF 6	*Level II: sinnvolle Risiko-Nutzen-Abwägung bezüglich hygienischer Präventivmaßnahmen und klinisch notwendigem Monitoring
BV 7	Enge Zusammenarbeit mit dem zuständigen Gesundheitsamt
*6.4*	*Desinfektion*
WZ 4	Kenntnisse gängiger Reinigungs‑, Desinfektions- und Sterilisationsverfahren (chemisch/physikalisch)
TK 14	Chemische Desinfektion (Wirkstoffe/Einwirkzeiten/Resistenzen)
TK 15	Physikalische Desinfektion (thermisch/Bestrahlung/mechanisch)
TK 16	Kennen des hausinternen Desinfektionsplans
*6.5*	*Bauliche Voraussetzungen*
WZ 5	Kenntnisse baulicher Maßnahmen, die eine höhere Sicherheit für die Hygiene ermöglichen
TK 17	Lüftungsanlagen (Luftfilter[stufen]/Zuluft/Abluft) und Unterdruckzimmer sowie Hygiene-Pitfalls (Funktionsstörungen)
TK 18	Trennung aseptischer/unreiner Bereiche
TK 19	Nutzung von Schleusen und deren Indikationen

Anmerkung: Level-I- bis -III-Angaben sind zur Hervorhebung mit einem * versehen

##### Grundlagen und Standards.

Auch Selbstschutz! In Zeiten zunehmender ambulanter und nosokomialer Infektionen und der steigenden Zahl resistenter Erreger rückt die Bedeutung der Hygiene im Arbeitsalltag eines Mediziners, insbesondere aber eines Notfallmediziners, zunehmend in den Vordergrund. Der hygienische Selbstschutz ist ebenso wichtig wie das unbedingte Vermeiden einer Übertragung potenziell tödlicher Keime.

##### Das soll gewusst und gekonnt werden.

- Der Akut- und Notfallmediziner soll sich nicht nur theoretisches Wissen und praktische Fähigkeiten, wie keimarmes oder steriles Arbeiten, aneignen, sondern auch die Fähigkeit besitzen, die Aufmerksamkeit eines jeden Mitarbeiters zu schärfen und seine Patienten und deren Angehörige zu schulen.
- Das Wissen um resistente Erreger, Isolationsmaßnahmen und grundsätzliche Hygienemaßnahmen kann die Prognose der Patienten entscheidend verbessern. Dabei ist eine gute interdisziplinäre Zusammenarbeit mit den Gesundheitsämtern und Krankenhaushygienikern unerlässlich.

#### 11.1.7. Allgemeiner Teil – Pharmakotherapie (Tab. 7)

**Table 7:** 

WZ 1	Sichere und wirksame Arzneimittelbehandlung unter Berücksichtigung der Besonderheiten von Pharmakokinetik und Pharmakodynamik bei Notfallpatienten
*7.1*	*Allgemeine Prinzipien der Pharmakotherapie bei Notfallpatienten*
WZ 2	Verständnis allgemeiner Pharmakodynamik und Pharmakokinetik (Verteilungsvolumina, Proteinbindung und Ausscheidungskinetiken) und deren Veränderungen bei (kritisch) kranken Patienten
TK 1	Standarddosierungen notfallmedizinischer Pharmaka; Ernährungssondenapplikation, periphere und zentrale Venenkatheter
TK 2	Nebenwirkungsprofile notfallmedizinischer Pharmaka: Nieren‑, Leber‑, Neuro- und Knochenmarktoxizität
TK 3	Spezifische Pharmakologie von Inotropika, vasoaktiven Substanzen und Inodilatatoren
TK 4	Interaktionen notfallmedizinischer Pharmaka, Metabolisierungsinteraktionen
TK 5	Spezifische Probleme der Medikamentenapplikation: chemische und physikalische Interaktionen in Sonden und Kathetern
*7.2*	*Antiinfektive Therapie (siehe auch* Tab. 5*, Top 5.12 und *Tab. 13*)*
WZ 3	Antiinfektive Therapie zur Infektionsprophylaxe sowie zur Infektions- und Sepsistherapie auf der Notaufnahme
TK 6	Kalkulierte Primärtherapie, Pharmakokinetik spezifischer Antiinfektiva (Unterschiede konzentrations- und zeitabhängiger Antibiotika, Gewebegängigkeit), bakterizide vs. bakteriostatische Antibiotika
TK 7	Antibiotikatherapie bei Multiresistenzen
TK 8	Antibiotikaprophylaxen vor operativen Eingriffen
TK 9	Einfluss der kritischen Erkrankung auf Plasmaspiegel und Gewebegängigkeit der eingesetzten Antiinfektiva; Bedeutung von Dosisanpassungen, Dosierungsintervallen, prolongierten Infusionslaufzeiten unter Berücksichtigung der ggf. gestörten Organfunktionen (Niere, Leber) und ggf. Antibiotikaspiegelbestimmungen
TK 10	Aktuelle Leitlinien der AWMF, internationale Leitlinien, Empfehlungen des Robert Koch-Instituts und der nationalen Fachgesellschaften zur antiinfektiven Therapie, insbesondere der Sepsis und des septischen Schocks, der Blutstrominfektion, der Pneumonie, der Peritonitis, der Harnwegsinfektion, der Weichteil- und Gewebsinfektion sowie der Endokarditis
PF 1	*Level III: Umsetzung der TK 9 und TK 10 in eine leitlinienorientierte antiinfektive Therapie des kritisch kranken Notfallpatienten
BV1	Mitarbeit im Antibiotic-Stewardship(ABS)-Team [51]; Zusammenarbeit mit der Krankenhaushygiene
*7.3*	*Individualisierte Pharmakotherapie bei bestimmten Notfallpatientengruppen*
WZ 4	Therapieanpassung an spezielle pharmakokinetische und pharmakodynamische Aspekte bei bestimmten Notfallpatientengruppen
TK 11	Therapieanpassung bei akutem Nierenversagen (AKI) und eingeschränkter Nierenfunktion: Modifikation der Dosierungshöhe und der Dosierungsintervalle (Dettli-Formeln)
TK 12	Therapieanpassung bei Adipositas sowie bei gesteigerter renaler Clearance bei hyperdynamischen Kreislaufverhältnissen
TK 13	Therapieanpassung bei Leberversagen sowie bei multiplem Organdysfunktionssyndrom (MODS)/Multiorganversagen
TK 14	Therapieanpassung bei generalisiertem Ödemen sowie bei Hypoproteinämie
TK 15	Therapieanpassung bei geriatrischen Patienten
TK 16	Therapieanpassung bei schwangeren Patientinnen
TK 17	Therapieanpassung bei Medikamenten- und Drogenabhängigkeit
PF 2	*Level III: Umsetzung der in TK 11 bis TK 17 genannten Kenntnisse in eine individualisierte Pharmakotherapie von Notfallpatienten unter Berücksichtigung der pharmakokinetischen Unterschiede bei intermittierender und kontinuierlicher Medikamentenapplikation

Anmerkung: Level-I- bis -III-Angaben sind zur Hervorhebung mit einem * versehen

##### Grundlagen und Standards.

Die gestörten Organfunktionen des Notfallpatienten können sowohl Pharmakokinetik als auch Pharmakodynamik erheblich beeinträchtigen. Auch die Pharmakotherapie in der Schwangerschaft und im höheren Alter ist mit zu berücksichtigen.

##### Das soll gewusst und gekonnt werden.

- **Pharmakokinetik und Pharmakodynamik:** Der Akut- und Notfallmediziner soll bei seinen Patienten auf Besonderheiten bei der Pharmakotherapie gefasst sein und eine sichere und wirksame Arzneimittelbehandlung unter Berücksichtigung der Besonderheiten von Pharmakokinetik und Pharmakodynamik durchführen können. Hierzu gehört auch das Verständnis von Verteilungsvolumina, Proteinbindung und Eliminationskinetiken sowie deren Veränderungen besonders bei kritisch kranken Patienten. Weiterhin muss er Standarddosierungen, Applikationsformen, Nebenwirkungsprofile, Interaktionen und Toxizitäten notfallmedizinischer Pharmaka kennen.
- **Antiinfektiva:** Besonders wichtig in der Notaufnahme sind profunde Kenntnisse der Antibiotikatherapie zur Infektionsprophylaxe sowie zur Infektions- und insbesondere Sepsistherapie. Eine empirische, oft kalkulierte Primärtherapie soll selbstständig indiziert werden können. Von großer Bedeutung ist die Pharmakokinetik spezifischer Antibiotika, konzentrations- und zeitabhängiger Antibiotika und der Antibiotika mit unterschiedlicher Gewebegängigkeit. Ebenso wichtig sind Kenntnisse zu Dosisanpassungen, Dosierungsintervallen und prolongierten Laufzeiten. Probleme der Antibiotikatherapie bei Multiresistenzen, Antibiotikaprophylaxen vor operativen Eingriffen und der Einfluss der kritischen Erkrankung auf Plasmaspiegel und Gewebegängigkeit sollen von notfallmedizinischer Seite in Zusammenarbeit mit ABS-Programmen [51] adressiert werden können.

### 11.2. Angiologische Aspekte in der Akut- und Notfallmedizin (Tab. 8)

**Table 8:** 

WZ 1	Erwerb der für die Betreuung von Notfallpatienten erforderlichen Kenntnisse und Fähigkeiten aus dem Bereich der Gefäßmedizin
*8/1*	*Thromboembolische Erkrankungen: tiefe Venenthrombose (TVT) und Lungenarterienembol*i*e (LAE)*
TK 1	TVT und deren Folgeerkrankungen, insbesondere der LAE und paradoxer Embolien
PF 1	*Level III: Fähigkeit zur selbstständigen Indikationsstellung zur zielführenden Diagnostik sowie der Durchführung der medikamentösen Akuttherapie bei und nach TVT
PF 2	*Level II: Indikationsstellung zum Einsatz interventioneller bzw. operativer Therapieverfahren bei massiver TVT sowie zentraler Lungenarterienembolie und praktische Erfahrung im Einsatz interventioneller Verfahren (lokale Lysetherapie, EKOS^TM^ [Boston Scientific, Marlborough, MA, USA] endovaskuläres System, Thrombektomie)
BV 1	Enge Zusammenarbeit mit prähospitalem Notfallmediziner, Angiologen, Kardiologen und Radiologen zur Früherkennung von TVT und LAE
BV 2	Förderung der Adhärenz bei der Antikoagulanzienbehandlung
*8/2*	*Akute und kritische Extremitätenischämie („acute limb ischaemia“ [ALI]/„critical limb ischaemia“ [CLI])*
TK 2	Akute und kritische Extremitatenischämien und deren Folgeerkrankungen
PF 3	*Level III: Fähigkeit zur selbständigen klinischen Diagnosestellung einer akuten Extremitätenischämie sowie zur Durchführung der medikamentösen Akuttherapie; Indikationsstellung zur zielführenden Diagnostik sowie Durchführung der medikamentösen Akut- und Langzeittherapie bei kritischer Beinischämie einschließlich der Durchführung primär- und sekundärpräventiver Maßnahmen
PF 4	*Level II: Indikationsstellung zum Einsatz interventioneller bzw. operativer Therapieverfahren
BV 3	Zusammenarbeit mit prähospitalem Notfallmediziner, Angiologen, Gefäßchirurgen und Radiologen zur unmittelbaren Behandlung einer akuten Beinischämie und Früherkennung einer kritischen Beinischämie
*8/3*	*Akute Mesenterialischämie*
TK 3	Akute Mesenterialischämie
PF 5	*Level III: selbständige Indikationsstellung zur zielführenden Diagnostik sowie zur Durchführung der medikamentösen Akuttherapie
PF 6	*Level III: Indikationsstellung zur weiterführenden sonographischen und radiologischen Diagnostik
PF 7	*Level II: Indikationsstellung zum Einsatz interventioneller bzw. operativer Therapieverfahren
BV 4	Zusammenarbeit mit prähospitalem Notfallmediziner, Angiologen, Gefäßchirurgen, Gastroenterologen, Viszeralchirurgen und Radiologen zur unmittelbaren Erkennung und Behandlung einer akuten mesenterialen Ischämie
*8/4*	*Akut entzündliche Gefäßerkrankungen (Vaskulitiden) (siehe auch Abschnitt 11.12 inkl.* Tab. 18)
TK 4	Akut entzündliche Gefäßerkrankungen und deren Folgeerkrankungen
PF 8	*Level III: Indikationsstellung zur zielführenden Diagnostik sowie zur Durchführung der medikamentösen Akuttherapie bei und nach einer akut entzündlichen Gefäßerkrankung
BV 5	Zusammenarbeit mit Angiologen, Radiologen, Rheumatologen, Nephrologen, Ophthalmologen zur Früherkennung und Behandlung einer akut entzündlichen Gefäßerkrankung

Anmerkung: Level-I- bis -III-Angaben sind zur Hervorhebung mit einem * versehen

#### Grundlagen und Standards.

Das Weiterbildungsziel ist der Erwerb der für die Betreuung von akut- und notfallmedizinischen Patienten erforderlichen gefäßmedizinischen Kenntnisse.

#### Das soll gewusst und gekonnt werden.

- **Thromboembolische Erkrankungen: ***Tiefe Venenthrombose* (TVT) und *Lungenarterienembolie* (LAE): Diagnostik und Therapie der TVT sowie der LAE erfordern rasches und leitlinienorientiertes Handeln unter Berücksichtigung der Klinik und der sonographischen sowie angiologischen oder radiologischen (CT-)Gefäßdiagnostik, ggf. unter Einbeziehung interventioneller bzw. operativer Therapieverfahren. Erforderlich sind ein adäquates Standardmonitoring sowie die kontinuierliche Überwachung von Patienten mit instabilen Kreislaufverhältnissen (z. B. intermediär hohes Risiko). Zudem müssen seltenere, aber akut lebensbedrohliche Krankheitsbilder, wie die obere Einflussstauung bei Verschluss der V. cava superior oder die Phlegmasia coerulea dolens, erkannt und unmittelbar der Therapie zugeführt werden.
- **Akute und kritische Extremitätenischämie: **Rasches interdisziplinäres Handeln ist bei der kritischen Extremitätenischämie – insbesondere der akuten Arm- oder Beinischämie – erforderlich unter Einbeziehung der konservativ-medikamentösen, interventionellen bzw. operativen Therapieverfahren. Er soll die Dringlichkeit der Therapie anhand klinischer Parameter einschätzen und eine akute Ischämie, die eine unmittelbare Therapie erfordert, von einer kritischen Extremitätenischämie abgrenzen können.
- **Akute Mesenterialischämie: **Die akute mesenteriale Ischämie ist ein in höherem Alter häufiges Krankheitsbild, das Überleben der Patienten hängt entscheidend von der raschen Diagnosestellung und Einleitung gezielter radiologischer (CT-)Diagnostik ab. Die interdisziplinäre Therapieeinleitung ist zu koordinieren.
- **Akut entzündliche Gefäßerkrankungen (Vaskulitiden) (siehe auch Abschnitt 11.12 inkl.** Tab. 18): Differenzialdiagnostisch sind bei schwerkranken Gefäßpatienten auch Vaskulitiden in Betracht zu ziehen. Die frühzeitige Diagnostik und adäquate Therapie, ggf. durch Einbezug von Kollegen mit einer fundierten angiologischen Fachexpertise, sind oft ausschlaggebend für die Vermeidung oder Begrenzung schwerwiegender Organschäden.

### 11.3. Endokrinologische, diabetologische und metabolische Aspekte in der Akut- und Notfallmedizin (Tab. 9)

**Table 9:** 

WZ 1	Erwerb der für die Betreuung von Akut- und Notfallpatienten erforderlichen Kenntnisse der Klinik, Diagnostik und Therapie relevanter endokriner, diabetologischer und metabolischer Störungen
*9.1*	*Primäre Hypophysenfunktionsstörungen und hypophysäre Funktionsstörungen beim kritisch kranken Patienten*
TK 1	Basiskenntnisse der Physiologie und Pathophysiologie des Energiemetabolismus, zirkadianer Oszillatoren und neuroendokriner Hormonachsen
TK 2	Pathophysiologie, Klinik, Diagnostik und Behandlung → Hypothalamisch-hypophysäre Störungen inkl. Diabetes insipidus centralis, Abgrenzung zu Diabetes insipidus renalis → (Pan‑)Hypopituitarismus inkl. hypophysäres Koma → Insuffizienz einzelner Hypophysenachsen, insbesondere der kortikotropen Achse → „Non-thyroidal-illness(low-T3)“-Syndrom
PF 1	*Level III: Akutbehandlung des hypophysären Hypokortisolismus
PF 2	*Level III: Akutbehandlung des (Pan‑)Hypopituitarismus
PF 3	*Level II: Akutbehandlung des Diabetes insipidus
PF 4	*Level III: Akutbehandlung des Syndroms der inadäquaten ADH-Sekretion (SIADH; s. auch Hyponatriämie: Tab. 15, 15/4)
PF 5	*Level II (zu TK 2): Erkennen typischer klinischer Symptome und Befundkonstellationen, Auswahl geeigneter weiterführender Labordiagnostik, Vermeiden irreführender diagnostischer und therapeutischer Eskalationen
PF 6	*Level II: Hormonersatztherapie bei vorbestehendem Hypopituitarimus
BV1	Interdisziplinäre Zusammenarbeit mit Endokrinologen, Neurochirurgen, Nephrologen und Anästhesisten
*9.2*	*Nebennierenerkrankungen*
TK 3	Ursachen, Klinik und Diagnostik der primären und sekundären Nebennierenrinden(NNR)-Insuffizienz inkl. der Addison-Krise und des Mineralokortikoidmangels
TK 4	Klinische Präsentation, Diagnostik und Initialbehandlung des Hyperkortisolismus
TK 5	Leitlinienorientierte Hydrokortisongabe in der adjunktiven Therapie des septischen Schocks; Dosiseskalation bei akuter oder vorbestehender Nebennierenrindeninsuffizienz
TK 6	Pharmakologische Interaktionen, z. B. Nebennierenrinde und Etomidat
TK 7	Klinik und Diagnostik des Phäochromozytoms
TK 8	Diagnostik und Initialbehandlung endokriner hypertensiver Notfälle
PF 7	*Level III: Initialdiagnostik und -therapie der Addison-Krise und der mineralokortikoiden Insuffizienz
PF 8	*Level III: Akutbehandlung endokriner hypertensiver Notfälle
PF 9	*Level II: spezifische Akutbehandlung bei Phäochromozytom
BV 2	Interdisziplinäre Zusammenarbeit mit Endokrinologen, endokrinen Chirurgen, Anästhesisten und Nephrologen
*9.3*	*Schilddrüsenerkrankungen*
TK 9	Pathophysiologie, Klinik, Diagnostik und Akutbehandlung → Hyperthyreose/thyreotoxische Krise → Hypothyreose/Myxödemkrise → Amiodaronassoziierte Schilddrüsenstörungen
TK 10	Indikation und Durchführung prophylaktischer Maßnahmen vor Applikation jodhaltiger Röntgenkontrastmittel bei Schilddrüsenstörungen
PF 10	*Level III: Anamnese und klinische Untersuchung bei Verdacht auf Schilddrüsenfunktionsstörungen
PF 11	*Level III: Erkennen typischer laborchemischer Befundkonstellationen bei Schilddrüsenerkrankungen
PF 12	*Level III: Fähigkeit zur selbstständigen Indikationsstellung einer zielführenden Diagnostik und Durchführung der medikamentösen Akuttherapie
PF 13	*Level II: Basiserfahrung mit der Schilddrüsensonographie inkl. Duplex
PF 14	*Level II: Kenntnisse von (therapieassoziierten) Komplikationen
BV 3	Interdisziplinäre Zusammenarbeit mit Endokrinologen und endokrinen Chirurgen
*9.4*	*Diabetes mellitus*
WZ 2	Erwerb der für die Betreuung von akut- und notfallmedizinischen Patienten erforderlichen Kenntnisse der Klinik, Diagnostik und Therapie relevanter Störungen der Glukosehomöostase
TK 11	Ursachen, klinische Zeichen und Behandlung einer Hypoglykämie
TK 12	Pathophysiologie, Klinik und Behandlung der diabetischen Ketoazidose (DKA) inkl. der euglykämischen DKA unter Natrium-Glukose-Cotransporter(SGLT)-2-Inhibitoren
TK 13	Pathophysiologie, Klinik und Behandlung einer hyperosmolaren hyperglykämischen Entgleisung sowie assoziierter Komplikationen
TK 14	Ursachen, klinische und prognostische Bedeutung sowie Behandlung einer sog. Stresshyperglykämie
PF 15	*Level III: Erkennen typischer klinischer Symptome und Befundkonstellationen bei Hypo- und Hyperglykämien
PF 16	*Level III: Anwendung differenzierter Therapiealgorithmen in der Akutphase
BV 4	Interdisziplinäre Zusammenarbeit mit der Diabetologie
*9.5*	*Adipositas*
WZ 3	Erwerb der für die Betreuung von akut- und notfallmedizinischen Patienten erforderlichen Kenntnisse bei schwerer Adipositas und nach bariatrischen Operationen
TK 15	Kenntnisse zu den Besonderheiten bei der Betreuung von Akut- und Notfallpatienten bei schwerer Adipositas und nach bariatrischen Operationen
*9.6*	*Metabolische Störungen*
WZ 4	Erwerb der für die Betreuung akut- und notfallmedizinischer Patienten erforderlichen Kenntnisse der Klinik, Diagnostik und Therapie notfallmedizinisch relevanter metabolischer Störungen
TK 16	Pathophysiologie, Klinik, Diagnostik und Akutbehandlung: → Gichtanfall → Metforminassoziierte Laktatazidose → Thiaminmangel und Refeeding-Syndrom → Hyperammonämien
PF 17	*Level II: Indikation zu weiterführender gezielter Diagnostik und Notfalltherapie bei zeitkritischen seltenen Stoffwechselstörungen (s. TK 16)
BV 5	„Awareness“ für die rationale Diagnostik seltener Stoffwechselstörungen
BV 6	„Awareness“ für patientenindividuelles Vorgehen bei vorbestehenden seltenen Stoffwechselstörungen
BV 7	Interdisziplinäre Zusammenarbeit mit Stoffwechselexperten, Endokrinologen und Nephrologen sowie ggf. Adhärenz zur spezifischen (Online‑)Literaturrecherche

Anmerkung: Level-I- bis -III-Angaben sind zur Hervorhebung mit einem * versehen

#### Grundlagen und Standards.

Das Weiterbildungsziel ist der Erwerb der für die Betreuung von akut- und notfallmedizinischen Patienten erforderlichen Kenntnisse der Klinik, Diagnostik und Therapie relevanter endokriner, diabetologischer und metabolischer Störungen.

#### Das soll gewusst und gekonnt werden.

- Zu erkennen sind Regulationsstörungen integrativer neuroendokriner Signalpfade beim kritisch kranken Patienten, wie Diabetes insipidus, hypophysäres Koma, Insuffizienz adenohypophysärer Achsen, insbesondere der kortikotropen Achse, und das Non-thyroidal-illness-Syndrom; zum anderen Nebennierenerkrankungen wie die Addison-Krise, mineralokortikoide Insuffizienz (auch z. B. bei adrenogenitalem Syndrom) und Phäochromozytom. Wichtig ist auch die leitlinienorientierte Hydrokortisonsubstitution bei absoluter oder relativer Nebenniereninsuffizienz, z. B. bei Patienten mit septischem Schock oder bei diagnostischen oder operativen Eingriffen.
- Weiterhin von Relevanz sind Schilddrüsenerkrankungen, wie thyreotoxische und Myxödemkrise, der Diabetes mellitus mit hypoglykämischer sowie hyperosmolarer hyperglykämischer Entgleisung und diabetischer Ketoazidose sowie die Besonderheiten bei schwerer Adipositas und nach bariatrischen Operationen.
- Differenzialdiagnostisch sollte der Akut- und Notfallmediziner auch an seltene, aber gravierende Stoffwechselstörungen denken, wie beispielsweise Gicht, metforminassoziierte Laktatazidose, arzneimittelbedingte Ketoazidose oder auch akute hepatische Porphyrien.

### 11.4. Gastroenterologische Aspekte in der Akut- und Notfallmedizin (Tab. 10)

**Table 10:** 

WZ 1	Erwerb der für die Betreuung von Akut- und Notfallpatienten erforderlichen Kenntnisse der Klinik, Diagnostik und Therapie relevanter gastroenterologischer Störungen [52] sowie der Pharmakotherapie bei gastrointestinalen Notfällen [53]
*10.1*	*Gastrointestinale Blutungen*
WZ 2	Fähigkeit zur Diagnose und nichtinterventionellen Therapie von gastrointestinalen (GI-)Blutungen
TK 1	Differenzierung von oberer vs. unterer GI-Blutung: Hämatemesis, Teerstuhl, Hämatochezie
TK 2	Ursachen der oberen GI-Blutung
TK 3	Differenzierung akuter variköser und nichtvariköser Blutungen
TK 4	Ursachen der unteren GI-Blutung
TK 5	Initiale Risikostratifizierung
TK 6	Initiale pharmakologische Maßnahmen: Protonenpumpeninhibitoren (PPI), selektive Vasopressoren [53]
TK 7	Kreislaufstabilisierung, Volumenmanagement
TK 8	Blutprodukte- und Gerinnungsmanagement
TK 9	Optionen der endoskopischen Blutstillung
TK 10	Sekundäre bzw. alternative Optionen der Blutungsstillung (interventionelle Radiologie, chirurgische Intervention)
TK 11	Vorbereitung zur Endoskopie: PPI, Prokinetika (z. B. Erythromycin), Darmreinigung
TK 12	Periinterventionelle Versorgung bei endoskopischen Eingriffen
TK 13	Klinische Abschätzung des Rezidivblutungsrisikos
PF 1	*Level III: Risikostratifizierung, Kreislauftherapie, differenziertes Gerinnungsmanagement und Vorbereitung zur Endoskopie
*10.2*	*Akute und chronische Lebererkrankungen*
WZ 3	Fähigkeit zur Diagnose und Akutbehandlung von akuten und chronischen Lebererkrankungen
TK 14	Klinische Einordnung erhöhter Leberwerte (Hepatitis, Cholestase, Synthesestörungen)
TK 15	Akutes Leberversagen (Definition, Diagnostik, Ursachen, spezifische Therapieoptionen, Risikostratifizierung)
TK 16	Paracetamolintoxikation, primäre und sekundäre Giftelimination, N‑Acetylcystein-Behandlung
TK 17	Akut-auf-chronisches Leberversagen (Definition, Diagnostik und Therapie der auslösenden Faktoren: Infektion, Blutung, Medikamente, Alkohol)
TK 18	Komplikationen der dekompensierten Leberzirrhose (Aszites, hepatorenales Syndrom, hepatische Enzephalopathie, Varizenblutung; [54])
PF 2	*Level III: Durchführung der diagnostischen und therapeutischen Parazentese
PF 3	*Level III: Diagnostik und Behandlung der spontan-bakteriellen Peritonitis (SBP)
PF 4	*Level III: Akutbehandlung des hepatorenalen Syndroms
PF 5	*Level III: Akutbehandlung der hepatischen Enzephalopathie
PF 6	*Level III: nichtinterventionelles Management der varikösen Blutung, selektive Vasopressoren, Prophylaxe der SBP, Atemwegsmanagement, Volumentherapie
PF 7	*Level III: differenziertes Blutprodukte- und Blutgerinnungsmanagement
PF 8	*Level III: Notfallsonographie: Schwerpunkt Leber und Lebergefäße
BV 1	Interdisziplinäre Zusammenarbeit, Schwerpunkt Gastroenterologie und Nephrologie
*10.3*	*Erkrankungen der Gallenblase und Gallenwege*
WZ 4	Fähigkeit zur Diagnose und Therapie von Erkrankungen der Gallenwege
TK 19	Differenzialdiagnostik rechtseitiger Oberbauchschmerz
TK 20	Differenzialdiagnostik prä-/intra-/posthepatischer Ikterus
TK 21	Indikation und Timing der Cholezystektomie bei akuter Cholezystitis
TK 22	Indikation und Timing der Notfall-ERC(P)
PF 9	*Level III: (infektiologisches) Management der (akuten) Cholezystitis, Cholangitis
PF 10	*Level II: Notfallsonographie: Schwerpunkt Leber, Gallenblase, Gallenwege
BV 2	Interdisziplinäre Zusammenarbeit, Schwerpunkt Gastroenterologie und Viszeralchirurgie
*10.4*	*Pankreatitis*
WZ 5	Fähigkeit zur Diagnose und Akuttherapie der akuten Pankreatitis und des akuten Schubs einer chronischen Pankreatitis
TK 23	Diagnosekriterien und Ursachen der akuten Pankreatitis
TK 24	Verlaufsformen der akuten Pankreatitis, Risikostratifizierung, Komplikationen
TK 25	Differenzierte Volumentherapie, Schmerztherapie
TK 26	Indikationen zur CT-Diagnostik (diagnostisch vs. prognostisch)
TK 27	Diagnostik und Therapie der biliären Pankreatitis (Labor, Bildgebung, ERC[P])
PF 11 PF 12 PF 13 PF 14 PF 15	*Level III: Volumentherapie und Monitoring *Level III: Schmerztherapie *Level III: Pleura‑/Aszitespunktion *Level III: Notfallsonographie: Schwerpunkt Pankreas, Gallenwege, Gallenblase *Level III: Messung und Interpretation des intraabdominellen Drucks
BV 3	Interdisziplinäre Zusammenarbeit, Schwerpunkt Gastroenterologie
*10.5*	*Erkrankungen des Magen-Darm-Trakts*
WZ 6	Fähigkeit zur Diagnose und Akuttherapie von Erkrankungen des Magen-Darm-Trakts
TK 28	Differenzialdiagnostik Oberbauchschmerz
TK 29	Differenzialdiagnostik des Erbrechens
TK 30	Indikationen zur endoskopischen Bolus‑/Fremdkörperextraktion
TK 31	Mechanischer und paralytischer Ileus
TK 32	Gastrointestinale Ischämie
TK 33	Divertikulitis
TK 34	Akuter Schub chronisch-entzündlicher Darmerkrankungen
TK 35	Hohlorganperforation, Peritonitis
TK 36	Indikationen zur computertomographischen Bildgebung
PF 16	*Level I: Darmsonographie
PF 17	*Level III: Notfallsonographie des Abdomens
PF 18	*Level II: Anwendung differenzierter Diagnose- und Therapiealgorithmen bei infektiösen Durchfallerkrankungen
PF 19	*Level II: Anwendung differenzierter Diagnose- und Therapiealgorithmen bei gastrointestinaler Ischämie
PF 20	*Level II: Anwendung differenzierter Diagnose- und Therapiealgorithmen bei Divertikulitis
PF 21	*Level II: Anwendung Diagnose- und Therapiealgorithmen bei einem akuten Schub chronisch-entzündlicher Darmerkrankungen
BV 4	Interdisziplinäre Zusammenarbeit, Schwerpunkt Gastroenterologie, Viszeralchirurgie, Radiologie

Anmerkung: Level-I- bis -III-Angaben sind zur Hervorhebung mit einem * versehen

#### Grundlagen und Standards.

Das Weiterbildungsziel ist der Erwerb der für die Betreuung von akut- und notfallmedizinischen Patienten erforderlichen Kenntnisse der Klinik, Diagnostik und Therapie relevanter gastroenterologischer Störungen [52].

#### Das soll gewusst und gekonnt werden.

- **Gastrointestinale Blutungen: **Fähigkeit zur Diagnose und nichtinterventionellen Therapie von gastrointestinalen (GI-)Blutungen [28]: Differenzierung zwischen oberer und unterer GI-Blutung und Kenntnis der verschiedenen Ursachen gastrointestinaler Blutungen. Die Unterschiede in der Risikobewertung und im Management von akuten varikösen und nichtvarikösen Blutungen sind dem Akut- und Notfallmediziner vertraut. Er beherrscht die initiale pharmakologische Behandlung bei akuten GI-Blutungen [53], die Kreislaufstabilisierung und das differenzierte Volumen- sowie das Blutprodukte- und Gerinnungsmanagement. Das Wissen um die Möglichkeiten der endoskopischen Blutungsstillung sowie der interventionellen Radiologie und der chirurgischen Intervention ist ein wichtiger Bestandteil der Tätigkeit. Er kennt die Maßnahmen zur Vorbereitung zur Endoskopie und der periinterventionellen Betreuung bei endoskopischen Eingriffen. Die interdisziplinäre Zusammenarbeit ist eng und umfasst die Schwerpunkte Gastroenterologie, interventionelle Radiologie und Viszeralchirurgie.
- **Akute und chronische Lebererkrankungen: **Erforderlich ist die Fähigkeit zur Diagnose und Therapie von akuten und chronischen Lebererkrankungen. Von besonderer Bedeutung sind die korrekte klinische Einordnung erhöhter Leberwerte sowie Kenntnisse über Definition, Diagnostik, Ursachen, spezifische Therapieoptionen und Risikostratifizierung des akuten und akut-auf-chronischen Leberversagens. Weitere wichtige Bestandteile der Tätigkeit sind die Diagnostik und Therapie der Komplikationen der dekompensierten Leberzirrhose, insbesondere von Aszites, spontan-bakterieller Peritonitis, hepatorenalem Syndrom, hepatischer Enzephalopathie, und das nichtinterventionelle Management der varikösen Blutung [54, 55]. Fundierte Kenntnisse der Schockbehandlung und des differenzierten Blutprodukte- und Blutgerinnungsmanagement sind notwendig. Die Abdomensonographie mit den Schwerpunkten Leber und Lebergefäße soll beherrscht werden. Die interdisziplinäre Zusammenarbeit erfolgt insbesondere mit der Gastroenterologie.
- **Erkrankungen der Gallenwege: **Erforderlich ist die Fähigkeit zur Diagnose und Therapie von Erkrankungen der Gallenwege. Der Akut- und Notfallmediziner beherrscht die Differenzialdiagnostik des rechtseitigen Oberbauchschmerzes und des prä-/intra-/posthepatischen Ikterus. Die Behandlung der akuten Cholezystitis und Cholangitis als Erkrankungen der Gallenwege steht in der Notaufnahmestation im Vordergrund. Eine enge interdisziplinäre Zusammenarbeit mit der Gastroenterologie und der Viszeralchirurgie ist unabdingbar. Das Wissen um die Indikationsstellung und das richtige Timing von Cholezystektomie und notfallmäßiger endoskopischer retrograder Cholangiographie (ERC) ist notwendig. Die Abdomensonographie mit den Schwerpunkten Leber, Gallenblase, Gallenwege soll beherrscht werden.
- **Pankreatitis: **Der Akut- und Notfallmediziner kennt die Prinzipien der Diagnostik und Therapie der akuten Pankreatitis und des akuten Schubs einer chronischen Pankreatitis. Er ist vertraut mit den Diagnosekriterien und Ursachen der akuten Pankreatitis und kennt die Verlaufsformen und Komplikationen der akuten Pankreatitis. Er beherrscht die differenzierte Volumentherapie bei akuter Pankreatitis und kennt Maßnahmen zur Beurteilung des intravasalen Volumens. Er weiß um die Prinzipien der Schmerztherapie bei akuter Pankreatitis. Wichtig ist das Wissen um die Indikationen zur CT-Diagnostik bei akuter Pankreatitis im Hinblick auf diagnostische bzw. prognostische Fragestellungen. Er hat profunde Kenntnisse der Abdomensonographie mit den Schwerpunkten Pankreas, Gallenwege, Gallenblase und beherrscht die Messung und Interpretation des intraabdominellen Drucks. Die interdisziplinäre Zusammenarbeit erfolgt schwerpunktmäßig mit der Gastroenterologie.
- **Erkrankungen des Magen-Darm-Trakts: **Der Akut- und Notfallmediziner soll die Fähigkeit zur Diagnose und Therapie von Erkrankungen des Magen-Darm-Trakts besitzen. Im Vordergrund steht die differenzialdiagnostische klinische Beurteilung abdomineller Schmerzen sowie von Erbrechen und Diarrhö. Er hat profunde Kenntnisse von Diagnostik und Therapie des mechanischen und paralytischen Ileus, der gastrointestinalen Ischämie, der Divertikulitis, des akuten Schubs chronisch-entzündlicher Darmerkrankungen und von Hohlorganperforationen und konsekutiver Peritonitis. Eine enge interdisziplinäre Zusammenarbeit mit den Fachbereichen Gastroenterologie, Viszeralchirurgie und Radiologie ist notwendig. Er ist mit den Indikationen und Verfahren der radiologischen Abdominaldiagnostik vertraut und beherrscht die Abdomensonographie. Er kennt die Indikationen zur endoskopischen Bolus- bzw. Fremdkörperextraktion und ist mit der periinterventionellen Betreuung bei endoskopischen Eingriffen vertraut.

### 11.5. Geriatrische Aspekte in der Akut- und Notfallmedizin (Tab. 11)

**Table 11:** 

WZ 1	Vermittlung von alters- und geriatriespezifischen Aspekten der klinischen Akut- und Notfallmedizin [63–65]
TK 1	Grundbegriffe der Geriatrie, wie Frailty, Sarkopenie, Multimorbidität [58, 66–68], und Definition des geriatrischen Patienten [69]
TK 2	Akutmedizinisch relevante altersspezifische Einschränkungen einzelner Organfunktionen
TK 3	Geriatrische Syndrome
TK 4	Altersspezifische Aspekte der Arzneimitteltherapie, insbesondere Multimedikation, Arzneimittelinteraktionen und Verordnungskaskaden [59, 60]
TK 5	Unspezifische Beschwerden, atypische/fehlende Symptomatik beim geriatrischen Patienten – „non specific complaints“ (NSC; [61, 70])
PF 1	*Level I: Assessment in der Geriatrie (klinische Frailty-Skala, Mini-Cog, 4‑AT-Delirscreening [71])
PF 2	*Level II: praktische Umsetzung der Erkenntnisse zu altersspezifischen Aspekten beim geriatrischen Akut- und Notfallpatienten im Hinblick auf Diagnostik und Therapiemaßnahmen
PF 3	*LeveI II: Berücksichtigung altersspezifischer Besonderheiten und Begleiterkrankungen wie deren Auswirkungen auf Diagnostik und Therapie
PF 4	*Level II: Anpassung akutmedizinischer Therapiemaßnahmen an Outcomeabschätzung und Therapieerwartung des geriatrischen Patienten; Bewertung von Funktionalität und Einschätzung der Prognose [72–74]
BV 1	Berücksichtigung der möglicherweise vorhandenen Multimorbidität und Frailty bei geriatrischen Patienten
BV 2	Berücksichtigung möglicher vorhandener kognitiver Störungen bei geriatrischen Patienten
BV 3	Berücksichtigung der möglicherweise vorhandenen sensorischen und funktionellen Einschränkungen bei geriatrischen Patienten
BV 4	Berücksichtigung der besonderen Situation geriatrischer Patienten am Lebensende
BV 5	Einbeziehung geriatrischer und palliativmedizinischer Expertise zur Symptomkontrolle beim geriatrischen Akut- bzw. Notfallpatienten
BV 6	Zusammenarbeit mit Geriatern in der Feststellung des geriatrischen Behandlungsbedarfs und der innerklinischen Disposition

Anmerkung: Level-I- bis -III-Angaben sind zur Hervorhebung mit einem * versehen

#### Grundlagen und Standards.

Der Anteil der über 70-jährigen Notfallpatienten liegt in der Notaufnahme bei 30 % [56]. Insofern ist es für den klinischen Notfallmediziner wichtig, Spezifika dieser Patienten zu kennen [57]. Dazu zählen unter anderem das Verständnis von Grundbegriffen der Geriatrie und Gerontologie, wie Sarkopenie und Frailty [58], altersspezifische Einschränkungen der Organfunktionen, geriatrische Syndrome, kognitive Dysfunktion, Multimorbidität, Polypharmazie und Polypragmasie sowie Arzneimittelinteraktionen und die häufigsten Verordnungskaskaden [59, 60].

#### Das soll gewusst und gekonnt werden.

- **Besonderheiten in Symptomatik und Diagnostik:**
  - Bei geriatrischen Patienten zeigen sich akute Erkrankungen meist durch unspezifische Symptome wie funktionelle oder kognitive Veränderung, Stürze oder Delir. Bis zu 20 % der älteren Patienten geben im Notfall unspezifische Beschwerden an und 51–59 % dieser Älteren mit unspezifischen Beschwerden haben ein akut behandlungsbedürftiges Problem [61].
  - Auch in der **Diagnostik** sind die Besonderheiten beim geriatrischen Patienten zu beachten. Durch die meist vorbestehende Multimorbidität können Akutsymptome überlagert und Befunde fehlinterpretiert werden. Dies kann auch Auswirkungen auf die Beurteilung der Behandlungsdringlichkeit haben [62].
- **Realistische Therapieoptionen, Patientenwünsche und Vorsorgeplanung:** Wesentlich ist es, die machbaren Therapieoptionen in der Akut- und Notfallmedizin mit den Patientenwünschen in Kooperation mit dem Patienten abzugleichen, ggf. auch mit Angehörigen, betreuenden Ärzten und Pflegeeinrichtungen und auch in Absprache mit Geriatern und Palliativmedizinern. Die vorausschauende Vorsorgeplanung („advance care planing“) stellt einen wichtigen Pfeiler der adäquaten Behandlung dar.

### 11.6. Hämatoonkologische Aspekte in der Akut- und Notfallmedizin (Tab. 12)

**Table 12:** 

*12.1*	*Akute Notfälle bei Patienten mit Krebs(neu)erkrankungen*
WZ 1	Fähigkeit zur Identifikation, Diagnostik und Behandlungseinleitung bei akuten Notfällen von Krebs(neu)erkrankungen
TK1	Grundlegende Kenntnisse hämatoonkologischer Erkrankungen und Notfallsituationen
TK2	Einleitung entsprechender diagnostischer Schritte bei hämatoonkologischen Notfällen
TK3	Interpretation pathologischer Veränderungen im Rahmen von Notfällen bei hämatologisch-onkologischen Erkrankungen
TK4	Therapieeinleitung bei hämatoonkologischen Notfallsituationen
PF1	*Level III: Erkennen eines Zusammenhangs von klinischem Befund und Notfallsituationen bei hämatoonkologischen Erkrankungen *Level II: praktische Erfahrungen in der Behandlung von Notfallsituationen bei hämatoonkologischen Erkrankungen *Level I: pathophysiologische Kenntnisse über spezifische Veränderungen im Rahmen einer Notfallsituation bei hämatoonkologischen Erkrankungen
PF2	*Level III: Einleitung einer Basisdiagnostik *Level II: Einleitung einer spezifischen Diagnostik nach Rücksprache mit einem Hämatoonkologen bei der Erstdiagnose von hämatoonkologischen Erkrankungen und Notfallsituationen *Level I: Durchführung von spezifischen diagnostischen Schritten bei Notfallsituationen im Rahmen hämatoonkologischer Erkrankungen
PF3	*Level I: Erkennen eines Zusammenhanges von klinischen Symptomen, Befunden und Ergebnissen der eingeleiteten Basisdiagnostik mit einer Notfallsituation im Rahmen einer hämatoonkologischen Erkrankung *Level II: Einleitung einer konsiliarischen (hämatologischen) Expertise *Level I: Differenzialdiagnostische Überlegungen
PF4	*Level III: Einleitung therapeutischer Basismaßnahmen in Notfallsituationen. *Level II: Einleitung spezifischer therapeutischer Maßnahmen in einer Notfallsituation (Tumorlysesyndrom, Kompressionssyndrome, Elektrolytstörungen, Blutungs- bzw. Gerinnungskomplikationen, neurologische Symptome, neutropenisches Fieber, Sichelzellkrise, Transfusionsreaktionen, obere Einflussstauung, Spinalkompression, Mikroangiopathien, wie thrombotisch-thrombozytopenische Purpura [TTP]/hämolytisch-urämisches Syndrom [HUS], Meningiosis) *Level I: Organisation und Management einer entsprechenden hämatoonkologischen Versorgung nach der initialen Notfallversorgung nach Absprache mit Hämatoonkologen
BV 1	Ergebnisorientiertes Handeln bei Patienten mit einer Krebserkrankung
BV 2	Fähigkeit zur engen Zusammenarbeit mit prähospitalem und Krankenhausnotfallmediziner, Hämatoonkologen, Chirurgen, Neurologen/Neurochirurgen, Strahlentherapeuten, Radiologen und weiteren Fachärzten, die in die Behandlung von Krebspatienten involviert sind
*12.2*	*Therapieassoziierte akute Notfälle bei Krebspatienten*
WZ 2	Fähigkeit zur Identifikation, Diagnostik und Behandlungseinleitung von therapieassoziierten akuten Notfällen bei Krebspatienten
TK 5	Grundlegende Kenntnisse hämatoonkologischer therapieassoziierter Notfallsituationen
TK 6	Einleitung entsprechender diagnostischer Schritte bei therapieassoziierten hämatoonkologischen Notfällen
TK 7	Interpretation pathologischer Veränderungen im Rahmen therapieassoziierter hämatoonkologischer Notfälle
TK 8	Therapieeinleitung bei therapieassoziierten hämatoonkologischen Notfallsituationen
PF 5	*Level III: Einleitung einer Basisdiagnostik *Level II: Einleitung einer spezifischen Diagnostik nach Rücksprache mit einem Hämatoonkologen bei therapieassoziierten Notfallsituationen
PF 6	*Level III: Erkennen eines Zusammenhangs von klinischen Symptomen, Befunden und Ergebnissen der eingeleiteten Basisdiagnostik mit einer therapieassoziierten hämatoonkologischen Notfallsituation *Level II: Einleitung einer konsiliarischen (hämatologischen) Expertise *Level I: Differenzialdiagnostische Überlegungen
PF 7	*Level III: Einleitung therapeutischer Basismaßnahmen in Notfallsituationen *Level II: Einleitung spezifischer therapeutischer Maßnahmen in einer therapieassoziierten Notfallsituation (CAR-T-Zell-Therapie, Tumorlysesyndrom, Elektrolytstörungen, Blutungs- bzw. Gerinnungskomplikationen, immuntherapieassoziierte Nebenwirkungen, stammzelltransplantierte Patienten) *Level I: nach Absprache mit Hämatoonkologen Einleitung einer spezifischen Therapie

Anmerkung: Level-I- bis -III-Angaben sind zur Hervorhebung mit einem * versehen

#### Grundlagen und Standards.

Obwohl es für Deutschland keine Zahlen gibt, zeigt eine internationale Studie [75], dass länderspezifisch 24–42,5 % der Krebsfälle in der Notaufnahme diagnostiziert werden. Zusätzlich stellen sich aber auch viele Patienten mit einer schon bekannten Krebserkrankung in der notfallmedizinischen Versorgung vor, aufgrund von Komplikationen entweder im Rahmen der Krebserkrankung oder therapieassoziiert.

#### Das soll gewusst und gekonnt werden.

- Der Akut- und Notfallmediziner soll nicht nur die typischen Komplikationen von Krebserkrankungen und deren Therapie, sondern auch Krebsneuerkrankungen erkennen und entsprechende diagnostische und therapeutische Schritte in Kooperation mit einem Hämatoonkologen bzw. anderen Fachdisziplinen einleiten können.
- **Psychoonkologie:** Patienten stellen sich entweder mit Komplikationen der Krebserkrankung, deren Therapie oder mit ersten Symptomen einer Krebserkrankung vor. Die Versorgung dieser Patienten stellt häufig eine besondere psychische Belastung für alle Beteiligten dar. Eine entsprechende empathische Kommunikation im notfallmedizinischen Setting sollte sowohl im Team als auch mit dem Patienten und den Angehörigen durchgeführt werden. Auch palliativmedizinische Aspekte einer Versorgung in der Notfallaufnahme (siehe Tab. 16 zu Abschn. 11.10.) sollten bekannt sein und umgesetzt werden.

### 11.7. Infektiologische Aspekte der Akut- und Notfallmedizin (Tab. 13)

**Table 13:** 

WZ 1	Erwerb der für die Betreuung von Patienten erforderlichen Kenntnisse der Klinik, Diagnostik, Prophylaxe und Therapie akut- und notfallmedizinisch relevanter infektiologischer Krankheitsbilder
TK 1	Kenntnisse von Algorithmen zur symptomorientierten Diagnostik bei Akut- und Notfallpatienten sowie einer risikoadaptierten, rationalen mikrobiologischen Diagnostik bei Patienten mit Verdacht auf infektiologische Erkrankungen
TK 2	Kenntnisse akut- und notfallmedizinisch relevanter infektiologischer Krankheitsbilder und deren Therapie
TK 3	Kenntnisse der Prophylaxe akut- und notfallmedizinisch relevanter infektiologischer Krankheitsbilder
TK 4	Kenntnisse akut- und notfallmedizinisch relevanter Hygienerichtlinien und „Antibiotic-Stewardship“-Prinzipien
PF 1	*Sepsis* *Level III: Kenntnis und Implementation von Maßnahmen zur frühen Identifikation von Patienten mit Sepsisverdacht *Level III: Anwendung von Sepsisalgorithmen inklusive spezifischer Diagnostik, patientenangepasster Therapie und Fokussuche/-sanierung
PF 2	*Atemwegsinfektionen* *Level III: Diagnostik und Therapie von Atemwegsinfektionen inklusive Pneumonie
PF 3	*Harnwegsinfektionen* *Level III: Diagnostik und Therapie von Harnwegsinfektionen
PF 4	*Haut- und Weichteilinfektionen* *Level III: Diagnostik und Therapie von Haut- und Weichteilinfektionen
PF 5	*Level III: Indikationsstellung und Umsetzung von Postexpositionsprophylaxen (PEP) nach akutmedizinisch relevantem möglichem Kontakt mit Infektionserregern (inklusive Tetanus, Bisswunden, Meningitis, Hepatitis, HIV)
PF 6	*Level II: Kenntnis, Diagnostik und initiale Therapie der wichtigsten Erkrankungen bei Reiserückkehrern
PF 7	*Level II: Kenntnis und auf lokale Gegebenheiten angepasste Anwendung der Empfehlungen zur Antibiotikatherapie akut- und notfallmedizinisch relevanter infektiologischer Erkrankungen
BV 1	Kooperation mit Infektiologen, Chirurgen, Onkologen, Mikrobiologen und Apothekern beim Management komplexer infektiologischer Probleme und Erstellung diagnostischer und therapeutischer Algorithmen

Anmerkung: Level-I- bis -III-Angaben sind zur Hervorhebung mit einem * versehen

#### Grundlagen und Standards.

Infektionserkrankungen gehören nach wie vor zu den häufigsten Ursachen für eine Vorstellung von Patienten in der Notaufnahme. Das Spektrum reicht von Bagatellinfektionen bis hin zu schweren, akut lebensbedrohlichen Infektionen. Gerade bei schweren Infektionen, wie z. B. der Sepsis, ist die zeitnahe Diagnose und der Beginn einer adäquaten Therapie essenziell. Die Komplexität der Erkrankungen steigt dabei nicht zuletzt aufgrund des Alters und zunehmender Komorbiditäten der versorgten Patienten weiter an. Zusätzlich ist die Notaufnahme für viele Patienten mit der Frage einer Prophylaxe nach möglicher Exposition gegenüber einem infektiösen Erreger die erste Anlaufstelle.

#### Das soll gewusst und gekonnt werden.

- Es ist wichtig, dass der Akut- und Notfallmediziner Erfahrung in der klinischen Präsentation, schnellen, aber rationalen Diagnostik und adäquaten Therapie der häufigsten Infektionserkrankungen besitzt.
- Neben dem Erkennen des klinischen Bilds spielt nicht nur das Behandeln einer akuten Infektion eine wichtige Rolle, sondern auch das Bahnen eines weiteren stationären Aufenthalts durch die initiale Diagnostik und Therapie.
- Neben dem Behandeln von Infektionen muss der Akut- und Notfallmediziner auch Kenntnisse in der Indikationsstellung und Durchführung der wichtigsten Postexpositionsprophylaxen besitzen.
- Es wird außerdem vorausgesetzt, dass der Akut- und Notfallmediziner Kenntnisse über die wichtigsten antiinfektiven Therapien hat. Er soll in der Lage sein, letztere an lokale Gegebenheiten sowie im Rahmen von „Antibiotic Stewardship“ in Zusammenarbeit mit Kollegen anderer Fachrichtungen anzupassen.

### 11.8. Kardiologische Aspekte der Akut- und Notfallmedizin (Tab. 14)

**Table 14:** 

WZ 1	Betreuung akut- und notfallmedizinischer Patienten mit Herz-Kreislauf-Erkrankungen/-Komplikationen inkl. des hypertensiven Notfalls unter Berücksichtigung der erforderlichen Diagnostik und Behandlung der jeweiligen kardiovaskulären Grunderkrankung
*14/1*	*Der kardiale Notfallpatient – generelle Aspekte*
TK 1	Grundkenntnisse derjenigen Herz-Kreislauf-Erkrankungen, bei denen im Falle der Progression/Dekompensation die Gefahr besteht, dass die betroffenen Patienten notfallpflichtig werden (*siehe 14/2-14/9*)
TK 2	Kenntnisse der in der Akut- und Notfallmedizin bei Patienten mit Herz-Kreislauf-Erkrankungen/-Komplikationen eingesetzten Pharmaka
TK 3	Fundierte Kenntnisse der kardialen Bildgebung zur Erkennung gravierender kardiovaskulärer Krankheitsbefunde in Notfallsituationen
TK 4	Grundkenntnisse der temporären mechanischen Herz-Kreislauf-Unterstützung
PF 1	*Level III: leitlinienorientierte Pharmakotherapie, insbesondere deren korrekte Anwendung in Notfallsituationen bei akut- und notfallmedizinischen Patienten mit Herz-Kreislauf-Erkrankungen/-Komplikationen
PF 2	*Level III: situationsgerechtes hämodynamisches Monitoring [78]
PF 3	*Level III: Beherrschung vital bedrohlicher bradykarder und tachykarder Rhythmusstörungen mittels medikamentöser Therapie, Schrittmacherstimulation und Kardioversion/Defibrillation
PF 4	*Level II: Indikationsstellung zur Perikardpunktion bei Perikardergüssen und Perikardtamponaden
PF 5	*Level II: Indikationsstellung zur kardialen Differenzialbildgebung (transthorakale und transösophageale Echokardiographie, Thorax-CT, Koronar-CT, kardiale MRT) zur Abklärung vital bedrohlicher kardiovaskulärer Krankheitsbefunde
PF 6	*Level I: Prüfung der Indikation zur Implantation eines temporären mechanischen Unterstützungssystems bei entsprechender Konstellation
BV 1	Intensive Kommunikation mit Patienten und Familienangehörigen der Patienten hinsichtlich der therapeutischen Vorgehensweise
BV 2	Enge Kooperation des betreuenden Notfallmediziners mit dem Kardiologen/Herzchirurgen hinsichtlich der Betreuung des notfälligen Herz-Kreislauf-Patienten
BV 3	Enge Kooperation mit dem Kardiologen hinsichtlich der Intensivpflichtigkeit eines kardiovaskulären Notfallpatienten sowie des richtigen Zeitpunkts der Verlegung des Patienten mit ausreichender Sicherheit auf die Intensivstation
*14/2*	*Der Patient mit akutem Herzinfarkt (STEMI/NSTEMI; spezifische Aspekte)*
TK 5	Für die Notfallmedizin- und Intensivmedizinphase des Herzinfarktpatienten erforderliches Leitlinienwissen zu Diagnose, Monitoring, und Therapie
PF 7	*Level III: Sicherstellung der Logistikkette bei STEMI-Patienten
PF 8	*Level II: Monitoring der Herzfunktion mittels Echokardiographie einschließlich der Erkennung mechanischer Infarktkomplikationen (Ventrikelseptumdefekt, Ventrikelruptur, akute Mitralinsuffizienz)
*14/3*	*Der Patient mit (infarktbedingtem) kardiogenem Schock (spezifische Aspekte)*
TK 6	Für die Notfallmedizin- und Intensivphase des Patienten mit (infarktbedingtem) kardiogenem Schock erforderliches evidenzbasiertes Wissen zu Diagnose, Monitoring, und Therapie im interprofessionellen Team [30, 79, 81]
PF 9	*Level III: speziell bei Patienten mit infarktbedingtem kardiogenem Schock: Sicherstellung der Logistikkette: möglichst: Notarzt → Herzkatheterlabor (HKL) zur frühestmöglichen Herzkatheteruntersuchung (HKU) und ggf. PCI; andernfalls: Notarzt → Notaufnahme/Chest Pain Unit/kardiologisch-internistischer Schockraum → Intensivstation oder HKL zur frühestmöglichen HKU und ggf. PCI
PF 10	*Level II: Abklärung – gemeinsam mit Kardiologen – der dem kardiogenen Schock zugrunde liegenden Herzerkrankung mittels Anamnese, EKG, HKU und kardialer Bildgebung (insbesondere Echokardiographie, ggf. auch Kardio-CT/„triple-rule out CT“ [82]: akutes Koronarsyndrom bzw. Aortendissektion bzw. Lungenembolie; Pumpfunktionseinschränkung von linkem und/oder rechtem Ventrikel infolge von Herzinfarkt, Kardiomyopathie, Myokarditis oder Perikardtamponade, hochgradigem Vitium, mechanischen Infarktkomplikationen
PF 11	*Level I: Diskussion des leitlinienorientierten [30, 79, 83] Einsatzes eines temporären mechanischen Unterstützungssystems (TMU) bei medikamentös therapierefraktärem (infarktbedingtem) kardiogenen Schock
*14/4*	*Der kritisch kranke Notfallpatient mit dekompensierter Herzinsuffizienz/kardialem Lungenödem (spezifische Aspekte)*
PF 12	*Level II: Abklärung und möglichst Beseitigung der Ursachen der Dekompensation
PF 13	*Level II: Rekompensationstherapie und Beginn/Fortsetzung einer leitlinienorientierten Herzinsuffizienztherapie
PF 14	*Level III: bei Beatmungspflichtigkeit: leitlinienorientierte nichtinvasive bzw. invasive Beatmung
PF 15	*Level II: bei terminaler Herzinsuffizienz: Möglichkeit der Implantation eines permanenten Herzunterstützungssystems bzw. einer Herztransplantation gemeinsam mit Kardiologen und Herzchirurgen in Erwägung ziehen
*14/5*	*Der Patient mit dekompensiertem angeborenem oder erworbenem Vitium, Kardiomyopathie, Herzbeteiligung bei systemischer Erkrankung einschließlich kardialer Auswirkungen einer Tumorerkrankung oder einer Tumortherapie sowie mit thorakaler Aortendissektion (spezifische Aspekte)*
PF 16	*Level II bzw. *Level I je nach anzuwendender Methode: Erfahrungen in der nichtinvasiven und invasiven bildgebenden Diagnostik sowie einer ggf. notwendigen rhythmologischen Diagnostik
PF 17	Indikationsstellung und Durchführung konservativ-medikamentöser Therapiemaßnahmen (*Level II) sowie Indikationsstellung zur primär- oder sekundär-prophylaktischen Implantation aktiver Herzrhythmusimplantate (*Level I), der interventionellen Therapie (einschließlich eines TMU; *Level I) oder der operativen Therapie (einschließlich der Herztransplantation; *Level I) in Absprache mit einem Kardiologen/ggf. Herzchirurgen
PF 18	*Level I: Abklärung der Indikationsstellung zur Myokardbiopsie
BV 4	Fallbezogen enge Zusammenarbeit mit jeweils beteiligten Fachgebieten (Kardiologie, Herzchirurgie, Rheumatologie, Pneumologie, Hämatologie/Onkologie, Humangenetik) zur zeitnahen Diagnosestellung und Behandlung
BV 5	Patientenadaptierte Vorgehensweise der Kollegen aller beteiligten Fachgebiete bei der Information von Patienten und deren Angehörigen im Fall einer angeborenen Kardiomyopathie bezüglich der weiteren Vorgehensweise
*14/6*	*Der kritisch kranke Patient mit infektiöser Endokarditis* (spezifische Aspekte)
TK 7	Rasches Erkennen, Diagnostizieren und Behandeln des infektiösen Endokarditisfokus (Nativklappe, prothetische Klappe inkl. interventionell implantierte Klappe und herz- und gefäßbezogenes prothetisches Material) bzw. des infizierten Schrittmachers, Defibrillators, herznahen Katheters oder eines TMU (Device)
PF 19	*Level III: leitlinienorientierte Probengewinnung zur mikrobiologischen Diagnostik
PF 20	*Level II: Durchführung der leitlinienorientierten antiinfektiven Therapie nach eindeutiger Endokarditisdiagnose in möglicher Absprache mit Infektiologen/Mikrobiologen und insbesondere Kardiologen
PF 21	*Level I: leitlinienorientierte Durchführung der bildgebenden Diagnostik mittels transthorakaler und transösophagealer Echokardiographie
BV 6	Indikationsstellung zur operativen Behandlung gemeinsam mit Kardiologen und Herzchirurgen
BV 7	Kooperation bei der antimikrobiellen Diagnose und Therapie mit Mikrobiologen/Infektiologen und Kardiologen
*14/7*	*Der kritisch kranke Patient mit Myokarditis (spezifische Aspekte)*
PF 22	Falls die Krankheitsakuität die Entscheidungen noch in der Notaufnahmestation erforderlich macht: Indikationsstellung und Durchführung konservativ-medikamentöser Therapiemaßnahmen (*Level II) sowie Indikationsstellung zur primär- oder sekundär-prophylaktischen Implantation aktiver Herzrhythmusimplantate (*Level I), der interventionellen Therapie (einschließlich eines TMU; *Level I) oder der operativen Therapie (einschließlich der Herztransplantation; *Level I) in Absprache mit einem Kardiologen/ggf. Herzchirurgen
PF 23	*Level I: Falls die Krankheitsakuität die Entscheidungen noch in der Notaufnahme erforderlich macht: Indikationsstellung zur Myokardbiopsie
BV 8	Enge Zusammenarbeit mit Kardiologen, Pathologen und Herzchirurgen
*14/8*	*Der kritisch kranke Patient mit Perikarderkrankungen (spezifische Aspekte)*
*TK 8*	Für die notfallmäßige Betreuung relevante Aspekte von Perikarderkrankungen, insbesondere von großen bzw. tamponierenden Perikardergüsse unterschiedlicher Ätiologie (Perikarditis, Tumortherapie) und Pericarditis constrictiva
PF 24	*Level III: Indikationsstellung und Durchführung einer Perikardpunktion bei Perikardtamponade
PF 25	*Level I: Interpretation der Ergebnisse der nichtinvasiven bildgebenden und ggf. notwendigen invasiven Diagnostik
PF 26	*Level II: konservative Therapie der jeweiligen Perikarderkrankung
BV 9	Bei Vorliegen rezidivierender Perikardergüsse oder einer konstriktiven Perikarditis: gemeinsame Beratung mit Kardiologen und Herzchirurgen bezüglich einer ggf. operativen Therapie
*14/9*	*Der Herzpatient mit vital bedrohlichen bradykarden oder tachykarden Rhythmusstörungen (spezifische Aspekte)*
*TK 9*	Kenntnisse über die bei der jeweils spezifischen Herz-Kreislauf-Erkrankung erwartbaren vital bedrohlichen bradykarden und tachykarden Herzrhythmusstörungen
PF 27	*Level III: kontinuierliches EKG-Monitoring inkl. verlässlicher Dokumentation auftretender Herzrhythmusstörungen
PF 28	*Level III: medikamentöse antiarrhythmische Therapie zur Unterdrückung der jeweiligen vital bedrohlichen brady- oder tachykarden Rhythmusstörung
PF 29	*Level III: kardiale Elektrotherapie (siehe PF 3)
PF 30	*Level I: selten erforderliche spezifische antiarrhythmische medikamentöse oder interventionelle Therapieoptionen
*14/10*	*Hyertensiver Notfall/hypertensive Dringlichkeit*
PF 31	*Level III: Abschätzung des Gefährdungsgrads des hypertensiven Patienten durch Klassifizierung des Bluthochdrucks entweder als hypertensiver Notfall oder als hypertensive Dringlichkeit ([77]; siehe auch Begleittext)
PF 32	Hypertensiver Notfall:
	*Level III: beim hypertensiven Notfall Einleitung der antihypertensiven Therapie und Behandlung der Endorganschädigung [77]
*14/11*	*Der kritisch kranke Patient mit Herzschrittmacher/Defibrillator (spezifische Aspekte)*
TK 10	Rasches Erkennen, Diagnostizieren und Behandeln einer Herzschrittmacher‑/Defibrillatorfehlfunktion („oversensing“/„undersensing“)
PF 33	*Level I: Grundzüge des Erkennens und des Abstellens von Herzschrittmacher‑/Defibrillator(fehl)funktionen
PF 34	*Level III: kontinuierliches EKG-Monitoring inkl. der Dokumentation der Herzschrittmacher‑/Defibrillatorfehlfunktion
*14/12*	*Der kritisch kranke Patient nach Herztransplantation (spezifische Aspekte)*
TK 11	Rasches Erkennen, Diagnostizieren und Behandeln einer akuten Abstoßungsreaktion
PF 35	*Level I: Grundzüge der Diagnostik und Behandlung einer akuten Abstoßungsreaktion inkl. Indikationsstellung zur Myokardbiopsie
PF 36	*Level I: Durchführung einer leitlinienorientierten Abstoßungsbehandlung in Absprache mit dem Kardiologen und Pathologen

Anmerkung: Level-I- bis -III-Angaben sind zur Hervorhebung mit einem * versehen

#### Grundlagen und Standards.

- Die kardiovaskuläre Notfall‑, Akut- und Intensivmedizin [15] macht aufgrund der Häufigkeit kardiovaskulärer Erkrankungen einen beträchtlichen Teilbereich dieser Disziplinen aus. Bei einem relevanten Anteil der Patienten ist eine kompetente Versorgung zeitkritisch, um die bestmögliche Diagnostik und Therapie zu gewährleisten. Bei den kardialen Erkrankungen ist von besonderer Bedeutung, dass nicht nur einige eigenständige Krankheitsbilder von zentraler Relevanz sind, sondern auch kardiovaskuläre Erkrankungen als Begleitumstände anderer Krankheitsbilder hochprävalent sind.
- Erforderlich sind grundlegende Kenntnisse der kardiovaskulären Anatomie, Physiologie und Pathophysiologie, Kenntnisse der für die klinische Akut- und Notfallmedizin relevanten akuten und chronischen kardialen Erkrankungen als eigenständige Krankheitsbilder und auch deren Relevanz als Komorbiditäten, der Verfahren zur Diagnosestellung (inkl. bildgebender Verfahren) und Kenntnisse der Therapie akuter Funktionsstörungen. Das Erkennen eines kardialen Problems (myokardial, strukturell, rhythmologisch) muss gewährleistet sein mit dem Ziel, häufige zeitkritische Therapiemaßnahmen und nachgeschaltete kardiologische Spezialdiagnostik und/oder -therapie einzuleiten. Insbesondere sollen notwendige medikamentöse Therapiestrategien selbstständig angewendet und Sofortmaßnamen, wie kardiopulmonale Reanimation, Kardioversion und Defibrillation, eigenständig durchgeführt werden können. Kenntnisse der (Notfall‑/Basis‑)Echokardiographie sollen vorhanden sein, ebenso wie grundlegende Kenntnisse der Sonographie und der Befundung konventioneller Röntgenaufnahmen des Thorax sowie der thorakalen Computertomographie und kardialen Magnetresonanztomographie.
- Hypertensive Dringlichkeit und hypertensiver Notfall [76, 77]: Bei einer Prävalenz der arteriellen Hypertonie von 30–50 % in Deutschland ist es nicht verwunderlich, dass jeder 10. Hochdruckpatient im Lauf seines Lebens eine hypertensive Notfallsituation erleidet. Bei etwa 5 von 1000 Fällen ist eine hypertensive Notfallsituation der Aufnahmegrund in der Notaufnahme. Blutdruckwerte > 180/110 mm Hg mit Organschädigung (akutes Lungenödem: 30 %; Schlaganfall: 20 %; Myokardinfarkt: 20 %; akute Nierenschädigung: 5 %) werden als hypertensiver Notfall klassifiziert und bedürfen einer sofortigen Behandlung in der Notaufnahme. Die Letalität eines nicht adäquat behandelten hypertensiven Notfalls wird auf etwa 10 % innerhalb der nächsten 5 Jahre geschätzt. Blutdruckwerte > 180/110 mm Hg ohne Endorganschädigung werden als hypertensive Dringlichkeit bezeichnet; hierbei wird eine zeitnahe ambulante Abklärung empfohlen.

#### Das soll gewusst und gekonnt werden.

- **Kardiologische(s) Notfalldiagnostik, -behandlung und -monitoring: **Für die zeitgerechte Diagnostik, Überwachung und Therapie von Akut- und Notfallpatienten ist eine Reihe von Fähigkeiten von besonderer Relevanz. Dazu gehören die Notfallechokardiographie/-sonographie, das hämodynamische (Basis‑)Monitoring [78] und das Management der akuten Herzinsuffizienz (inklusive des akuten Rechtsherzversagens und infolge eines dekompensierten Vitiums), das Erkennen und Versorgen des akuten Koronarsyndroms (ACS) inkl. des Patienten mit Myokardinfarkt (NSTEMI und ST-Hebungs-Infarkt [STEMI]), das Management des (infarktbedingten) kardiogenen Schocks [79], das Erkennen und die Behandlungsinitiierung eines Perikardergusses bzw. einer Perikardtamponade, Kenntnisse zu mechanischen Kreislaufunterstützungsverfahren sowie Notfallrhythmusdiagnostik und akutes Arhythmiemanagement. Darüber hinaus sind die Fähigkeiten zur Reanimation/Advanced Life Support (ALS) essenziell, ebenso wie diejenigen zu den Leitsymptomen der Themenkomplexe „Dyspnoe und Brustschmerz“, „Hypertensive Notfälle“, „Hypotonie und Synkope“ und „Herzerkrankung bei speziellen Patientengruppen“ (HIV, Schwangerschaft, Tumorpatienten, Patienten mit pulmonalarteriellem Hochdruck, Transplantationspatienten, akutes Rechtsherzversagen). Die Pharmakotherapie in der kardiovaskulären Notfallmedizin soll beherrscht werden, ebenso wie das Basismanagement bei notfälligen Devicepatienten mit Herzschrittmacher oder Defibrillator [80].
- **Spezifische diagnostische und therapeutische Fähigkeiten: **Der Akut- und Notfallmediziner soll über Kenntnisse verfügen, die es ihm ermöglichen, eigenständig oder in Kooperation folgende diagnostische und therapeutische Maßnahmen in Bezug auf oben genannte Krankheitsbilder anzuwenden.
  - Die diagnostischen Fähigkeiten beinhalten:
    - die EKG-Beurteilung [40];
    - die transthorakale Echokardiographie sowie Grundzüge der transösophagealen Echokardiographie [39];
    - den fokussierten Gefäßultraschall [33];
    - Indikation zur Linksherzkatheteruntersuchung;
    - Indikation zur Rechtsherzkatheteruntersuchung;
    - Basiskenntnisse in der Herzschrittmacher- und Deviceabfrage;
    - Indikation zur Perikardpunktion und Myokardbiopsie;
    - kardiale Biomarker;
    - Basiskenntnisse in der Indikationsstellung und Diagnostik mittels Kardio-CT und Kardio-MRT.
  - Die therapeutischen Fähigkeiten beinhalten:
    - die Absprache mit einem Facharzt für Innere Medizin und Kardiologie zur Indikationsstellung zur akuten perkutanen Koronarintervention (PCI);
    - die leitliniengerechte Initiierung einer Inotropika‑/Vasopressorentherapie;
    - die passagere perkutane Schrittmachertherapie, Kardioversion, Defibrillation und Überstimulation;
    - die Postreanimationsphase einschließlich des zielgerichteten Temperaturmanagements;
    - die Indikationsstellung mechanischer Herz-Kreislauf-Unterstützungssysteme nach Anlegen in einem Herzkatheterlabor oder im Schockraum;
    - die systemische Thrombolyse sowie Grundprinzipien der Thrombektomie und Thrombusfragmentierung inklusive der Indikationsstellung in Absprache mit dem jeweiligen Fachkollegen;
    - ein adäquates medikamentöses Therapiemanagement des hypertensiven Notfalls.

### 11.9. Nephrologische Aspekte der Akut- und Notfallmedizin (Tab. 15)

**Table 15:** 

WZ 1	Notfallversorgung von Patienten mit den verschiedenen Stadien der akuten und chronischen Nierenfunktionseinschränkung
*15/1*	*Akute Nierenfunktionseinschränkung (AKI)*
WZ 2	Fähigkeit zur Diagnose, Behandlung und Prävention einer AKI. Korrekte klinische Allokation eines Patienten mit AKI
TK 1	Diagnose und Differenzialdiagnose der AKI; korrekte Einschätzung des vorliegenden Flüssigkeitsstatus
TK 2	Anamnese unter besonderer Berücksichtigung der die Niere involvierenden Erkrankungen inkl. Systemerkrankungen (Sepsis, Vaskulitiden, pulmorenale Syndrome)
TK 3	Differenzialdiagnose der AKI einschließlich kardiorenaler, pulmorenaler und hepatorenaler Syndrome, der rapid progressiven Glomerulonephritis (RPGN) und von Nephrotoxizitäten
PF 1	*Level II: Diagnostik einer AKI; Säure-Basen-Analytik; Urämiesymptome erfassen; Hyperkaliämie im EKG erfassen; Sonographie der Nieren
PF 2	*Level III: Festlegung einer medikamentösen Akuttherapie (Volumen, Diuretika, Flüssigkeit, Beseitigung einer Volumenüberladung)
PF 3	*Level II: Erkennen der Indikation für eine akute Nierenersatztherapie
BV 1	Fähigkeit zur engen Zusammenarbeit mit dem Nephrologen in der Diagnostik und Therapie akuter Nierenerkrankungen
*15/2*	*Chronische Nierenerkrankung („chronic kidney disease“, CKD)*
WZ 3	Fähigkeit zur Diagnose einer CKD; Kenntnisse der akuten Komplikationen eines CKD-Patienten; korrekte klinische Allokation eines Patienten mit CKD je nach Schweregrad und Komplikationslevel
TK 4	Diagnose und Einteilung einer CKD
TK 5	Anamnese unter besonderer Berücksichtigung der mit einer CKD assoziierten kardiovaskulären und metabolischen Begleiterkrankungen
TK 6	Kenntnisse der chronischen Nierenersatzverfahren und seiner für die Notfallmedizin relevanten Besonderheiten (insbesondere: Peritonealdialyse: Peritonitis; Hämodialyse: Shuntverschluss, Unterdialyse und Überwässerung; Nierentransplantation: Abstoßung, postrenale Abflussprobleme, Infektionen bei Immunsuppression)
PF 4	*Level III: Diagnostik einer CKD (Säure-Basen-Analytik; Hyperkaliämie: EKG; Überwässerung; Anämie; kardiovaskuläre Akutkomplikationen)
PF5	*Level II: Festlegung einer medikamentösen Akuttherapie (Volumen, Diuretika, Flüssigkeit, Beseitigung einer Volumenüberladung) oder Weiterleitung an Dialyseabteilung
PF 6	*Level II: Shuntmanagement (akute Blutung, Shuntverschluss)
BV 2	Fähigkeit zur engen umfassenden Koordination mit dem Nephrologen, Radiologen und Gefäßchirurgen in der adäquaten Weiterbehandlung von Patienten mit chronischen Nierenerkrankungen
*15/3*	*Thrombotische Mikroangiopathie (TMA)*
WZ 4	Fähigkeit zur Diagnose einer thrombotischen Mikroangiopathie (TMA), insbesondere einer thrombotisch-thrombozytopenischen Purpura (TTP) und eines hämolytisch-urämischen Syndroms (HUS)
TK 7	Kenntnisse der TMA und seiner wichtigen Formen, der TTP und des HUS
PF 7	*Level I: Fähigkeit zur selbständigen zielführenden Diagnostik einer TMA
*15/4*	*Elektrolytstörungen (Natrium)*
WZ 5	Grundlagen der Volumen- und Osmoregulation
TK 8	Hypo- und Hypernatriämie; SIADH
PF 8	*Level III: Notfalltherapie
	*Level II: Fähigkeit zur zielführenden Diagnostik einer Hypo- bzw. Hypernatriämie inkl. Serumosmolaritat, Urinnatrium, Urinosmolaritat sowie der Indikation und Durchführung einer medikamentösen Akuttherapie
*15/5*	*Elektrolytstörungen (Kalium)*
TK 9	Hypo- und Hyperkaliämie
PF 9	*Level III: Fähigkeit zur zielführenden Diagnostik einer Hypo- bzw. Hyperkaliämie und zur Durchführung einer medikamentösen bzw. extrakorporalen Akuttherapie
	*Level III: Notfalltherapie
PF 10	*Level II: Fähigkeit zur raschen Indikationsstellung einer Akutdialyse bei therapierefraktärer Hyperkaliämie und notfallmäßige Anlage eines passageren Doppellumenkatheters
*15/6*	*Elektrolytstörungen (Kalzium, Phosphat, Magnesium)*
TK 10	Störungen des Kalzium‑, Phosphat- und Magnesiumhaushalts
PF 11	*Level II: Fähigkeit zur zielführenden Diagnostik einer Hypo- bzw. Hyperkalzämie sowie zur Durchführung einer medikamentösen Akuttherapie sowie adäquates Management der Störungen des Kalzium‑, Phosphat- und Magnesiumhaushalts
	**Level II:** Indikationsstellung einer extrakorporalen Akuttherapie
BV 3	Fähigkeit zur Koordination und Zusammenarbeit mit Nephrologen und/oder Onkologen
*15/7*	*Störungen des Säure-Basen-Haushalts*
WZ 6	Bedeutung der Aufrechterhaltung eines konstanten pH-Werts für Zellphysiologie und den Elektrolythaushalt; Puffersysteme, Atemphysiologie und Kompensationsmechanismen zur pH-Kontrolle
TK 11	Metabolische Azidose/Alkalose und respiratorische Azidose/Alkalose
TK 12	Bestimmung und Bedeutung der Anionenlücke
PF 12	*Level III: selbständige Indikationsstellung zur zielführenden Diagnostik einer Störung des Säure-Basen-Haushalts inkl. der Durchführung einer arteriellen Blutgasanalyse und deren Interpretation mit Bestimmung der Anionenlücke
PF 13	*Level III: Fähigkeit zur selbständigen Durchführung der Akuttherapie bestehender Störungen des Säure-Basen-Haushalts sowohl im metabolischen als auch im respiratorischen Bereich

Anmerkung: Level-I- bis -III-Angaben sind zur Hervorhebung mit einem * versehen

#### Grundlagen und Standards.

Grundlagenkenntnisse der Nierenphysiologie, Nierenfunktion und Urindiagnostik sind erforderlich, ebenso das Wissen um die für die Akutmedizin relevanten akuten und chronischen Nierenerkrankungen.

#### Das soll gewusst und gekonnt werden.

- Die **akute Nierenfunktionseinschränkung** („acute kidney injury“, AKI) ist das häufigste sepsisassoziierte Organversagen in der Notfall- und Intensivmedizin. Dafür werden Kenntnisse und Handlungskompetenz benötigt:
  - zur spezifischen Pathophysiologie des AKI sowie des Organ-Crosstalks;
  - zur Diagnostik, Differenzialdiagnostik und Therapie kardiorenaler, hepatorenaler sowie pulmorenaler Syndrome als Manifestation von Systemerkrankungen;
  - vertieft zur Indikation und Durchführung einer medikamentösen Akuttherapie des AKI sowie der Indikationen zur Einleitung einer Nierenersatztherapie;
  - zur Beherrschung der standardisierten Nierensonographie, unter anderem zum Ausschluss prä- und postrenaler Ursachen des AKI;
  - zum Verständnis für Langzeitfolgen eines AKI einschließlich der Notwendigkeit einer nephrologischen Nachsorge.
- **Chronische Nierenerkrankung**:
  - Einteilung der chronischen Nierenerkrankung („chronic kidney disease“, CKD) in Schweregrade und der sich daraus ergebenden medizinischen Konsequenzen;
  - Kenntnisse der sich daraus ergebenden Labor- sowie Urindiagnostik;
  - Verständnis, dass Patienten mit chronischer Dialysetherapie rasch an Zentren mit Dialysemöglichkeit und Nephrologie weiterzuvermitteln sind.
- **Thrombotische Mikroangiopathie (TMA)**:
  - Diagnostik und Therapie der verschiedenen Formen einer mit einem AKI einhergehenden TMA, insbesondere einer thrombotisch-thrombozytopenischen Purpura (TTP) und eines hämolytisch-urämischen Syndroms (HUS) anhand der klinischen Trias „Thrombopenie, Hämolyse und Fragmentozyten“;
  - Verständnis, dass Patienten mit einem TMA-Verdacht sofort in nephrologische oder hämatologische Behandlung weiter verwiesen werden müssen.
- **Elektrolytstörungen**:
  - Physiologie und Pathophysiologie der Volumen- und Osmoregulation sowie der Homeostase des Kalium‑, Kalzium‑, Phosphat- und Magnesiumhaushalts;
  - Elektrolytstörungen und die damit verbundenen akut bedrohlichen Krankheitsbilder der Notfallmedizin;
  - Akuttherapie vital bedrohlicher Elektrolytstörungen einschließlich der Indikationen zur Durchführung einer Akutdialyse [84].
- **Störungen des Säure-Basen-Haushalts**:
  - Bedeutung der Aufrechterhaltung eines konstanten pH-Werts für Zellphysiologie und Elektrolythaushalt sowie der Kompensationsmechanismen zur Konstanthaltung des pH-Werts;
  - zielführende Diagnostik einer Störung des Säure-Basen-Haushalts inkl. der Durchführung einer arteriellen oder venösen Blutgasanalyse und deren Interpretation, auch mit Bestimmung der Anionenlücke.

### 11.10. Palliativmedizinische Aspekte der Akut- und Notfallmedizin (Tab. 16)

**Table 16:** 

WZ 1	Verständnis für die Komplexität der Situation bei Patienten mit unheilbaren fortgeschrittenen Erkrankungen und in der letzten Lebensphase
PF 1	*Level II: „Total-pain“-Konzept anwenden können (Berücksichtigung einer physischen, psychischen, sozialen und spirituellen Dimension von Leid)
WZ 2	Einbeziehung und Unterstützung der An- und Zugehörigen
PF 2	*Level III: Gesprächskompetenz
WZ 3	Strukturen der allgemeinen und spezialisierten Palliativversorgung
TK 1	Palliativstation, Palliativdienst, allgemeine ambulante Palliativversorgung (AAPV), spezialisierte ambulante Palliativversorgung (SAPV), mögliche Erreichbarkeit/Kontakte in Notfallsituationen benennen
WZ 4	Krankheit, Sterben, Tod und Trauer in verschiedenen Kulturen und Religionen
BV 1	Kultursensibler Umgang mit Schwerkranken und ihren Nahestehenden
WZ 5	Ethische und rechtliche Grundprinzipien der Patientenversorgung
TK 2	Rechtliche Rahmenbedingungen zu Indikationsstellung, Stellenwert des Patientenwillens und Therapiezielfindung erläutern können
PF 3	*Level III: ethische Grundprinzipien auf Notfallsituationen sicher anwenden können, um zu einer ethischen Entscheidungsfindung zu gelangen
WZ 6	Umgang mit existenzieller Angst
BV 2	Auseinandersetzung mit der praktischen Relevanz existenzieller Angst und Diversität am Lebensende in Notfallsituationen
WZ 7	Interprofessionelle und interdisziplinäre Teamarbeit
TK 3	Die Handlungsfelder der einzelnen Berufsgruppen/Disziplinen benennen können
PF 4	*Level III: im interprofessionellen und interdisziplinären Team auf Augenhöhe zusammenarbeiten
BV 3	Die eigenen Grenzen und die der anderen Teammitglieder wahrnehmen und respektieren
WZ 8	Management von körperlichen und psychischen Krisen
PF 5	*Level II: Symptomlinderung unter Berücksichtigung des „Total pain“-Konzepts (Berücksichtigung einer physischen, psychischen, sozialen und spirituellen Dimension von Leid)
WZ 9	Behandlung von Symptomen, Schmerzen und Komplikationen bei unheilbaren fortgeschrittenen Erkrankungen und in der letzten Lebensphase
PF 6	*Level III: Behandlung gastrointestinaler Symptome bei unheilbaren fortgeschrittenen Erkrankungen und in der letzten Lebensphase
PF 7	*Level III: Behandlung pulmonaler Symptome bei unheilbaren fortgeschrittenen Erkrankungen und in der letzten Lebensphase
PF 8	*Level II: Behandlung neurologischer Symptome bei unheilbaren fortgeschrittenen Erkrankungen und in der letzten Lebensphase
PF 9	*Level III: Schmerzbehandlung; bei komplexer Natur: *Level II
WZ 10	Therapierefraktäre Symptomkrise
PF 10	*Level I: Betreuung des Patienten mit therapierefraktärer Symptomkrise
WZ 11	Sterbephase
PF 11	*Level II: Betreuung des Patienten in der Sterbephase
WZ 12	Beurteilung der Angemessenheit von Therapiemaßnahmen unter Berücksichtigung des Therapieziels
TK 4	Grundsätze der Therapiezielfindung erläutern können
PF 12	*Level III: ein ethisches Team „Timeout“ in der Notfallversorgung Schwerkranker sicher anwenden (analog „10-for-10“: Welches realistische Therapieziel gibt es? Was wissen wir über den Patientenwunsch? Mit welchen nächsten Schritten können wir das Therapieziel erreichen?)
BV 4	Reflexion von Notfallsituationen mit gelungener und misslungener Therapiezielfindung im Team
WZ 13	Individuelles Symptomempfinden und Leiderfahrung der Patienten
BV 5	Die Subjektivität von Leid in der Notfallsituation angemessen berücksichtigen

Anmerkung: Level-I- bis -III-Angaben sind zur Hervorhebung mit einem * versehen

#### Grundlagen und Standards [85].

**Figure 1:**
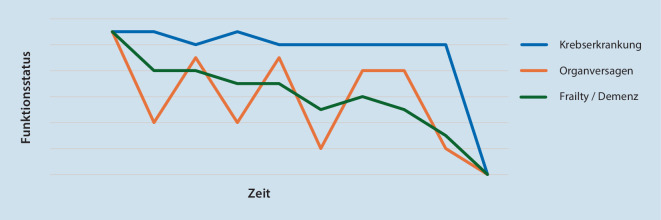

Die demographische Entwicklung und die Weiterentwicklung medizinischer Behandlungsmöglichkeiten führen dazu, dass insbesondere die Innere Medizin mit einer zunehmenden Zahl an multimorbiden Patienten mit komplexen Erkrankungssituationen und palliativmedizinischem Behandlungsbedarf konfrontiert ist. Neben den onkologisch/hämatologisch Erkrankten betrifft dies auch die große Gruppe der Patienten mit progredientem Organversagen (COPD, Herzinsuffizienz, Niereninsuffizienz) sowie hochaltrige, gebrechliche und demente Patienten. In Bezug auf eine angemessene Therapiezielfindung stellen die sehr unterschiedlichen Krankheitsverläufe die Behandler – insbesondere in Akut- und Notfallsituationen – häufig vor große Herausforderungen (Abb. 1).

Notfälle bei Patienten in palliativer Situation lassen sich in folgende Konsultationsanlässe clustern [86]:

- Probleme ohne Zusammenhang zur Grunderkrankung;
- neu aufgetretene Probleme im Zusammenhang mit der Grunderkrankung;
- therapieassoziierte Probleme;
- Exazerbation bekannter Symptome;
- „total pain“;
- Überforderung des sozialen Umfelds.

Es gilt in diesen Situationen – basierend auf der mutmaßlichen Prognose – neben dem üblichen notfallmedizinischen Therapieziel „Hilfe zum Leben“, auch weitere mögliche Ziele zu eruieren, wie „Hilfe im Sterben“ oder „Hilfe zur rechten Zeit zu sterben, bis dahin aber bestmöglich zu leben“ [86].

#### Das soll gewusst und gekonnt werden.

Um den in „Grundlagen und Standards“ genannten Anforderungen an eine palliativmedizinisch orientierte Notfallbehandlung gerecht zu werden, sollen die in Tab. 16 aufgeführten Weiterbildungsziele (WZ; siehe auch [87]) berücksichtigt werden.

### 11.11. Pneumologische Aspekte der Akut- und Notfallmedizin (Tab. 17)

**Table 17:** 

WZ 1	Grundlagen der Lungenanatomie, -physiologie und -pathophysiologie sowie der für die Akut- und Notfallmedizin relevanten akuten und chronischen Lungenerkrankungen; Verfahren zur Diagnosestellung und Materialgewinnung sowie zur Therapie pneumologischer Notfälle
*17.1*	*Erforderliche Fähigkeiten bei der Betreuung von Notfallpatienten mit pneumologischen Erkrankungen*
PF 1	*Level III: Erkennen und Ersteinschätzung akuter pneumologischer Erkrankungen (A[temwegs]- und B[eatmungs]-Probleme); selbstständige Diagnosestellung inklusive der Interpretation von Befunden und der Therapieeinleitung
PF 2	*Level III: selbstständige Einleitung einer nichtinvasiven Beatmung mit Indikationsstellung/Kontraindikationen; Beatmungseinstellungen und Begrenzung der Therapiemöglichkeiten
PF 3	*Level III: selbstständige Atemwegssicherung zur Behebung eines A‑ und/oder B‑Problems; Beherrschen der Hämodynamik nach Narkoseeinleitung bei kritisch kranken Patienten
PF 4	*Level II: Durchführung einer Bronchoskopie zur Diagnostik und Therapie bei A‑ und/oder B‑Problemen
PF 5	*Level III: Anlage einer Thoraxdrainage in Seldinger-Technik zur Behebung eines Pneumothorax/Pleuraergusses jedweder Genese
BV 1	Erkennen der Indikation zur Verlegung in eine angemessen höhere Versorgungsstufe nach initialer Versorgung und Behebung des akuten Problems
BV 2	Sinnvolle Zusammenfassung relevanter Krankheitsfakten zu einer Übergabe an (pneumologische) Kollegen/enge Zusammenarbeit mit (pneumologischen) ärztlichen und atmungstherapeutischen Kollegen
BV 3	„End-of-life“-Gespräche mit Angehörigen bei Vorliegen lebenslimitierender pneumologischer Erkrankungen
BV 4	Enge Zusammenarbeit mit Anästhesisten, Radiologen, Thoraxchirurgen und anderen Ärzten von Fachabteilungen (einschließlich, aber nicht limitiert auf Nephrologie, Rheumatologie, Kardiologie), die in die Versorgung akuter pneumologischer Erkrankungen involviert sind
BV 5	Kenntnisse juristischer Grundlagen im Zusammenhang mit Patientenverfügung, „advance care planning“, und Palliativversorgung
*17.2*	*Akute A(temwegs)- und/oder B(eatmungs)-Probleme*
WZ 2	Erkennen, Ersteinschätzung und Behandlung von A‑ und/oder B‑Problemen
TK 1	Physiologie und Pathophysiologie der Atmung und der Atemmechanik
TK 2	Therapieoptionen bei A‑ und/oder B‑Problemen in Abhängigkeit von der Genese
TK 3	Unterscheidung verschiedener Formen des Pneumothorax
PF 6	*Level III: Anlage einer Thoraxdrainage (in Seldinger-Technik)
*17.3*	*Respiratorisches Versagen – akutes Lungenversagen (hypoxisch/hyperkapnisch)*
WZ 3	Fähigkeit zur (Differenzial‑)Diagnosestellung und Behandlung des respiratorischen Versagens jedweder Genese
TK 4	Anamnese (Eigen‑/Fremdanamnese) inkl. Berufs- und Reiseanamnese; Basiskenntnisse der Allergologie
TK 5	Ventilatorisches Versagen
TK 6	Oxygenierungsversagen
TK 7	Obstruktive und restriktive Lungenerkrankungen
TK 8	Grundkenntnisse der Bedeutung der fortgeschrittenen COPD in der Akut- und Langzeitprognose
TK 9	Indikationen, Kontraindikationen und Limitationen einer antiobstruktiven Therapie
TK10	Grundkenntnisse der Bedeutung häufiger interstitieller Lungenerkrankungen in der Akut- und Langzeitprognose
PF 7	*Level III: Einstellung der Beatmung (nichtinvasiv, invasiv) bei obstruktiven und restriktiven Ventilationsstörungen
*17.4*	*Pulmonale und bronchiale Blutungen*
WZ 4	Fähigkeit zur (Differenzial‑)Diagnosestellung und Behandlung einer Lungenparenchym- oder endobronchialen Blutung; Grundkenntnisse der Bedeutung häufiger rheumatischer Lungenerkrankungen in der Akut- und Langzeitprognose
TK11	Ätiologie und Pathogenese von Hämoptysen und Hämoptoe
TK12	Optionen (in interdisziplinärer Zusammenarbeit mit interventionellen Radiologen und Thoraxchirurgen)
TK13	Akute Tumorblutung inkl. Palliation
PF 8	*Level I: Anwenden eines Bronchusblockers – gemeinsam mit einem Pneumologen – zur akuten Blutstillung
	*Level I: Endoskopie bei Lungenblutung
*17.5*	*Pulmonalvaskuläre Erkrankungen*
WZ 5	Fähigkeit zur (Differenzial‑)Diagnosestellung und Behandlung einer akuten und chronischen pulmonalen Hypertonie (PH) sowie des obstruktiven Schocks
TK14	Lungenarterienembolie: Diagnostik
TK15	Lungenarterienembolie: Indikationsstellung der stadienadaptierten Therapie
TK16	Lungenarterienembolie: Durchführung der stadienadaptierten Therapie
TK17	Lungenarterienembolie: Indikationsstellung für interventionelle Verfahren inkl. va-ECMO
PF 9	Lungenarterienembolie: *Level III: Durchführung einer systemischen Lysetherapie
PF10	Lungenarterienembolie: *Level I: Indikationsstellung und Anlage einer va-ECMO
*17.6*	*Verletzungen des Thorax und damit verbundene Probleme*
WZ 6	Kenntnisse über thorakale Verletzungen und damit verbundener Probleme (einschließlich, aber nicht limitiert: stumpfes und penetrierendes Thoraxtrauma, iatrogene und traumatische Verletzungen der Atemwege)
TK18	Pneumothorax/Spannungspneumothorax
TK19	Rippenserienfraktur („flail chest“)
TK20	Lungenkontusion
TK21	Hämatothorax
PF11	*Level II: Anlage einer Thoraxdrainage in Minithorakotomietechnik
PF12	*Level I: endoskopische Fremdkörperentfernung auch bei Säuglingen und Kleinkindern mit Pneumologen
*17.7*	*Infektiöse Lungenerkrankungen*
WZ 7	Fähigkeit zur (Differenzial‑)Diagnosestellung und Behandlung von infektiösen Lungenerkrankungen
TK22	Bronchitis, Bronchiolitis, Pneumonie, Empyem, Lungenabszess, Pleuritis und Pleuraerguss, Tuberkulose, Influenza, SARS-CoV‑2
TK23	Kenntnisse antiinfektiver Therapie bei Infektionserkrankungen mit pulmonalem Fokus
PF13	*Level II: Abnahme von erregerhaltigem Material aus den zentralen Atemwegen
PF14	*Level I: Abnahme von erregerhaltigem Material aus den tiefen Atemwegen inkl. BAL mithilfe eines Pneumologen
*17.8*	*Angeborene Lungenerkrankungen/sonstige Erkrankungen mit potenziellen A(temwegs)- und B(eatmungs)-Problemen*
WZ 8	Grundkenntnisse der Bedeutung angeborener Lungenerkrankungen sowie neuromuskulärer Erkrankungen in der Akut- und Langzeitprognose
TK24	Mukoviszidose
TK25	Neuromuskuläre Erkrankungen
*17.9*	*Thorakale Neoplasien*
WZ 9	Kenntnisse der wesentlichen thorakalen Neoplasien und deren Prognose in Abhängigkeit des Stadiums und Behandlungsfortschritts
TK26	Grundlegende Kenntnisse der Bedeutung gezielt therapierbarer Mutationen für die Prognose
PF15	*Level I: interventionelle Endoskopie bei Tumorverlegung und/oder Blutung der Atemwege
*11.10*	*Andere pulmonale Probleme*
TK27	Atelektasen
PF16	*Level I: Therapie von Atelektasen durch Endoskopie bei respiratorisch instabilen Patienten

Anmerkung: Level-I- bis -III-Angaben sind zur Hervorhebung mit einem * versehen

#### Grundlagen und Standards.

Erforderlich sind grundlegende Kenntnisse der Lungenanatomie, -physiologie und -pathophysiologie, Kenntnisse der für die klinische Akut- und Notfallmedizin relevanten akuten und chronischen Lungenerkrankungen, der Verfahren zur Diagnosestellung (inkl. Materialentnahme) und Kenntnisse der Therapie akuter Funktionsstörungen des Organs. Das Erkennen eines A(temwegs)- und B(eatmungs)-Problems soll gewährleistet sein und die Diagnose einer akuten Lungenerkrankung inkl. der Lungenarterienembolie soll selbständig gestellt werden. Notwendige Therapieverfahren, wie Maskenbeatmung, nichtinvasive Beatmung, Notfallintubation und invasive Beatmung, sowie grundlegende Beatmungseinstellungen sollen selbstständig durchgeführt werden können. Grundlegende Kenntnisse der Lungen- und Pleurasonographie sollen vorhanden sein, ebenso wie grundlegende Kenntnisse der Echokardiographie und der Befundung konventioneller Röntgenaufnahmen des Thorax.

#### Das soll gewusst und gekonnt werden.

- Er soll selbstständig einen **Pneumothorax/Hämatothorax** diagnostizieren, Kenntnisse der klinischen und radiologischen Zeichen einer Spannungskomponente bei Pneumothorax haben und stadienadaptiert therapieren können (inkl. Anlage einer Thoraxdrainage).
- Die Indikationsstellung für die diagnostische und therapeutische **Bronchoskopie** (z. B. bei der Aspiration von Fremdkörpern, aber auch z. B. bei Polytrauma) soll beherrscht werden; ggf. kann die Durchführung in Kooperation mit einem Pneumologen erfolgen.
- Bei **respiratorischem Versagen** jedweder Art und Genese sind grundlegende Kenntnisse der Pathophysiologie und der Behandlung zur Sicherstellung der Oxygenierung und Dekarboxylierung erforderlich.
- Bei **obstruktiven Ventilationsstörungen** sind Kenntnisse zu Indikationen und Kontraindikationen einer antiobstruktiven Therapie erforderlich, ebenso Kenntnisse und praktische Erfahrungen mit verschiedenen Möglichkeiten der Beatmung (nichtinvasiv, invasiv). Die Fähigkeit zur Einleitung einer nichtinvasiven Beatmung und Kenntnisse grundsätzlicher Beatmungseinstellungen zur Oxygenierung und Dekarboxylierung, insbesondere zur Therapie der Hyperkapnie, sollen vorhanden sein. Kenntnisse des typischen Verlaufs, der Akut- und Langzeitprognose und akuter und mittel-/langfristiger therapeutischer Optionen bei obstruktiven Ventilationsstörungen sind erforderlich.
- Bei **restriktiven Ventilationsstörungen** sind Kenntnisse erforderlich zu Indikationen und Kontraindikationen einer Beatmungstherapie, bezüglich des typischen Verlaufs, der Akut- und Langzeitprognose und akuter und mittel-/langfristiger therapeutischer Optionen.
- Bei **Lungenblutungen** sollen im Kontext der Grunddiagnose gekannt und beherrscht werden: Ätiologie und Pathogenese, Ersteinschätzung, therapeutische Optionen (in interdisziplinärer Zusammenarbeit mit interventionellen Radiologen und Thoraxchirurgen) sowie deren stadienadaptierte Therapie zur akuten Sicherung des Gasaustauschs.
- **Interstitielle Lungenerkrankungen**, Vaskulitiden, Lungenblutungen und pulmonal-arterielle Hypertonie sollen in enger Zusammenarbeit mit dem Pneumologen und/oder Rheumatologen behandelt werden können.
- Er soll **Lungenarterienembolien** erkennen und behandeln können und Kenntnisse der Pathophysiologie und der Risikostratifizierung haben. Ebenso sind eingehende Kenntnisse der Therapieoptionen bis hin zu interventionellen Therapieverfahren und der Indikation zur venoarteriellen ECMO erforderlich.
- **Entzündliche und infektiöse Lungenerkrankungen** (Bronchitis, Bronchiolitis, Pneumonien mit und ohne Erguss/Empyem und schwere Pneumonien mit septischem Verlauf) sind häufige akutmedizinische Krankheitsbilder. Hier soll die Fähigkeit zur risikoadaptierten (Differenzial‑)Diagnosestellung und oft rasch notwendigen Therapieeinleitung, v. a. einer effektiven kalkulierten antiinfektiven Therapie, vorhanden sein.
- Wichtig sind auch Kenntnisse häufiger und akuter Komplikationen von **malignen Erkrankungen** der Lunge und des Thorax sowie von Lungenmetastasen und deren Prognose.

### 11.12. Rheumatologische Aspekte der Akut- und Notfallmedizin (Tab. 18)

**Table 18:** 

*18.1*	*Entzündliche Gelenkerkrankungen (z.* *B. rheumatoide Arthritis, Psoriasisarthritis und Spondyloarthritis)*
WZ 1	Erwerb der Kenntnisse und praktischen Fähigkeiten in der Diagnostik entzündlicher Gelenkerkrankungen
TK 1	Grundkenntnisse der immunologisch vermittelten Gelenkentzündung
TK 2	Klinische Symptomatik immunologisch bedingter, infektiologischer und kristallinduzierter Arthritiden
TK 3	Grundzüge der laborchemischen und immunologischen Diagnostik
TK 4	Grundzüge der bildgebenden Diagnostik
TK 5	Grundzüge einer immunsuppressiven/antiinflammatorischen/immunmodulatorischen Therapie, ihrer möglichen – v. a. infektiologischen – Komplikationen sowie deren Maskierung durch ebendiese Immunsuppression
PF 1	*Level III: Fähigkeit zur selbständigen klinischen Untersuchung und Diagnostik zum Erkennen einer entzündlichen Gelenkerkrankung
PF 2	*Level III: Suche nach und Erkennen von Gichttophi
PF 3	*Level II: Einordnung der bildgebenden, laborchemischen und immunologischen Befunde und Ultraschalldiagnostik einer Arthritis
PF 4	*Level III: Erkennen von Warnhinweisen („*red flags*“) für komplikative Verläufe: septische Arthritis, Nervenkompressionssyndrome, schwere extraartikuläre Manifestationen entzündlicher Gelenkerkrankungen, spinale Komplikationen von rheumatoider Arthritis (RA) und Spondylarthropathie (SpA)
BV 1	Fähigkeit zur Zusammenarbeit mit Rheumatologen in der Patientenbetreuung und Diagnostik
*18.2*	*Kollagenosen (z.* *B. systemischer Lupus erythematodes, Myositis, Mischkollagenose, Sjögren-Syndrom)*
WZ 2	Erwerb der Grundkenntnisse und praktischen Fähigkeiten in der Diagnostik von Kollagenosen
TK 6	Grundkenntnisse der immunologisch vermittelten Entzündung bei Kollagenosen
TK 7	Kenntnisse typischer klinischer Befunde von Kollagenosen
TK 8	Grundzüge der laborchemischen und immunologischen Diagnostik
TK 9	Grundzüge der bildgebenden Diagnostik
PF 5	*Level III: Erkennen der klinischen Symptome und deren Bedeutung im Kontext von Kollagenosen (z. B. Raynaud-Phänomen, Sklerodaktylie, Tabaksbeutelmund, Schmetterlingserythem, vaskulitische Hautveränderungen, Schluckstörung, Dyspnoe, Nekrosen, Polyserositis, Thrombosen/Embolien, Fieber, Nierenversagen, Endokarditis, HyperCKämie, Hämatemesis, Hämoptysen, Hämatochezie, Pankreatitis, Rechtsherzinsuffizienz)
PF 6	*Level III: richtiges Einordnen von „*red flags*“ (z. B. Fieber, Dyspnoe, Bewusstseinstrübung) in die Differenzialdiagnose möglicher Therapiekomplikationen oder spezifischer Organmanifestationen wie Nieren‑, Herz‑, Lungen- oder ZNS-Beteiligung
BV 2	Fähigkeit zur Zusammenarbeit mit Rheumatologen in der Patientenbetreuung und Diagnostik, ggfs. mit den Fachgebieten Kardiologie, Pneumologie und Nephrologie
*18.3*	*Antiphospholipidantikörpersyndrom (APS)*
WZ 3	Erwerb der Grundkenntnisse und praktischen Fähigkeiten in der Diagnostik des APS
TK 10	Grundkenntnisse der Labordiagnostik des APS
TK 11	Kenntnis möglicher klinischer Symptome (Lungenembolie, Apoplex, Extremitätennekrosen, Schwangerschaftskomplikationen [z. B. rezidivierende Aborte])
TK 12	Kenntnis der Maximalvariante des APS („*catastrophic*“* APS, *CAPS)
PF 7	*Level II: Erkennen klinischer Präsentationen des APS; Akutdiagnostik und deren Interpretation (Lupusantikoagulans, Autoantikörper)
PF 8	*Level I: Indikationsstellung für immunologische/extrakorporale Verfahren bei CAPS
BV 3	Fähigkeit zur Zusammenarbeit mit Rheumatologen und Hämostaseologen in der Patientenbetreuung und Diagnostik
*18.4*	*Polymyalgia rheumatica (PMR)*
WZ 4	Erwerb der Kenntnisse und praktischen Fähigkeiten von Klinik, Diagnostik und Akuttherapie einer PMR
TK 13	Sichere Diagnostik einer PMR
TK 14	Erkennen der Komorbidität „Riesenzellarteriitis“ (inklusive Arteriitis temporalis, hier v. a. deren Warnsignale wie Angina masseterica, Amaurosis fugax)
TK 15	Therapie der PMR
PF 9	*Level II: Interpretation des Labors
PF 10	*Level I: Sonographie der Bizepssehne und Bursen des Schultergelenks
PF 11	*Level II: Therapie
BV 4	Fähigkeit zur Zusammenarbeit mit Rheumatologen in der Patientenbetreuung, Diagnostik und Therapie
*18.5*	*Riesenzellarteriitis (inkl. Arteriitis temporalis)*
WZ 5	Erwerb der Grundkenntnisse und praktischen Fähigkeiten in der klinischen Symptomatik und Diagnostik einer Riesenzellarteriitis (inklusive Arteriitis temporalis, hier v. a. deren Warnsignale wie Angina masseterica, Amaurosis fugax)
TK 16	Grundkenntnisse der immunologisch vermittelten Gefäßentzündung
TK 17	Grundzüge der laborchemischen und bildgebenden Diagnostik (Gefäßsonographie)
TK 18	Grundkenntnisse der Therapie
TK 19	Erkennen möglicher organbedrohender Situationen (z. B. Erblindung und Gefäßdissektion und deren Warnsignale wie Angina masseterica, Amaurosis fugax)
PF 12	*Level I: Grundzüge in der Einordnung der laborchemischen Befunde
PF 13	*Level I: Grundzüge in der Einordnung der Bildgebung; duplexsonographische Grundkenntnisse (Erkennen eines *Halo*)
BV 5	Fähigkeit zur Zusammenarbeit mit Rheumatologen in der Patientenbetreuung und Diagnostik, ggf. mit den Fachgebieten Augenheilkunde und Neurologie
*18.6*	*Kleingefäßvaskulitiden (ANCA-assoziierte Vaskulitiden)*
WZ 6	Erwerb der Grundkenntnisse und praktischen Fähigkeiten in der klinischen Symptomatik und Diagnostik einer Kleingefäßvaskulitis (ANCA-assoziierte Vaskulitis)
TK 20	Grundkenntnisse der immunologisch vermittelten Gefäßentzündung
TK 21	Erkennen möglicher organbedrohender Situationen, v. a. Hämoptysen, Nierenversagen
TK 22	Grundzüge der laborchemischen und bildgebenden Diagnostik, der Interpretation eines Urinsediments
PF 14	*Level I: Grundzüge in der Einordnung der laborchemischen Befunde
PF 15	*Level I: Grundzüge der Organdiagnostik
PF 16	*Level I: Indikationsstellung zur immunsuppressiven Therapie
PF 17	*Level I: Indikationsstellung zu extrakorporalen Verfahren (z. B. Plasmapherese)
BV 6	Fähigkeit zur Zusammenarbeit mit Rheumatologen in der Patientenbetreuung und Diagnostik, ggf. mit den Fachgebieten Kardiologie, Pneumologie und Nephrologie
*18.7*	*Autoinflammatorische Syndrome (z.* *B. adultes Still-Syndrom, Makrophagenaktivierungssyndrom [Synonym: hämophagozytische Lymphohistiozytose], familiäres Mittelmeerfieber, „drug-reaction with eosinophilia and systemic symptoms“ [DRESS])*
WZ 7	Erwerb der Grundkenntnisse und praktischen Fähigkeiten in der Diagnostik unklarer Fiebersyndrome
TK 23	Grundkenntnisse der autoinflammatorischen Syndrome als Ursachen eines unklaren Fiebers
TK 24	Grundkenntnisse der Labordiagnostik (insbesondere Ferritin)
TK 25	Grundkenntnisse etablierter Scoringsysteme bei Makrophagenaktivierungssyndrom (z. B. HScore)
PF 18	*Level II: Grundzüge in der Einordnung der laborchemischen Befunde
PF 19	*Level I: Indikationsstellung zur immunsuppressiven Therapie
BV 7	Fähigkeit zur Zusammenarbeit mit Rheumatologen in der Patientenbetreuung und Diagnostik, ggf. mit dem Fachgebiet Hämatologie
*18.8*	*Infektionen unter immunsuppressiver Therapie*
WZ 8	Erkennen von Infektionen unter einer antiinflammatorischen/immunsuppressiven/immunmodulatorischen Therapie; Therapie der Infektionserkrankung und Handhabung der immunsuppressiven Therapie; Kenntnisse über die Besonderheiten der Diagnostik und Therapie bei Infektionen unter immunsuppressiver Therapie
TK 26	Diagnostik der Infektion
TK 27	Einleitung einer antiinfektiven Therapie
TK 28	Handhabung der immunsuppressiven Therapie
TK 29	Kenntnisse über Möglichkeiten der Chemoprophylaxe unter Immunsuppression (z. B. *Pneumocystis-jirovecii*-Pneumonie [PjP])
PF 20	*Level III: Management der Infektion, auch opportunistischer Infektion
PF 21	*Level I: Indikationsstellung zur Chemoprophylaxe bei Immunsuppression
BV 8	Fähigkeit zur Zusammenarbeit mit Rheumatologen in der Diagnostik und Therapieplanung, ggf. mit den Fachgebiet Infektiologie

Anmerkung: Level-I- bis -III-Angaben sind zur Hervorhebung mit einem * versehen

#### Grundlagen und Standards.

Entzündlich rheumatische Erkrankungen sind durch autoimmunologische bzw. autoinflammatorische Prozesse des Immunsystems gekennzeichnet, die mit einer entkoppelten immunologischen Entzündungsreaktion und folgender Zerstörung des körpereigenen Gewebes einhergehen. Zusätzlich werden auch nichtentzündliche Gelenkerkrankungen wie die Arthrose der Rheumatologie zugeordnet.

#### Das soll gewusst und gekonnt werden.

- Entscheidend für die bestmögliche Betreuung von Patienten mit rheumatologischen Erkrankungen in der Notaufnahme ist eine **fundierte Diagnostik**.
- Zu den **akutmedizinisch relevanten rheumatologischen Erkrankungen** zählen entzündliche Gelenkerkrankungen, Kollagenosen, das Antiphospholipidantikörpersyndrom (APS), die Polymyalgia rheumatica, die Riesenzellarteriitis (inkl. Arteriitis temporalis), die Kleingefäßvaskulitiden (ANCA-assoziierte Vaskulitiden) und autoinflammatorische Syndrome inkl. kristallinduzierter Arthritiden.
- Die antiinflammatorische, immunsuppressive und immunmodulatorische **Therapie rheumatologischer Erkrankungen** birgt für die Patienten die Gefahr infektiöser Komplikationen. Dies erfordert Kenntnisse über die Besonderheiten der Diagnostik und Therapie bei Infektionen unter immunsuppressiver Therapie.

### 11.13. Toxikologische Aspekte der Akut- und Notfallmedizin (Tab. 19)

**Table 19:** 

WZ 1	Erwerb der Kenntnisse in klinischer Toxikologie, die für eine selbständige Diagnostik und (An‑)Behandlung der häufigsten und gefährlichsten Intoxikationen nötig sind
TK 1	Kenntnis vital bedrohlicher Intoxikationen
TK 2	Verstehen des zeitlichen Verlaufs von Intoxikationen
TK 3	Rolle der Giftnotrufzentralen
TK 4	Logistische Herausforderungen bei intoxikierten Patienten
*19/1*	*Aufnahme des intoxikierten Patienten*
TK 5	Kenntnis der Besonderheiten bei Intoxikierten
TK 6	Risikoabschätzung für Patient und Personal
TK 7	Verletzungen bei Intoxikierten
TK 8	EKG-Beurteilung
TK 9	Pupillenbefund bei verschiedenen Intoxikationen
TK 10	Eigenschutz/Dekontamination
TK 11	Grundkenntnisse im Umgang mit aggressiven Patienten/Deeskalation
PF 1	*Level III: fokussierte körperliche Untersuchung
PF 2	*Level III: Umgang mit aggressiven Patienten
PF 3	*Level III: fokussierte Anamnese
PF 4	*Level II: EKG-Beurteilung
BV 1	Fähigkeit zur Deeskalation
BV 2	Fähigkeit zum einfühlsamen Umgang mit Patienten nach Suizidversuch
*19/2*	*Laboruntersuchungen bei intoxikierten Patienten*
TK 12	Basislabor
TK 13	Kenntnisse von Laborveränderungen bei bestimmten Intoxikationen
TK 14	Urinuntersuchung mittels Gaschromatographie mit Massenspektrometriekopplung (U-GCMS)
*19/3*	*Toxidrome*
TK 15	Kenntnis des sympathomimetischen Syndroms
TK 16	Kenntnis des anticholinergen Syndroms
TK 17	Kenntnis des Opiatsyndroms
TK 18	Kenntnis des Serotoninsyndroms
TK 19	Kenntnis des cholinergen Syndroms
PF 5	*Level III: Erkennen von o. g. Syndromen
PF 6	*Level II: toxidromspezifische Therapie
*19/4*	*Antidote*
TK 20	Überblick über die wichtigsten Antidote und ihrer Dosierung
TK 21	Nebenwirkungen der Antidote
*19/5*	*Giftelimination*
TK 22	Primäre Giftelimination, insbesondere Einsatz von Aktivkohle
TK 23	Sekundäre Giftelimination, Mehrfachdosis Aktivkohle (MDAC)
TK 24	Weitere Möglichkeiten zur Giftelimination und deren Indikation (Gastroskopie, Magenspülung, antegrade Darmspülung („whole bowel irrigation“, WBI), Dialyse
TK 25	Kenntnis von Pharmakobezoarbildnern
PF 7	*Level III: Erkennen der Kontraindikationen für eine Gabe von Aktivkohle
19/6	*Verlegung/Entlassung des intoxikierten Patienten*
TK 26	Kenntnisse der Voraussetzungen für eine sichere Entlassung
TK 27	Kenntnisse der Indikationen für eine Verlegung auf die IMC/ITS
PF 8	*Level III: Erstellen strukturierter Verlegungs‑/Entlassbriefe
*19/7*	*Spezielle Toxikologie*
WZ *2*	Erlangung der Kenntnisse für die selbständige Versorgung von Patienten mit u. g. Intoxikationen
TK 28	Kenntnis über u. g. Intoxikationen (*19.8–19.13*)
TK 29	Kenntnisse der Risiken bei u. g. Intoxikationen
PF 9	*Level III: Fähigkeit zur selbstständigen Indikationsstellung einer zielführenden Diagnostik sowie zur selbstständigen Therapiedurchführung bei Intoxikationen, soweit es im Rahmen der Akutmedizin möglich ist
*19/8*	*Medikamentenintoxikationen – häufige Vergiftungen*
TK 30	Psychopharmaka, insbesondere Serotoninwiederaufnahmehemmer (SSRI)
TK 31	Paracetamol, inkl. Umgang mit dem Rumack-Matthew-Nomogramm
TK 32	Trizyklische Antidepressiva (TZA)
*19/9*	*Seltenere, aber gefährliche Vergiftungen*
TK 33	β‑Blocker (u. a. „high dose insulin euglycemic therapy“ [HIET], va-ECMO)
TK 34	Kalziumantagonisten
TK 35	Salizylate
TK 36	Zyanid (auch inhalativ)
*19/10*	*Inhalative Intoxikationen (außer Drogen)*
TK 37	Kohlenmonoxid (CO; [88])
*19/11*	*Toxische Alkohole*
TK 38	Methanol
TK 39	Ethylenglykol
TK 40	Isopropanol und Azeton
*19/12*	*Intoxikationen mit Drogen*
TK 41	Alkohol (Äthylalkohol)
TK 42	Opiate
TK 43	Synthetische Cannabinoide
TK 44	Kokain
TK 45	Halluzinogene
TK 46	γ‑Hydroxybuttersäure (GHB)
TK 47	Amphetamin
TK 48	Methamphetamin
TK 49	3,4-Methylendioxy-N-methylamphetamin (MDMA, Ecstacy)
*19/13*	*Intoxikation mit Säuren und Laugen*
TK 50	Verständnis des Unterschiedes bei Ingestionen von Säuren oder Laugen
TK 51	Rationales Vorgehen bei diesen Intoxikationen

Anmerkung: Level-I- bis -III-Angaben sind zur Hervorhebung mit einem * versehen

#### Grundlagen und Standards.

Relevant sind diejenigen Kenntnisse in klinischer Toxikologie, die für eine selbstständige Diagnostik und (Primär‑)Behandlung der häufigsten und gefährlichsten Intoxikationen nötig sind. Bei der zielführenden Diagnostik und Therapie der zahlreichen und komplexen Medikamentenintoxikationen empfiehlt sich die Kooperation mit den Giftnotrufzentralen.

#### Das soll gewusst und gekonnt werden.

- **Toxidrome, Antidote, primäre Giftelimination**: Der Akut- und Notfallmediziner benötigt insbesondere das Wissen um Toxidrome (sympathomimetisch, anticholinerg, Opiat, Serotonin, cholinerg) und der toxidromspezifischen Therapie.
- Der Akut- und Notfallmediziner soll auch einen **Überblick über die wichtigsten Antidote und deren Dosierung sowie über die Möglichkeiten der primären Giftelimination **haben.

## Abkürzungen

AAPV: Allgemeine ambulante Palliativversorgung
ABCDE: „*Airway, breathing, circulation, disability, exposure/environment*“
ABC-Schema: („*Abdomen*“, „*Breath*“ [Thorax], „*Cardiac*“)-Schema
ABS: Antibiotic Stewardship
ACS: Akutes Koronarsyndrom („*acute coronary syndrome*“)
AKI: Akutes Nierenversagen („*acute kidney injury*“), akute Nierenfunktionseinschränkung
ALI: Akute Extremitätenischämie („*acute limb ischaemia*“)
ALS: *Advanced Life Support*
ANCA: Antineutrophile zytoplasmatische Antikörper („*anti-neutrophil cytoplasmatic antibody“*)
APS: Antiphospholipidantikörpersyndrom
ASD: Vorhofseptumdefekt („*atrial septal defect*“)
AWMF: Arbeitsgemeinschaft der Wissenschaftlichen Medizinischen Fachgesellschaften e. V.
BÄK: Bundesärztekammer („*german medical association*“)
BAL: Bronchoalveoläre Lavage
BDI: Berufsverband Deutscher Internistinnen und Internisten e. V.
BGB: Bürgerliches Gesetzbuch
BV: Beruflich-professionelles Verhalten
CAPS: „*Catastrophic antiphospholipid antibody syndrome*“
CAR‑T Cells: „*Chimeric antigen receptor T cells*“
cCT: Kraniale Computertomographie („*cranial computer tomography*“)
CKD: Chronische Nierenerkrankung („*chronic kidney disease*“)
CLI: Kritische Extremitätenischämie („*critical limb ischaemia*“)
cMRT: Kraniale Magnetresonanztomographie („*cranial magnetic resonance tomography*“)
CO: Kohlenmonoxid („carbon monoxide“)
COPD: Chronisch-obstruktive Bronchopneumopathie („*chronic obstructive pulmonary dsease*“)
COVID-19: Coronaviruskrankheit 2019
CT: Computertomographie („*computer tomography*“)
DGA: Deutsche Gesllschaft für Angiologie – Gesellschaft für Gefäßmedizin e.V.
DGIIN: Deutsche Gesellschaft für Internistische Intensivmedizin und Notfallmedizin e. V. („*german society of medical intensive care and emergency medicine*“)
DGIM: Deutsche Gesellschaft für Innere Medizin e. V.
DGINA: Deutsche Gesellschaft Interdisziplinäre Notaufnahme e. V./Deutsche Gesellschaft Interdisziplinäre Notfall- und Akutmedizin e. V.
DGK: Deutsche Gesellschaft für Kardiologie – Herz- und Kreislaufforschung e. V.
DGUM: Deutsche Gesellschaft für Ultraschall in der Medizin e. V.
DKA: Diabetische Ketoazidose
*DRESS*: *„Drug-reaction with eosinophilia and systemic symptoms“*
ECLS: „*Extracorporeal life support*“
ECMO: Extrakorporale Membranoxygenierung („*extracorporeal membrane oxygenation*“)
eCPR: Extrakorporale kardiopulmonale Reanimation (*„extracorporeal cardiopulmonary resuscitation“*)
EKG: Elektrokardiogramm
ERC(P): Endoskopische retrograde Cholangio(pankreatiko)graphie.
ESC: *„European Society of Cardiology“* (Europäische Gesellschaft für Kardiologie)
ESICM: European Society of Intensive Care Medicine
FEEL: „*Focused echocardiography in emergency life support*“
FEWP: Fachlich empfohlener Weiterbildungsplan
G-BA: Gemeinsamer Bundesausschuss
GHB: γ‑Hydroxybuttersäure
GI: Gastrointestinal
HIET: „*High dose insulin euglycemic therapy*“
HKL: Herzkatheterlabor
HKU: Herzkatheteruntersuchung
HIV: Humanes Immundefizienzvirus
HUS: Hämolytisch-urämisches Syndrom
ICD: Implantierbarer Kardioverter/Defibrillator („*implantable cardioverter-defibrillator*“)
IMC: *„Intermediate Care“*
ITS: Intensivstation
LAA: Linkes Vorhofohr
LAE: Lungenarterienembolie
MDAC: Mehrfachdosis Aktivikohle („*multiple-dose activated charcoal*“)
MDMA: Ecstacy (3,4-Methylendioxy-N-methylamphetamin)
MODS: Multiples Organdysfunktionssyndrom („*mulitple organ dysfunction syndrome*“)
MRGN: Multiresistente gramnegative Bakterien
MRT: Magnetresonanztomographie („*magnetic resonance tomography*“)
MRSA: Methicillinresistenter *Staphylococcus aureus*
(M-)WBO: (Muster‑)Weiterbildungsordnung
NNR: Nebennierenrinde
NSC: „Nonspecific complaints“
NSTEMI: Nicht-ST-Strecken-Hebungs-Infarktinfarkt („*non-ST-elevation myocardial infarction*“)
PCI: Perkutane Koronarintervention („*percutaneous coronary intervention*“)
PEEP: Positiver endexspiratorischer Druck („*positive endexpiratory pressure*“)
PEP: Postexpositionsprophylaxe
PF: Praktische Fähigkeiten
PFO: Persistierendes Foramen ovale
PH: Pulmonale Hypertonie
PjP: *Pneumocystis-jirovecii*-Pneumonie
PMR: Polymyalgia rheumatica
POCT: „*Point of care testing*“
PPI: Protonenpumpeninhibitor(en)
PVK: Peripherer Venenkatheter
QM: Qualitätsmanagement
RA: Rheumatoide Arthritis
RPGN: Rapid progressive Glomerulonephritis
RSV: Humanes Respiratorisches Synzytial-Virus („*human orthopneumovirus*“)
SAPV: Spezialisierte ambulante Palliativversorgung
SARS-CoV‑2: Schweres-akutes-Atemwegssyndrom-Coronavirus Typ 2 („*severe acute respiratory *syndrome* coronavirus type 2*“)
SBP: Spontan-bakterielle Peritonitis
SGLT‑2: Natrium-Glukose-Cotransporter 2
SIADH: Syndrom der inadäquaten ADH-Sekretion
SIN‑I,II: Sonographie in der internistischen Notfall- und Intensivmedizin I, II
SOP: „*Standard operating procedure“*
SpA: Spondylarthropathie
SSRI: Selektive Serotonin-reuptake-Inhibitoren
STEMI: ST-Strecken-Hebungs-Infarkt („*ST-elevation myocardial infarction*“)
TK: Theoretische Kenntnisse
TMA: Thrombotische Mikroangiopathie
TMU: Temporäres mechanisches Unterstützungssystem
TEE: Transösophageale Echokardiographie („*transesophageal echocardiography*“)
TTE: Transthorakale Echokardiographie („*transthoracical echocardiography*“)
TTP: Thrombotisch-thrombozytopenische Purpura
TVT: Tiefe Venenthrombose
TZA: Trizyklische Antidepressiva
UEMS: *European Union of Medical Specialists*
va-ECMO: Venoarterielle extrakorporale Membranoxygenierung
VRE: Vancomycinresistente Enterokokken
VSD: Ventrikelseptumdefekt („*ventricular septal defect*“)
vv-ECMO: Venovenöse extrakorporale Membranoxygenierung
WBO: Weiterbildungsordnung
WBI: Antegrade Darmspülung („whole bowel irrigation“)
WZ: Weiterbildungsziel
ZVK: Zentralvenöser Katheter
Z‑WB: Zusatz-Weiterbildung
